# Malaria parasite heme biosynthesis promotes and griseofulvin protects against cerebral malaria in mice

**DOI:** 10.1038/s41467-022-31431-z

**Published:** 2022-07-12

**Authors:** Manjunatha Chandana, Aditya Anand, Sourav Ghosh, Rahul Das, Subhashree Beura, Sarita Jena, Amol Ratnakar Suryawanshi, Govindarajan Padmanaban, Viswanathan Arun Nagaraj

**Affiliations:** 1 Infectious Disease Biology, Institute of Life Sciences, Bhubaneswar, 751023 Odisha India; 2 School of Biotechnology, Kalinga Institute of Industrial Technology, Bhubaneswar, 751024 Odisha India; 3 Regional Centre for Biotechnology, Faridabad, 121001 Haryana India; 4 Department of Biochemistry, Indian Institute of Science, Bangalore, 560012 Karnataka India

**Keywords:** Mechanisms of disease, Parasite biology, Malaria

## Abstract

Heme-biosynthetic pathway of malaria parasite is dispensable for asexual stages, but essential for mosquito and liver stages. Despite having backup mechanisms to acquire hemoglobin-heme, pathway intermediates and/or enzymes from the host, asexual parasites express heme pathway enzymes and synthesize heme. Here we show heme synthesized in asexual stages promotes cerebral pathogenesis by enhancing hemozoin formation. Hemozoin is a parasite molecule associated with inflammation, aberrant host-immune responses, disease severity and cerebral pathogenesis. The heme pathway knockout parasites synthesize less hemozoin, and mice infected with knockout parasites are protected from cerebral malaria and death due to anemia is delayed. Biosynthetic heme regulates food vacuole integrity and the food vacuoles from knockout parasites are compromised in pH, lipid unsaturation and proteins, essential for hemozoin formation. Targeting parasite heme synthesis by griseofulvin—a FDA-approved antifungal drug, prevents cerebral malaria in mice and provides an adjunct therapeutic option for cerebral and severe malaria.

## Abstract (web summary)

Malaria parasite heme pathway is non-essential for blood stages, but essential for mosquito and liver stages. Here, the authors show that heme pathway promotes cerebral pathogenesis by enhancing hemozoin formation and that targeting it with griseofulvin inhibits cerebral malaria in mice.

## Introduction

Malaria remains a major concern of morbidity and mortality, especially with the emerging parasite resistance to artemisinin-based combination therapies (ACTs) and mosquito resistance to insecticides^1^. According to World Health Organization (WHO), 241 million cases and 627,000 malaria deaths occurred in 2020^1^. Of the five *Plasmodium* species causing human malaria, *Plasmodium falciparum* (*Pf*) is the deadliest one responsible for more than 90% of the infections. The clinical manifestations of *Pf* malaria vary from mild (uncomplicated malaria) to severe (complicated malaria). Uncomplicated malaria is characterized by fever, headache, nausea, chills and mild anemia. Complicated malaria is categorized by the existence of at least one criterion of disease severity that includes respiratory distress, metabolic acidosis, pulmonary edema, severe anemia, jaundice, renal failure or neurological complications like impaired consciousness, convulsions etc. The typical outcome of severe malaria is multi-organ failure and/or cerebral malaria (CM) of which, CM is the most severe neurological complication with high mortality. About one-third of the patients recovering from CM show long-term neurocognitive impairments^2–4^.

Our current understanding on CM comes from a few post-mortem studies of human CM (HCM) and a large number of experimental CM (ECM) studies performed in mouse models^5,6^. Although rodent parasites lack orthologs for *Pf* erythrocyte membrane protein 1 - a protein family that is transported to RBC membrane and primarily associated with parasite sequestration, the molecular mechanisms and cellular machinery underlying cerebral pathogenesis and parasite virulence are conserved^7^. There were earlier discrepancies on the extent of using ECM as a model for HCM, especially in the context of the sequestration of parasitized RBCs (pRBCs) in brain microvasculature. HCM is characterized by strong cytoadherence and dense sequestration of large numbers of pRBCs that can obstruct substantial lengths of cerebral microvasculature^8^, in contrast to irregular distribution of pRBCs in ECM that seem to be mechanically trapped^9,10^. However, several studies have suggested significant analogies between HCM and ECM in terms of cerebral and neurovascular pathology. There is a consensus on increased permeability of blood-brain barrier (BBB), brain capillary occlusions, parasitized-red blood cells (pRBCs) accumulation in brain microvasculature, leukocyte infiltration, and endothelial activation with dysregulated inflammation and aberrant host-immune responses. All these lead to BBB disruption, intracerebral hemorrhages, ischemia, edema, increased intracranial pressure, axonal damage and demyelination, culminating in the dysfunction of central nervous system^5,6,11^.

Cerebral pathology arises due to a complex interplay of molecular events triggered by various host- and parasite-derived factors. The synchronous growth of the asexual parasites in red blood cells (RBCs) and the associated schizogony result in the release of pathogen-associated molecular patterns (PAMPs) such as hemozoin (Hz), glycosylphosphatidylinositiol, parasite DNA and RNA, and danger-associated molecular patterns (DAMPs) such as heme, uric acid, and extracellular vesicles^12,13^. Hz and its precursor heme play a central role in CM pathogenesis^14–21^. The asexual stage parasites endocytose host hemoglobin (Hb) and digest it in the food vacuole (FV). The toxic free heme released during this process is detoxified into Hz by heme detoxification protein (HDP) that undergoes a circuitous trafficking and abundantly present in the infected RBC cytosol than parasite FVs^22^. Although the rate of HDP-mediated Hz formation is much higher, autocatalytic^23^, histidine rich protein (HRP)-mediated^24^ and lipid-driven mechanisms^25,26^ for Hz formation have also been described. There is a positive correlation of Hz released into the circulation and phagocytosed by the circulating phagocytic cells with disease severity in children and adults^27–29^. Similarly, plasma free heme is associated with disease severity^30,31^. Free heme is cytotoxic to endothelial cells and it can increase the expression of adhesion molecules, induce NLRP3 inflammasome and IL-1β secretion, and activate polymorphonuclear cells. The only treatment option for CM is parenteral administration of artemisinin derivatives or quinine with supportive therapies. However, the fatality due to CM remains high despite the parasite clearance^2–4,32^. The molecular mechanisms underlying CM pathogenesis need to be understood for developing adjunct therapies.

Malaria parasite synthesizes heme de novo despite the ability of asexual stages to access host Hb-heme^33^. The parasite heme pathway is compartmentalized in mitochondrion, apicoplast and cytosol, and heme is eventually synthesized in the mitochondrion. Our earlier study with *P. berghei* (*Pb*) aminolevulinate synthetase (ALAS) and ferrochelatase (FC) knockouts (KOs) generated for the first and last enzymes, demonstrated that the parasite pathway is dispensable for growth in asexual stages, but essential for the development of sporozoites in mosquitoes and pre-erythrocytic stages in liver. Moreover, FCKO parasites can utilize host Hb-heme for their survival in blood stages^34,35^. Subsequent studies in *Pf* using the KO parasites generated for ALAS, FC, apicoplast-localized porphobilinogen deaminase and cytosol-localized coproporphyrinogen oxidase, confirmed these findings with the suggestion that extracellular ALA acquired through new permeability pathways may also lead to parasite heme synthesis^36,37^. The conversion of extracellular ALA into protoporphyrin IX occurs in RBCs with the help of host enzymes, and heme is synthesized by parasite FC. Although de novo heme synthesis is non-essential and the asexual KO parasites can acquire heme/heme precursors from host RBCs and import some of the host enzymes, the parasite enzymes are expressed and heme synthesis occurs as evident from ^13^C/^14^C-ALA metabolic labeling studies^34–41^. We hypothesized that the parasite de novo heme pathway should have a physiological relevance in the asexual stages. Here, we show that de novo heme induces ECM pathogenesis by regulating Hz formation in the asexual stages, thus offering an answer to a long-standing question. We further provide an adjunct therapeutic option on the basis that griseofulvin - a well-known FDA-approved drug capable of inhibiting parasite heme synthesis, can prevent ECM in mice.

## Results

### Heme pathway KO parasite-infected mice are protected from ECM

**Fig. 1:**
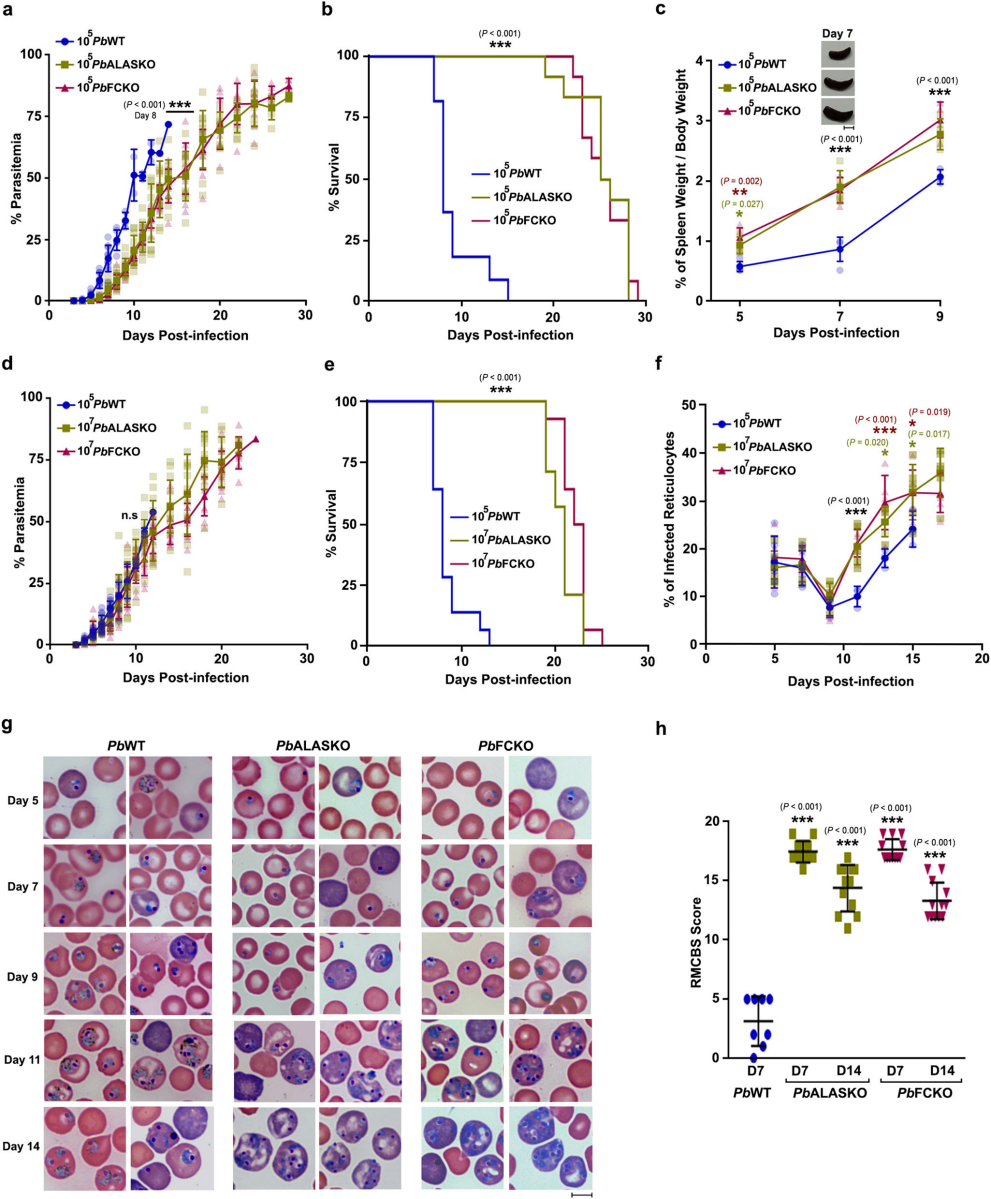
CM protection in heme pathway KO parasite-infected mice. **a** Growth analysis of *Pb*WT (*n* = 10), *Pb*ALASKO (*n* = 12) and *Pb*FCKO (*n* = 12) parasites in C57BL/6 mice. 10^5^ parasites were used to initiate *Pb*WT and *Pb*KO parasite infections. The data represent three different batches. (mean ± SD; ***P < 0.001, Two-way ANOVA). **b** Mortality curves of mice infected with *Pb*WT, *Pb*ALASKO and *Pb*FCKO parasites. The data represent the mice utilized for growth curve analysis (***P < 0.001, log-rank (Mantel–Cox) test). **c** Spleen weight of mice infected with *Pb*WT (*n* = 13), *Pb*ALASKO (*n* = 14) and *Pb*FCKO (*n* = 14) parasites. For each day, 3–5 mice from at least three different batches were included (mean ± SD; *P < 0.05, **P < 0.01, ***P < 0.001, Two-way ANOVA). Scale bar = 1 cm. **d** Growth analysis of *Pb*WT (*n* = 14), *Pb*ALASKO (*n* = 14) and *Pb*FCKO (*n* = 14) parasites in C57BL/6 mice. 10^5^ and 10^7^ parasites were used to initiate WT and KO parasite infections, respectively. The data represent four different batches (mean ± SD; n.s. not significant, Two-way ANOVA). **e** Mortality curves of mice infected with *Pb*WT, *Pb*ALASKO and *Pb*FCKO parasites. The data represent the mice utilized for growth curve analysis (***P < 0.001, log-rank (Mantel–Cox) test). **f** Percentage of infected reticulocytes in the parasitized red cells (mean ± SD; *P < 0.05, ***P < 0.001, Two-way ANOVA). The data represent six mice each for *Pb*WT, *Pb*ALASKO and *Pb*FCKO parasites. **g** Giemsa-stained images for peripheral blood smears prepared from tail vein blood of *Pb*WT and *Pb*KO parasite-infected mice. Reticulocytes could be identified by their distinct blue color. Images were captured using 100x objective. Scale bar = 5 μm. *n* = 4–6 independent experiments. **h** RMCBS score for mice infected with *Pb*WT (*n* = 8) and *Pb*KO (*n* = 12) parasites. *Pb*WT data represent the mice that succumbed to ECM (mean ± SD; ***P < 0.001, unpaired *t*-test; two-sided). For (**a**, **c**, **d**, **f**), individual data points are shown with the respective light shaded colors. Source data are provided as a Source Data file.

Our earlier work with *Pb* heme pathway KO parasites was carried out in outbred Swiss mice that do not develop ECM^34^. Here, we performed our studies in ECM-susceptible C57BL/6 inbred mouse strain by injecting 10^5^ asexual stage parasites. Assessment of the peripheral blood parasitemia showed 2–3 days delay in the growth of KO parasites with respect to the WT (Fig. 1a). Importantly, about 80% of the WT-infected mice succumbed to ECM within day 10 when the blood parasitemia was around 10–30%. The WT-infected mice that escaped from ECM died of anemia on day 12–16 post-infection. In contrast, mice infected with ALASKO and FCKO parasites were protected from ECM and they died because of anemia on day 20–30 (Fig. 1b). The delay in the growth of KO parasites was associated with an early increase in the spleen weight of infected mice (Fig. 1c), suggesting a better splenic clearance. A similar delay in the growth of KO parasites and the mortality of KO-infected mice due to anemia was observed in Balb/c mice that do not develop ECM (Supplementary Fig. 1a, b). It is known that the growth of heme pathway KO parasites differs slightly with respect to the genetic background of mouse (inbred/outbred) and a similar delay in growth is also reported for other heme pathway KO parasites^34,42,43^. To rule out the possibility that ECM protection is because of a delay in the increase in blood parasitemia, we performed growth analyses in C57BL/6 mice infected with 10^7^ ALASKO/FCKO parasites. While the growth of 10^7^ KO parasites in mice was comparable with 10^5^ WT parasites, KO-infected mice were once again protected from ECM (Fig. 1d, e). The mortality due to anemia was delayed by 6–10 days, and KO-infected mice could sustain a higher parasitemia for a prolonged period. There were no significant differences in the reticulocyte versus mature RBC preference between WT and KO parasites in the first 9 days, the duration in which ECM mortality occurred in WT-infected mice. However, KO parasites showed significantly increased reticulocyte preference and multiple infections in the reticulocytes during the later course of infections that represent anemic phase (Fig. 1f, g). The rapid murine coma and behavioral scale (RMCBS) score of 10^5^ WT parasite-infected mice that succumbed to ECM was below 5 on day 7 whereas, RMCBS score of 10^7^ KO parasite-infected mice was around 17 and 14 on day 7 and 14, respectively (Fig. 1h). For all the subsequent experiments, we initiated asexual infections by injecting 10^5^ WT and 10^7^ ALASKO/FCKO parasites.

**Fig. 2:**
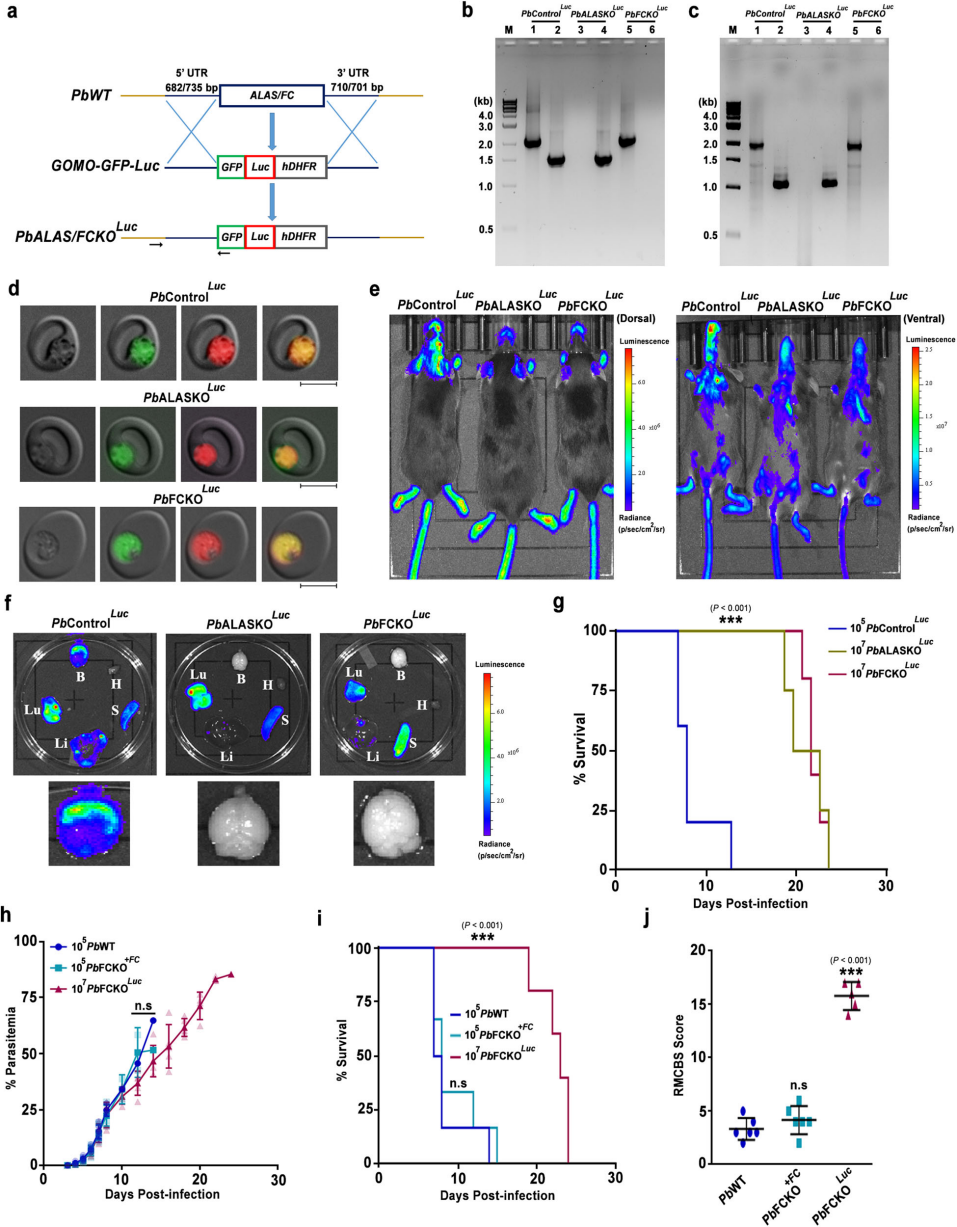
Generation of Luc-expressing heme pathway KO parasites, in vivo bioluminescence imaging of infected mice and genetic complementation. **a** Double-crossover recombination strategy to generate Luc-expressing *Pb*ALASKO (*Pb*ALASKO^*Luc*^) and *Pb*FCKO (*Pb*FCKO^*Luc*^) parasites. Black arrows represent the position of primers used for the confirmation of site-specific integration in *Pb*KO^*Luc*^ parasites (data provided in Supplementary Fig. 1). **b** Genomic DNA PCR confirmation for *ALAS* and *FC* deletions in *Pb*ALASKO^*Luc*^ and *Pb*FCKO^*Luc*^ parasites, respectively. Lane M: 1 kb ladder; Lane 1, 3 and 5: *ALAS* product (2.11 kb); Lane 2, 4 and 6: *FC* product (1.54 kb). *n* = 3 independent experiments. **c** RT-PCR confirmation for *ALAS* and *FC* deletions. Lane M: 1 kb ladder; Lane 1, 3 and 5: *ALAS* product (1.92 kb); Lane 2, 4 and 6: *FC* product (1.05 kb). *n* = 3 independent experiments. **d** Live GFP and m-cherry fluorescence of *Pb*Control^*Luc*^ and *Pb*KO^*Luc*^ parasites. Images were captured using 100x objective. Scale bar = 5 μm. *n* = 4 independent experiments. **e** Whole body bioluminescence imaging of *Pb*Control^*Luc*^ and *Pb*KO^*Luc*^ parasite-infected mice on day 8 post-infection. *n* = 3 independent experiments. **f** Ex vivo bioluminescence imaging of liver (Li), lungs (Lu), brain (B), heart (H) and spleen (S) of *Pb*Control^*Luc*^- and *Pb*KO^*Luc*^-infected mice. Enlarged images of brain are shown. **g** Mortality curves of mice infected with *Pb*Control^*Luc*^ (*n* = 5), *Pb*ALASKO^*Luc*^ (*n* = 4) and *Pb*FCKO^*Luc*^ (*n* = 5) parasites (***P < 0.001, log-rank (Mantel–Cox) test). **h** Growth analysis of *Pb*WT (*n* = 6), *Pb*FCKO^*+FC*^ (*n* = 6) and *Pb*FCKO^*Luc*^ (*n* = 5) parasites in C57BL/6 female mice. The data represent two different batches (mean ± SD; n.s not significant, Two-way ANOVA). Individual data points are shown with the respective light shaded colors. **i** Mortality curves of mice infected with *Pb*WT (*n* = 6), *Pb*FCKO^*+FC*^ (*n* = 6) and *Pb*FCKO^*Luc*^ (*n* = 5) parasites (n.s not significant, ***P < 0.001, log-rank (Mantel–Cox) test). **j** RMCBS score for mice infected with *Pb*WT (*n* = 6), *Pb*FCKO^*+FC*^ (*n* = 6) and *Pb*FCKO^*Luc*^ (*n* = 5) parasites on day 7/8 post-infection. *Pb*WT and *Pb*FCKO^*+FC*^ data represent the mice succumbed to ECM (mean ± SD; n.s - not significant, ***P < 0.001, unpaired *t*-test; two-sided). Source data are provided as a Source Data file.

These results were verified with another set of independent *Pb*KO^*Luc*^ parasite lines wherein, *ALAS* and *FC* genes were replaced individually with GFP-luciferase (Luc)-expressing cassette containing m-cherry (Fig. 2a). The successful replacement of *ALAS* and *FC* was confirmed by PCR analyses performed with DNA and RNA isolated from the respective *Pb*KO^*Luc*^ parasites (Fig. 2b, c), and by examining GFP and m-cherry fluorescence (Fig. 2d). For control, *c-ssurRNA* locus in the WT parasite was replaced with GFP-Luc-expressing cassette containing m-cherry (*Pb*Control^*Luc*^). The site-specific integrations in *Pb*Control^*Luc*^ and *Pb*KO^*Luc*^ parasites were also confirmed by PCR analyses (Supplementary Fig. 1c–e). In vivo bioluminescence studies showed accumulation of pRBCs in the brain of *Pb*Control^*Luc*^-infected mice, but not in the *Pb*KO^*Luc*^-infected mice (Fig. 2e) and this was confirmed by ex vivo imaging as well (Fig. 2f). The mice infected with *Pb*KO^*Luc*^ parasites were protected from ECM (Fig. 2g). To rule out any off-target effect, we performed genetic complementation in *Pb*FCKO^*Luc*^ by reintroducing *FC* (*Pb*FCKO^*+FC*^) expressed under its native promoter through stable integration (Supplementary Fig. 1f). The successful complementation of FC was verified by PCR and Western analyses (Supplementary Fig. 1g–i). *Pb*FCKO^*+FC*^ parasites behaved like WT parasites and caused ECM in the infected mice (Fig. 2h–j). These data suggested that the mice infected with heme pathway KO parasites are protected from ECM.

### Absence of ECM pathology in heme pathway KO parasite-infected mice

**Fig. 3:**
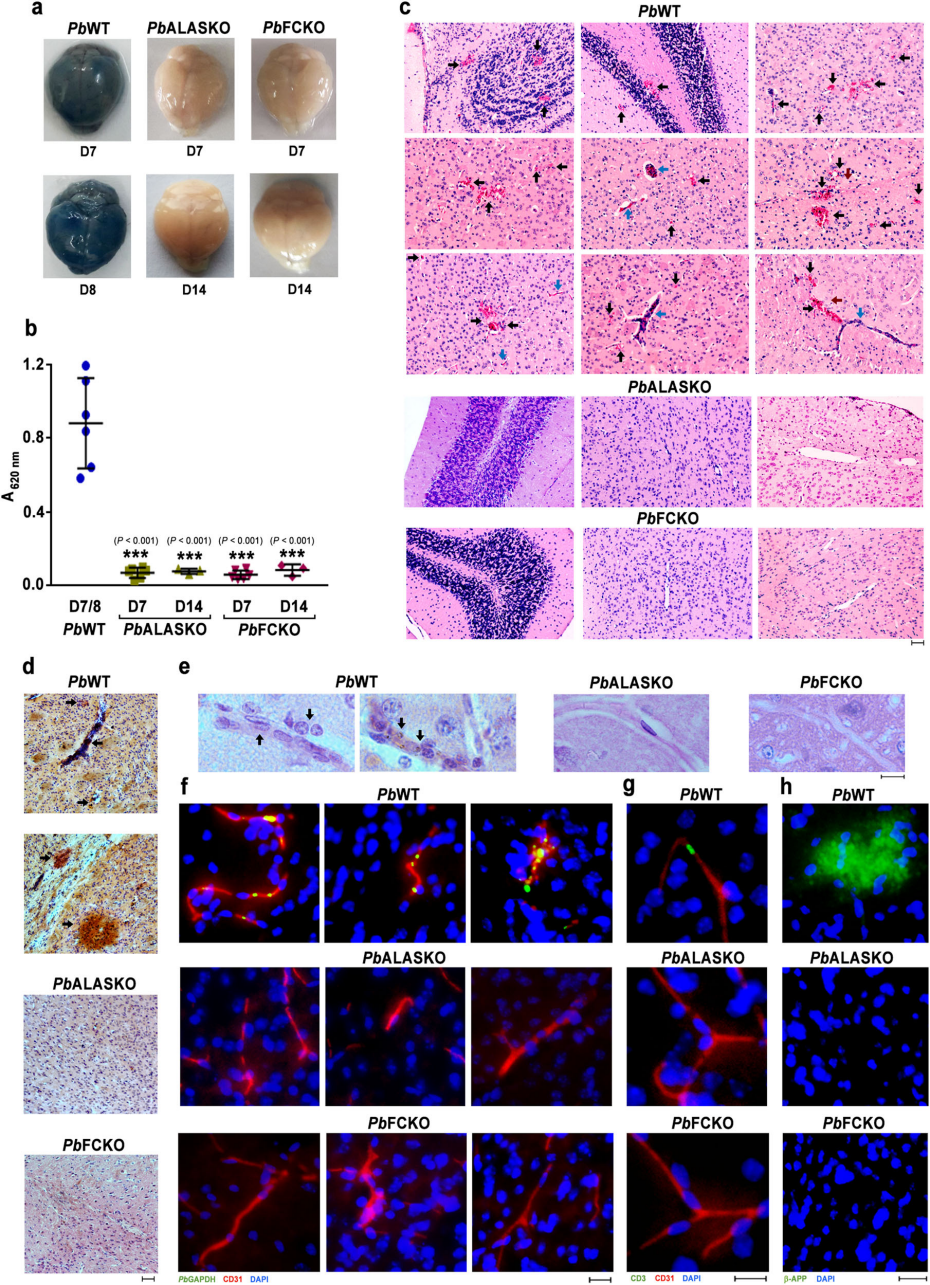
Assessment of cerebral pathology in heme pathway KO parasite-infected mice. **a** Evans blue extravasation in the brain of mice infected with *Pb*WT and heme pathway *Pb*KO parasites. **b** Quantification of Evans blue in the brain samples of mice infected with *Pb*WT (*n* = 6) and heme pathway *Pb*KO (*n* = 9) parasites. (mean ± SD; ***P < 0.001, unpaired *t*-test; two-sided). **c** H&E staining of the brain sections prepared from *Pb*WT and *Pb*KO parasite-infected mice. Black arrows—intracerebral and petechial hemorrhages, blue arrows—thrombosed blood vessels and brown arrows—gross demyelination. Images were captured using 10x objective. Scale bar = 50 μm. *n* = 3–5 independent experiments. **d** IgG extravasation in the brain sections of *Pb*WT and *Pb*KO parasite-infected mice. Black arrows—areas showing IgG immunoreactivity. Images were captured using 10x objective. Scale bar = 50 μm. *n* = 3 independent experiments. **e** H&E staining of the brain sections indicating (black arrows) occluded vasculatures containing luminal and abluminal leukocytes, and parasite-derived Hz. Images were captured using 60x objective. Scale bar = 10 μm. *n* = 3 independent experiments. **f** Immunofluorescence analysis of parasite accumulation in the brain sections of *Pb*WT and *Pb*KO parasite-infected mice. *n* = 3 independent experiments. **g** Immunofluorescence analysis of CD3^+^ cells in the blood vessels of *Pb*WT and *Pb*KO parasite-infected mice. *n* = 2 independent experiments**. h** Immunofluorescence analysis of β-APP staining in the brain sections of *Pb*WT and *Pb*KO parasite-infected mice. *n* = 2 independent experiments. **f**–**h** Images were captured using 20x objective. Scale bar = 20 μm. Source data are provided as a Source Data file.

To evaluate the integrity of BBB and assess vascular leakage, Evans blue extravasation analyses were carried out. While the brain collected from WT-infected mice on day 7/8 stained intensely with Evans blue, the extravasation of Evans blue into the brain was barely detectable in KO-infected mice on day 7 and 14 (Fig. 3a). Quantification of Evans blue in the brain extracts confirmed this observation (Fig. 3b). Histopathological assessment of hematoxylin and eosin (H&E)-stained brain sections of WT-infected mice on day 7 showed intracerebral hemorrhages with extravasation of erythrocytes into the perivascular space, petechial hemorrhages, thrombosed and leukocyte-packed vessels, gross demyelination and myelin pallor. No such hallmark features of ECM could be detected in the brain sections of KO-infected mice (Fig. 3c). Immunohistochemical studies performed with the brain sections of WT-infected mice showed the extravasation of IgG in cerebral parenchyma and the presence of IgG in occluded vessels and hemorrhages, but not in the KO-infected mice (Fig. 3d). Luminal and abluminal leukocytes, and parasite-derived Hz could be detected in the occluded vasculature of WT-infected mice. (Fig. 3e). Immunofluorescence analyses of the brain sections using *Pb* glyceraldehyde-3-phosphate dehydrogenase (GAPDH) and mouse CD31 antibodies showed the accumulation of parasites in CD31^+^ vasculature and extravascular parasites in the hemorrhages of WT-infected mice, but not in the KO-infected mice (Fig. 3f). Similarly, antibodies specific for CD3 and β-amyloid precursor protein (β-APP) showed the accumulation of CD3^+^ T cells in the cerebral vasculature (Fig. 3g) and axonal injury in the brain sections of WT-infected mice, but not in the KO-infected mice (Fig. 3h). These data suggested the absence of ECM-associated brain lesions in mice infected with heme pathway KO parasites.

### Inflammatory parameters in heme pathway KO parasite-infected mice

**Fig. 4:**
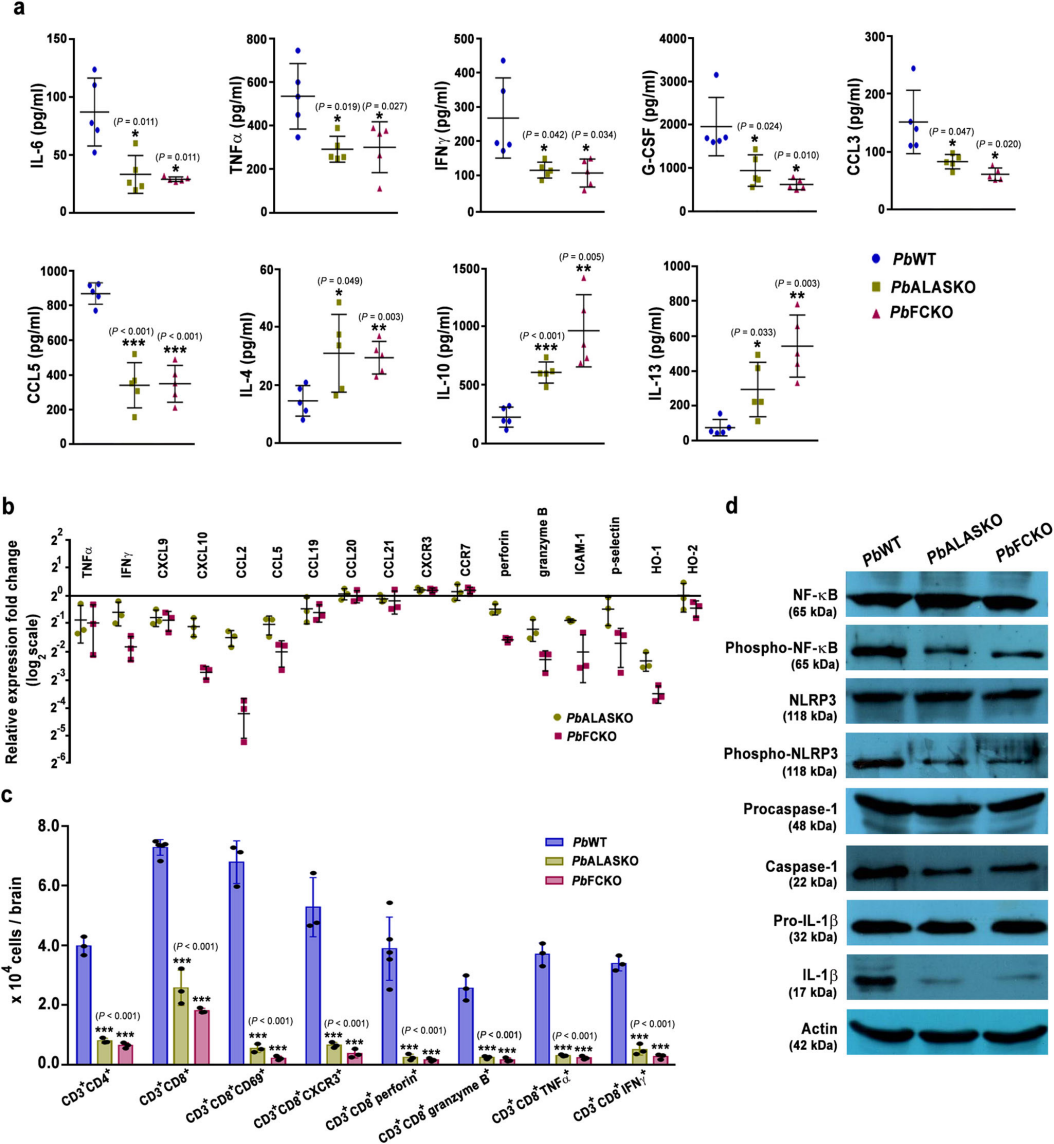
Assessment of inflammatory parameters in heme pathway KO parasite-infected mice. **a** Plasma cytokine and chemokine levels of *Pb*WT- and *Pb*KO-infected mice (*n* = 5) (mean ± SD; *P < 0.05, **P < 0.01, ***P < 0.001, unpaired *t*-test; two-sided). **b** qPCR analyses of host transcripts in the brain samples of infected mice. Expression levels were normalized with mouse GAPDH. Relative expression fold changes of mRNA transcripts in the KO-infected mice with respect to WT-infected mice (mean ± SD) are shown (*n* = 3). **c** Flow cytometry analyses of T cells in the brain samples of infected mice. Mice on day 7/8 post-infection were used and the data for each cell type were obtained from at least three different mice infected with *Pb*WT or *Pb*KO parasites (mean ± SD; ***P < 0.001, Two-way ANOVA). Individual data points are shown as black circles. Gating strategy and representative flow cytometry plots are shown in the Supplementary Fig. 2. **d** Western analyses of brain homogenates prepared from *Pb*WT- and *Pb*KO-infected mice. 200 μg of total protein was used from the pooled brain homogenates of three different mice for *Pb*WT, *Pb*ALASKO and *Pb*FCKO. *n* = 2 independent experiments. Full-length blots are provided in the Supplementary Fig. 7. Source data are provided as a Source Data file.

Multiplex assays performed for mice infected with KO parasites on day 8 showed a significant decrease in the plasma levels of proinflammatory cytokines and chemokines—IL-6, TNFα, IFNγ, G-CSF, CCL3 (MIP-1α) and CCL5 (RANTES). There was also a significant increase in anti-inflammatory cytokines—IL-4, IL-10 and IL-13 (Fig. 4a). Quantitative RT-PCR analyses examining the expression of cytokines, chemokines, chemokine receptors and other key mediators of cerebral pathogenesis in the brain samples of KO-infected mice showed a substantial reduction of >1.5 fold in the transcript levels of TNFα, IFNγ, CXCL9, CXCL10, CCL2 (MCP-1), CCL5, CCL19, perforin, granzyme B, ICAM-1, p-selectin and HO-1. In particular, the decrease in IFNγ, CXCL10, CCL2, CCL5, granzyme B, ICAM-1 and HO-1 in FCKO was greater than 3-fold (Fig. 4b). Flow cytometry analyses of the leukocytes isolated from the brain samples of KO-infected mice showed significant reduction in CD3^+^ CD4^+^ and CD3^+^ CD8^+^ double positive T cells, and CD3^+^ CD8^+^ CD69^+^, CD3^+^ CD8^+^ CXCR3^+^, CD3^+^ CD8^+^ perforin^+^, CD3^+^ CD8^+^ granzyme B^+^, CD3^+^ CD8^+^ TNFα^+^ and CD3^+^ CD8^+^ IFNγ^+^ triple positive T cells (Fig. 4c and Supplementary Fig. 2). Western analyses for the brain homogenates of KO-infected mice showed reduction in phospho-NLRP3, phospho-NF-κB, cleaved caspase-1 and IL-1β (Fig. 4d). RNA levels of schizont membrane-associated cytoadherence protein (SMAC), skeleton-binding protein 1 (SBP1) and membrane-associated histidine-rich protein-1a (MAHRP1a) mediating sequestration and cytoadherence in *Pb* were comparable between WT and FCKO parasites (Supplementary Fig. 3a). Further, there was no significant difference between the in vitro cytoadherence of *Pb*Control^*Luc*^- and *Pb*FCKO^*Luc*^-pRBCs to TNFα-prestimulated mouse brain endothelial cells (Supplementary Fig. 3b, c). These data suggested that although the parasite machinery underlying cytoadherence is retained, mice infected with heme pathway KO parasites show reduced in vivo sequestration due to an overall decrease in systemic and neuronal inflammation and T cell infiltration in the brain milieu.

### Decreased Hz formation in heme pathway KO parasites

**Fig. 5:**
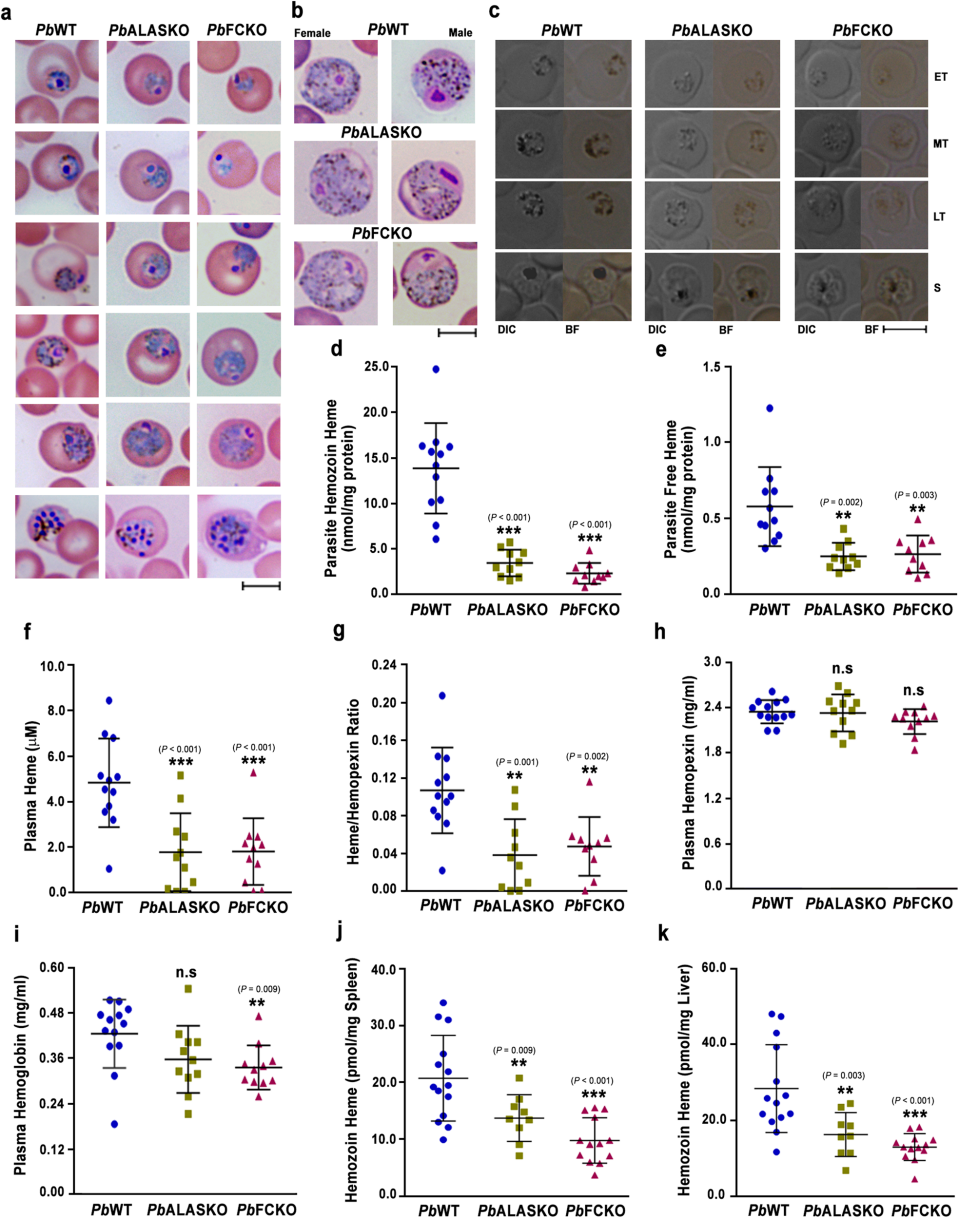
Hz and heme levels in heme pathway KO parasites. **a**, **b** Bright field images of Giemsa-stained *Pb*WT, *Pb*ALASKO and *Pb*FCKO asexual stage parasites and gametocytes, respectively, showing Hz content. Images were captured using 100x objective. *n* = 3–5 independent experiments. Scale bar = 5 μm. **c** Hz content in differential interference contrast (DIC; left) and bright field images (right) of paraformaldehyde-fixed pRBCs containing *Pb*WT and *Pb*KO parasites. Images were captured using 100x objective. Scale bar = 5 μm. *n* = 3 independent experiments. **d** Hz levels in *Pb*WT (*n* = 12) and *Pb*KO (*n* = 10) parasites. **e** Free heme levels in *Pb*WT (*n* = 11) and *Pb*KO (*n* = 10) parasites. **f** Free heme levels in the plasma samples of *Pb*WT (*n* = 12) and *Pb*KO (*n* = 11) parasite-infected mice. **g** Heme/Hemopexin ratio in the plasma samples of *Pb*WT (*n* = 12) and *Pb*KO (*n* = 10) parasite-infected mice. **h** Plasma hemopexin levels of *Pb*WT (*n* = 13) and *Pb*KO (*n* = 11) parasite-infected mice. **i** Plasma hemoglobin levels of *Pb*WT (*n* = 13) and *Pb*KO (*n* = 11) parasite-infected mice. **j**, **k** Hz load in the spleen and liver of *Pb*WT (*n* = 14) and *Pb*KO (*n* = 9 for ALASKO; *n* = 13 for FCKO) parasite-infected mice, respectively. **d**–**k** The data represent mean ± SD (n.s not significant, **P < 0.01, ***P < 0.001, unpaired *t*-test; two-sided). Source data are provided as a Source Data file.

A substantial decrease in Hz formation was observed in the Giemsa-stained peripheral blood smears of KO parasites. This was more prominent in the asexual stages (Fig. 5a) than gametocytes (Fig. 5b), and could be readily detected in almost 60–70% of the pRBCs containing asexual stages. These findings were verified by examining Hz content in paraformaldehyde-fixed pRBCs (Fig. 5c) and Hz dynamics in live pRBCs (Supplementary Movie 1, Supplementary Movie 2 and Supplementary Movie 3). To confirm, total Hz content of the KO parasites normalized with respect to the protein was examined and it was 20–25% of the WT (Fig. 5d). During Hz formation in the FV, heme can leach into the parasite cytosol. Free heme in the parasite lysates of the KO parasites prepared by hypotonic lysis showed around 55% decrease when compared with WT (Fig. 5e). In addition, there was a significant decrease of around 55–60% in the plasma free heme and heme/hemopexin ratio of the KO-infected mice (Fig. 5f, g). However, there were no significant differences between the plasma hemopexin levels of WT- and KO-infected mice (Fig. 5h), and there was 15–20% decrease in the plasma Hb levels that was statistically significant in case of FCKO, but not in ALASKO (Fig. 5i). The quantification of Hz load in the spleen and liver showed 40–50% decrease in the KO-infected mice (Fig. 5j, k). A similar decrease in the Hz content and free heme was observed for the KO parasites isolated from Balb/c mice (Supplementary Fig. 3d, e). These results suggested an overall decrease in the Hz synthesis of KO parasites. This was further verified by the restoration of Hz and free heme levels in *Pb*FCKO^*+FC*^ parasites isolated from C57BL/6 mice (Supplementary Fig. 3 f–h) and the presence of cerebral pathogenesis in the *Pb*FCKO^*+FC*^-infected mice (Supplementary Fig. 3i–k).

**Fig. 6:**
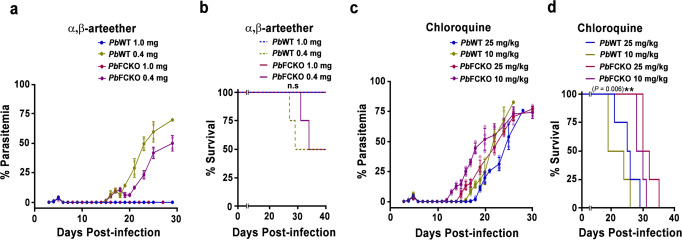
Sensitivity of WT and FCKO parasites to α, β-arteether and chloroquine. **a**, **b** Blood parasitemia and mortality curves of infected mice treated with α,β-arteether, respectively. The data represent four mice for each group (n.s not significant, log-rank (Mantel–Cox) test). **c**, **d** Blood parasitemia and mortality curves of infected mice treated with chloroquine, respectively. The data represent four mice for each group (**P < 0.01, log-rank (Mantel–Cox) test). For (**a**, **c**), the data represent mean ± SD. Individual data points are shown with the respective light shaded colors. Source data are provided as a Source Data file.

Heme and Hz are associated with the function of antimalarial drugs—artemisinin and chloroquine^44,45^. Therefore, we were interested in examining the effect of α,β-arteether (artemisinin derivative) and chloroquine on FCKO-infected C57BL/6 mice in vivo. For α,β-arteether treatment, a single intramuscular dose of 1 mg/mouse or 0.4 mg/mouse was administered on day 5 post-infection when the blood parasitemia was around 5%. For chloroquine treatment, a daily dose of 25 mg/kg or 10 mg/kg was administered intraperitoneally for five consecutive days from day 5 post-infection. While 1 mg of α,β-arteether could clear the WT and FCKO parasites, 50% recrudescence was observed for 0.4 mg dosage in the WT- and FCKO-infected mice. The day of recrudescence was comparable between WT and FCKO parasites and the parasites were detectable in peripheral smears prepared on day 12 post-infection. However, the growth of FCKO parasites in mice showing recrudescence was delayed in the subsequent days and this was reflected in the mortality (Fig. 6a, b). The assessment of parasite load at the time of recrudescence on day 12 using *Pb*GAPDH primers showed that *C*_*t*_ values normalized against mouse GAPDH for FCKO-infected mice were ~1.5 cycles more than WT-infected mice (Supplementary Fig. 4a–d), suggesting 3-fold less parasite load in FCKO-infected mice. In case of chloroquine, recrudescence was observed for WT and FCKO parasites with both the doses that were tested and importantly, FCKO parasites appeared 2–4 days earlier than WT (Fig. 6c, d). This was also confirmed by performing qPCR analysis on day 13 for 25 mg/kg and day 10 for 10 mg/kg doses. In both the cases, *C*_*t*_ values obtained for the parasite load in FCKO-infected mice were ~4 cycles lower than WT-infected mice, suggesting 16-fold higher parasite load in FCKO-infected mice (Supplementary Fig. 4e–h). Further, one out of four and two out of four WT-infected mice died of CM in 25 mg/kg and 10 mg/kg treatment, respectively. Despite showing an early recrudescence, all the FCKO-infected mice died of anemia and their mortality was delayed in comparison to WT-infected mice (Fig. 6d). These results suggested that chloroquine sensitivity is compromised in FCKO parasites as observed by the earlier recrudescence, while α,β-arteether sensitivity remains unaltered in vivo in mice.

### De novo heme is essential for the functional integrity of FV

**Fig. 7:**
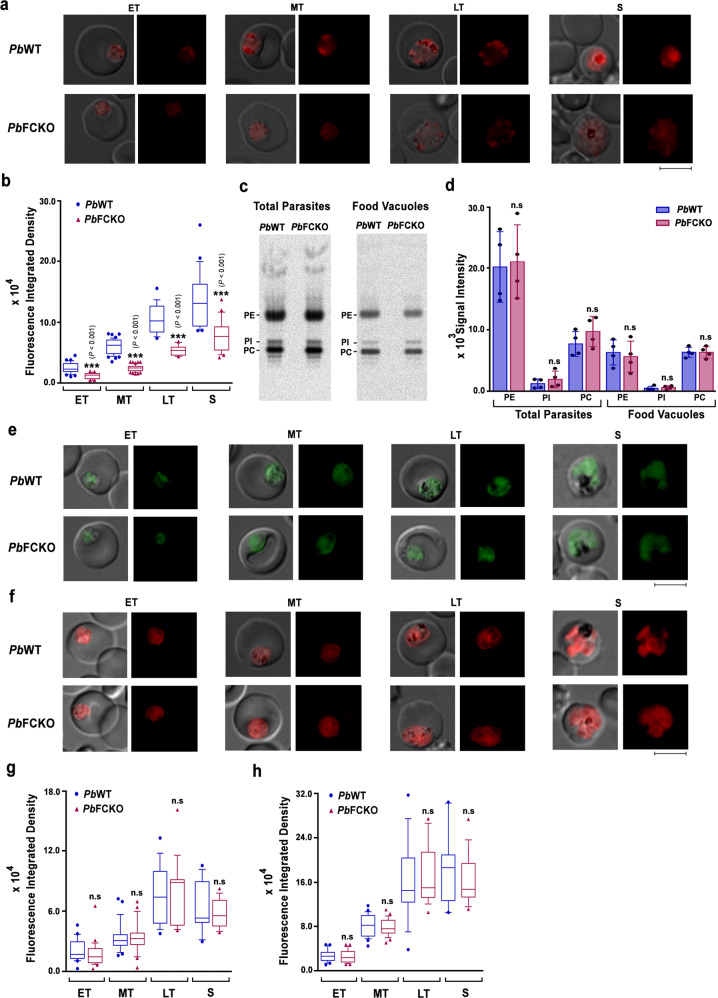
Assessment of food vacuole pH, phospholipids and neutral lipids in FCKO parasites. **a** Live cell fluorescence imaging of LysoTracker Deep Red uptake in *Pb*WT and *Pb*FCKO parasites. **b** Quantification of fluorescence signal from various stages. *Pb*WT − 98 trophozoites and 22 schizonts; *Pb*FCKO − 95 trophozoites and 23 schizonts. ET, MT and LT early, mid and late trophozoites; S schizonts. Images were captured using 100x objective. Scale bar = 5 μm. (***P < 0.001, unpaired *t*-test; two-sided). **c** TLC separation of ^32^P-orthophosphoric acid radiolabelled phospholipids for *Pb*WT and *Pb*FCKO total parasites and FVs. Half the phospholipid preparation was used for total parasites. For FVs, entire preparation was used. PC phosphatidylcholine, PI phosphatidylinositol, PE phosphatidylethanolamine. **d** Band intensities quantified (mean ± SD; n.s not significant, unpaired *t*-test; two-sided). The data represent four different experiments. Individual data points are shown as black circles. **e** Live cell fluorescence imaging of BODIPY 493/503 staining in *Pb*WT and *Pb*FCKO parasites. Images were captured using 100x objective. Scale bar = 5 μm. **f** Live cell fluorescence imaging of Nile Red staining in *Pb*WT and *Pb*FCKO parasites. Images were captured using 100x objective. Scale bar = 5 μm. **g** Quantification of the fluorescence signal from various stages for BODIPY 493/503 staining. The data represent 69 trophozoites and 15 schizonts for *Pb*WT, and 68 trophozoites and 14 schizonts for *Pb*FCKO (n.s not significant, unpaired *t*-test; two-sided). **h** Quantification of the fluorescence signal from various stages for Nile Red staining. The data represent 68 trophozoites and 14 schizonts for *Pb*WT, and 70 trophozoites and 14 schizonts for *Pb*FCKO (n.s not significant, unpaired *t*-test; two-sided). ET early trophozoites, MT mid trophozoites, LT late trophozoites, S schizonts. For (**b**, **g**, **h**), Box and whisker plots display 10th and 90th percentile as the whiskers, 25th−75th percentile as the boxes and median as the centre line. Source data are provided as a Source Data file.

**Fig. 8:**
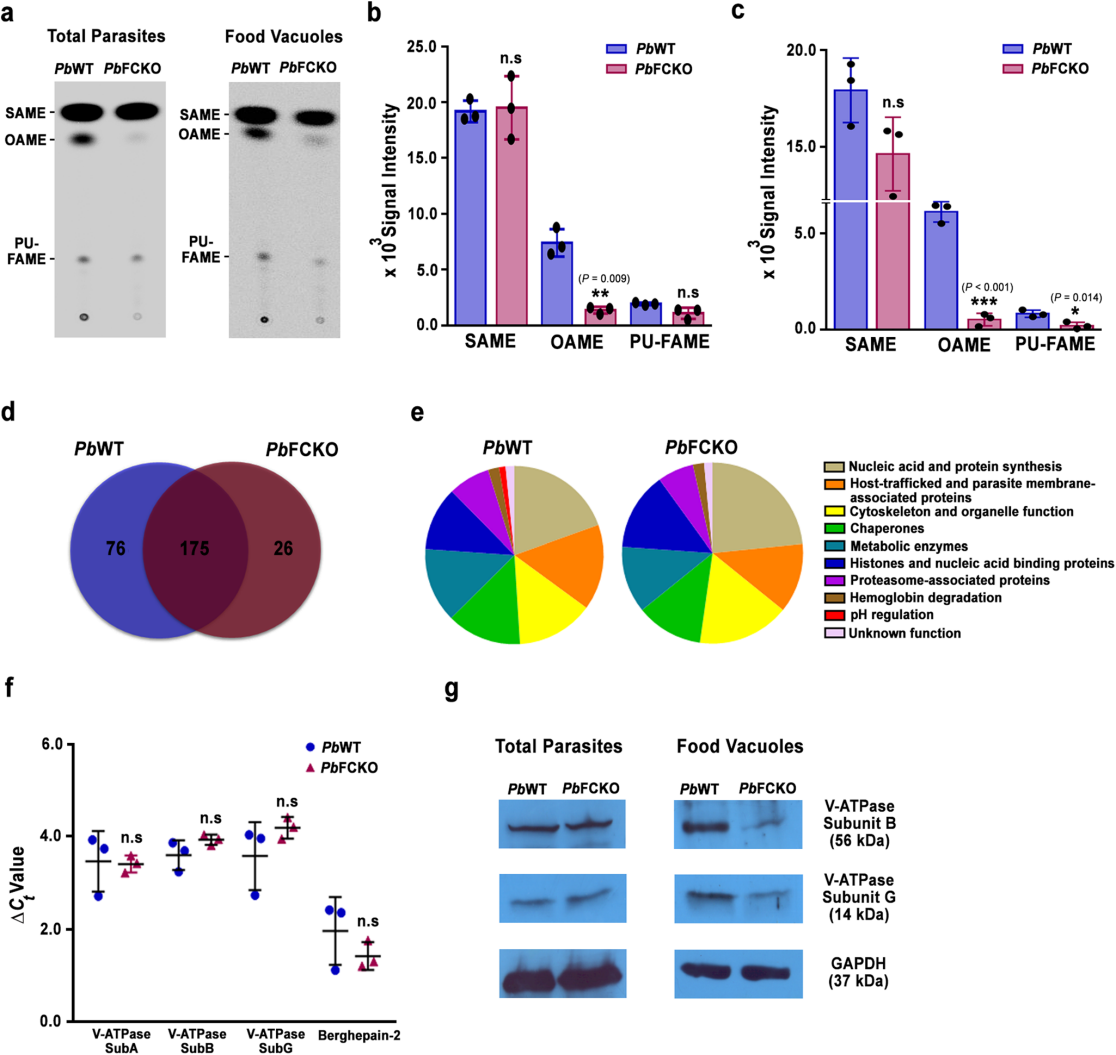
Evaluation of OA synthesis and FV proteomics for WT and FCKO parasites. **a** TLC separation of ^14^C-SA radiolabelled unsaturated FAMEs for *Pb*WT and *Pb*FCKO total parasites and FVs. One third of the FAME preparation used for total parasites and entire preparation used for FVs. **b**, **c** Band intensities quantified for total parasites and FVs, respectively (mean ± SD; n.s not significant, *P < 0.05, **P < 0.01, ***P < 0.001, unpaired *t*-test; two-sided), from three different experiments. Individual data points are shown as black circles. **d** Venn diagram of total proteins identified in the *Pb*WT and *Pb*FCKO FVs. **e** Functional classification of the proteins based on gene ontologies available at PlasmoDB and UniProt databases. Supplementary Data 1 and Supplementary Data 2 have the complete set of details related to *Pb*WT and *Pb*FCKO FV proteome analyses. Three different FV preparations of *Pb*WT and *Pb*FCKO were pooled independently to get adequate amount of protein for the proteomics analyses. **f** qPCR analysis of RNA transcripts. The Δ*C*_*t*_ values obtained with respect to parasite GAPDH were plotted (mean ± SD; n.s not significant, unpaired *t*-test; two-sided). The data represent three different preparations of RNA from WT and FCKO parasites. **g** Western analysis of V-type H^+^ATPase subunits. The total parasite preparations represent parasite pellets pooled from two different mice. For FVs, three different FV pellets were pooled separately for WT and FCKO. These preparations were independent of those that were used for proteomics analysis. GAPDH is known to be present in FVs and therefore, used as a control for total parasite and FV preparations. (*n* = 2 independent experiments). Full-length blots are provided in the Supplementary Fig. 7. Source data are provided as a Source Data file.

To understand the molecular mechanisms underlying decreased Hz synthesis, we assessed pH, lipids and protein content of the FVs in FCKO parasites and compared with WT parasites. Live fluorescence imaging of pRBCs incubated with acidophilic dye LysoTracker Deep Red indicated less fluorescence in the FCKO parasites (Fig. 7a), suggesting that the pH of FCKO FV is compromised. The quantification of fluorescence signal intensities showed ~50% decrease (Fig. 7b). We examined the phospholipid content and could not find significant differences for in vitro ^32^P-orthophosphoric acid radiolabelling of major phospholipids - phosphatidylcholine (PC), phosphatidylinositol (PI) and phosphatidylethanolamine (PE), in the total parasites and FVs of FCKO parasites (Fig. 7c, d and Supplementary Fig. 5a). Similarly, the assessment of neutral lipid content by live fluorescence imaging of pRBCs stained with BODIPY 493/503 and Nile Red did not show significant differences (Fig. 7e–h). Lipids associated with parasite Hz are reported to be abundant in monohydroxy derivatives of polyenoic fatty acids, and unsaturated glycerophospholipids induce rapid and efficient Hz formation in hematophagous insect *Rhodnius prolixus*^46,47^. GC-MS analysis of fatty acid methyl esters (FAMEs) prepared from the FVs of *Pb* parasites indicated the presence of oleic acid (OA) as a major unsaturated fatty acid followed by arachidonic acid along with other saturated fatty acids, fatty acyl alcohols and derivatives, alkanes, etc. (Supplementary Fig. 5b and Supplementary Table 1). Malaria parasite scavenges stearic acid (SA) from plasma and converts it into OA by ER-localized Δ9-desaturase (stearoyl-CoA 9-desaturase). The *cis* double bond formation in OA requires heme since it utilizes the electrons transferred by cytochrome b5 from NADH cytochrome b5 reductase^48,49^. We performed in vitro ^14^C-SA radiolabelling to assess OA synthesized by the parasite. The separation of unsaturated FAMEs prepared from FCKO total parasites and FVs showed almost 80–90% decrease in the radiolabelling of OA methyl ester (OAME) in comparison to WT (Fig. 8a–c), with no significant differences in the signal intensities of SA methyl ester (SAME). The identity of the slower migrating polyunsaturated FAME (PU-FAME) that also showed a significant decrease in FVs is yet to be established and there are no clear-cut evidences for the presence of other desaturases in the parasite. There was a significant ~50% decrease in the ^14^C-SA radiolabelling of FCKO FVs, but not in the total parasites (Supplementary Fig. 5c).

FV proteomics is challenging due to the presence of large amounts of host Hb, Hb degradation peptides and indigenous proteases. To the best of our knowledge, there is only one report on *Pf* FV proteomics based on LC-MS/MS analysis of proteins excised from SDS-PAGE gel that identified 116 proteins excluding elongation factors and ribosomal proteins^50^. Our repeated attempts to perform differential proteomics for WT and FCKO FVs by Isobaric tags for relative and absolute quantitation (iTRAQ) were unsuccessful because of the interference from host Hb. LC-MS/MS of in-solution trypsin digested FV protein extracts solubilized with 6 M urea was successful. Around 68% of the proteins identified earlier in the total *Pf* FV proteome^50^ were present in our FV preparations from *Pb*. The quality of FV preparations could be assessed by the presence of signature FV proteins such as plasmepsin IV, berghepain, aminopeptidases, subunits of vacuolar-type H^+^ATPase (V-type H^+^ ATPase), together with parasitophorous vacuolar (PV) proteins including exported protein 1 (Exp1), Exp2, early transcribed membrane protein, PV1, PV5 (lipocalin) etc., and Rab GTPases associated with cytostome-FV trafficking. A total number of 251 and 201 proteins could be identified for WT and FCKO FVs, respectively, and 175 proteins were common between them suggesting an overall consistency in the preparations (Fig. 8d). The peptide intensities and spectral counts were comparable between WT and FCKO FV preparations (Supplementary Fig. 6a, b). In agreement with the decreased uptake of LysoTracker Deep Red in FCKO pRBCs, none of the subunits of V-type H^+^ATPase—a proton pump maintaining the acidic pH of FV^51^ could be detected in FCKO FVs suggesting the lower abundance of these proteins. In WT FV preparations, A, B and G subunits of V-type H^+^ATPase could be detected (Fig. 8e, Supplementary Data 1 and Supplementary Data 2). While plasmepsin IV, the only *Pb* aspartic protease involved in Hb degradation could be detected in WT and FCKO FVs, berghepain-2—a cysteine protease involved in Hb degradation^52,53^ could not be detected in FCKO FVs (Supplementary Data 1 and Supplementary Data 2). qPCR analysis indicated that the RNA levels of these proteins are comparable between WT and FCKO parasites (Fig. 8f). However, Western analysis carried out for V-type H^+^ATPase subunits B and G showed a significant decrease in FCKO FVs with not much change in the total parasites (Fig. 8g). The rest of the proteins unique for WT and FCKO FVs include proteasome subunits, ribosomal proteins, metabolic enzymes etc., (Supplementary Data 1 and Supplementary Data 2) that are not related to Hz formation and known to be present in FV preparations^50^. Further, in agreement with less Hz formation, FVs of FCKO parasites appeared paler in comparison to WT (Supplementary Fig. 6c). While iTRAQ experiments were unsuccessful in identifying the parasite FV proteins because of the interference from host Hb, they could be used to assess the host Hb accumulation in FCKO FVs. There was ~3–4 -fold increase in Hb α and β chains in FCKO FVs as quantified by iTRAQ (Supplementary Fig. 6d; Supplementary Data 3). Similar to the mutants of Hb proteolysis^52^, translucent vesicles were also observed in the Giemsa-stained smears of FCKO parasites (Supplementary Fig. 6e). All these evidences suggested that the functional integrity of FCKO FVs is compromised in terms of pH, lipid unsaturation and proteins that in turn can collectively lead to a decreased Hz formation.

### Griseofulvin treatment protects mice from ECM

**Fig. 9:**
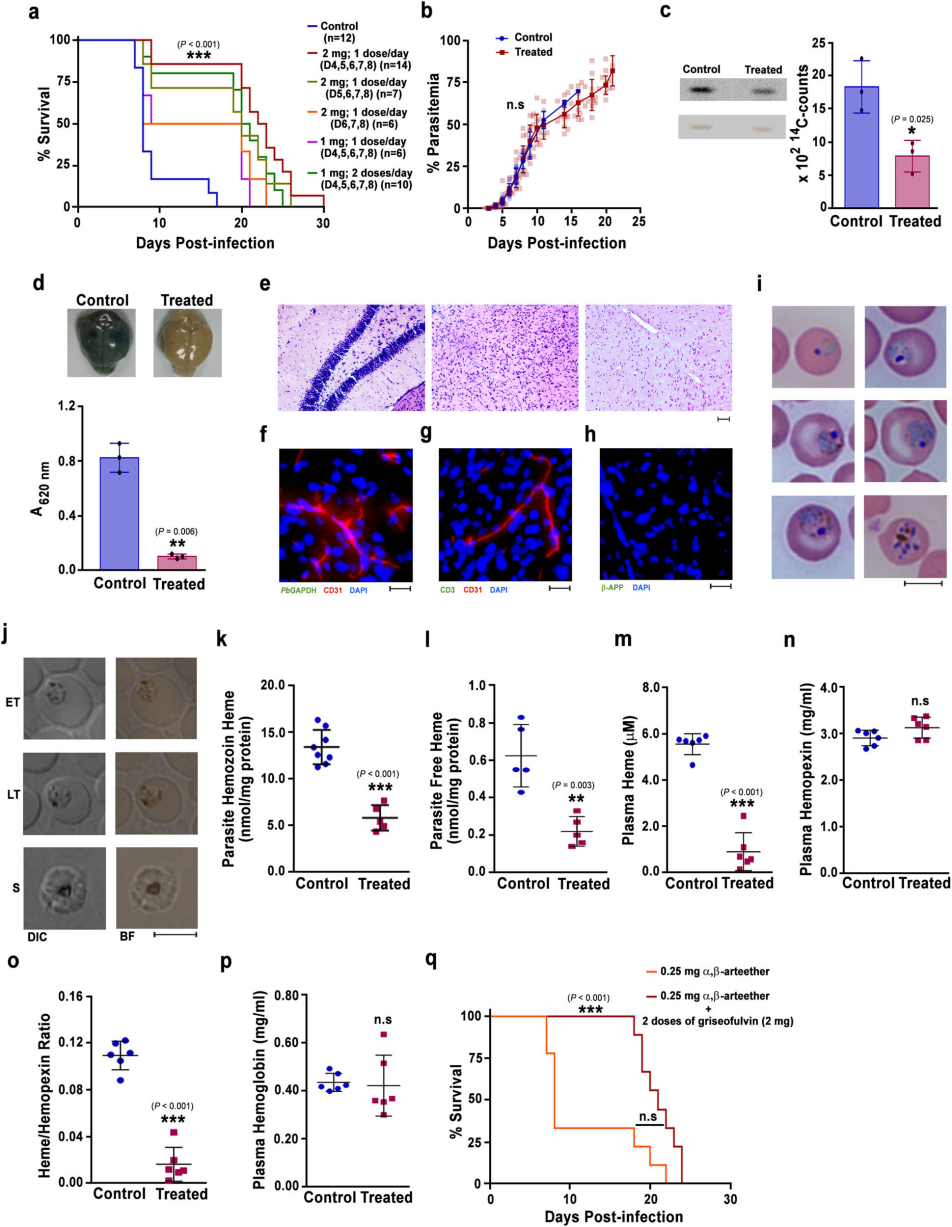
Effect of griseofulvin treatment on CM pathogenesis. **a** Mortality curves of *Pb*WT-infected mice treated with different dosages of griseofulvin (***P < 0.001, log-rank (Mantel–Cox) test). **b** Growth curve analysis (*n* = 12) for 2 mg dose per day on day 4, 5, 6, 7, and 8 (mean ± SD; n.s not significant, Two-way ANOVA). **c** Phosphorimager and scanned images of TLC performed for ^14^C-ALA labeled parasite free heme and the radioactive counts measured for three different experiments (mean ± SD; n.s not significant, *P < 0.05, unpaired *t*-test; two-sided). Individual data points are shown as black circles. Full-length scans are provided in the Supplementary Fig. 7. **d** Extravasation of Evans blue and its quantification (*n* = 3) (mean ± SD; **P < 0.01, unpaired *t*-test; two-sided). Individual data points are shown as black circles. **e** H&E staining of the brain sections. Images were captured using 10x objective. Scale bar = 50 μm. *n* = 3 independent experiments. **f** Parasite accumulation **g** CD3^+^ cells in the blood vessels. *n* = 2 independent experiments. **h** Axonal injury in the brain sections. Images were captured using 20x objective. Scale bar = 20 μm. *n* = 3 independent experiments. **i** Giemsa-stained parasites. Images were captured using 100x objective. Scale bar = 5 μm. *n* = 3 independent experiments. **j** Hz content in differential interference contrast (DIC; left) and bright field images (right) of paraformaldehyde-fixed RBCs. Images were captured using 100x objective. Scale bar = 5 μm. *n* = 3 independent experiments. **k** Parasite Hz (*n* = 8 for control; *n* = 5 for treated). **l** Parasite free heme (*n* = 5). **m** Plasma free heme (*n* = 6). **n** Plasma hemopexin (*n* = 6). **o** Plasma heme/hemopexin ratio. **p** Plasma hemoglobin (*n* = 6). **k**–**p** (mean ± SD; n.s not significant, **P ≤ 0.01, ***P < 0.001, unpaired *t*-test; two-sided). **q** Mortality curves for *Pb*WT-infected C57BL/6 female mice treated with α,β-arteether alone (*n* = 9) and α,β-arteether in combination with griseofulvin (*n* = 9). The data represent two different batches. (***P < 0.001, n.s not significant, log-rank (Mantel–Cox) test). Source data are provided as a Source Data file.

Griseofulvin, isolated from *Penicillium griseofulvum*, is a FDA-approved antifungal drug used to cure tinea infections. It interacts with fungal microtubules and disrupts spindle assembly leading to mitotic arrest. In humans, griseofulvin dosage is given to the extent of 1000 mg/day in adults and 10 mg/kg/day in children for several weeks^54–56^. It can also inhibit FC by generating N-methyl protoporphyrin IX (NMPP) through the action of cytochrome P450 enzymes^57^. It has already been shown that the malaria parasite can convert griseofulvin into NMPP^58^. Therefore, we evaluated the potential of griseofulvin in preventing ECM by treating WT-infected C57BL/6 mice from day 4 when the blood parasitemia was around 2%. A single dose of 2 mg/day (comparable with the dosage of humans) administered from day 4 and continued until day 8 showed the best protection. While ~80% of the control mice succumbed to ECM within day 10, >80% of the treated mice were protected from ECM. Similar protection was also observed for the mice treated with 1 mg dose, twice a day from day 4 to day 8 (Fig. 9a). There was also a significant delay in the mortality that occurred due to anemia. While the mortality in ECM-escaped control mice occurred within day 17, >80% of the treated mice could survive beyond day 20 with almost 50% of them surviving even beyond day 24. Interestingly, the growth curves of WT parasites in treated and untreated mice were very much comparable (Fig. 9b). We analyzed heme synthesis in griseofulvin-treated WT parasites by incubating the in vivo-treated pRBCs in vitro with ^14^C-ALA (committed precursor of heme synthesis) for 9 h and there was around 60% decrease in the ^14^C-labeling of free heme (Fig. 9c). Further, griseofulvin-treated mice showed less Evans blue extravasation in the brain (Fig. 9d), with the absence of intracerebral hemorrhages (Fig. 9e), lack of accumulation of parasites and CD3^+^ T cells in the cerebral vasculature (Fig. 9f, g) and undetectable axonal injury (Fig. 9h). Giemsa-stained peripheral smears and paraformaldehyde-fixed pRBCs showed less Hz content (Fig. 9i, j). As observed for KO parasites, the total Hz content in the griseofulvin-treated parasites was around 50–60% less when compared with untreated parasites (Fig. 9k) and there was close to 60% decrease in the free heme levels (Fig. 9l). Further, the plasma levels of heme, hemopexin and Hb together with heme/hemopexin ratio of the griseofulvin-treated WT-infected mice were comparable with the KO-infected mice (Fig. 9m–p). These results suggested that griseofulvin can prevent ECM through inhibition of parasite heme synthesis.

Our next interest was to examine the potential of griseofulvin as an adjunct drug with α,β-arteether (primary artemisinin component of ACTs) in preventing ECM. The dose optimization studies performed with α,β-arteether suggested that a single intramuscular dose of 0.25 mg/mouse on day 6 when there was onset of neurological symptoms and blood parasitemia was around 10%, led to ECM in ~60–70% of WT-infected mice. ECM occurred despite the clearance of peripheral parasitemia as observed in the blood smears (Supplementary Fig. 6f, g). However, the inclusion of two doses of 2 mg griseofulvin at 24 h interval on day 6 and 7, post-α,β-arteether treatment, led to a complete protection of ECM (Fig. 9q). There were no significant differences in day 7 parasite load measured by qPCR analysis and in the mortality due to anemia caused by recrudescence for ECM-protected mice treated with the combination of α,β-arteether and griseofulvin in comparison with α,β-arteether alone (Supplementary Fig. 6h, Fig. 9q).

### Griseofulvin treatment inhibits Hz formation in *Pf* parasites

**Fig. 10:**
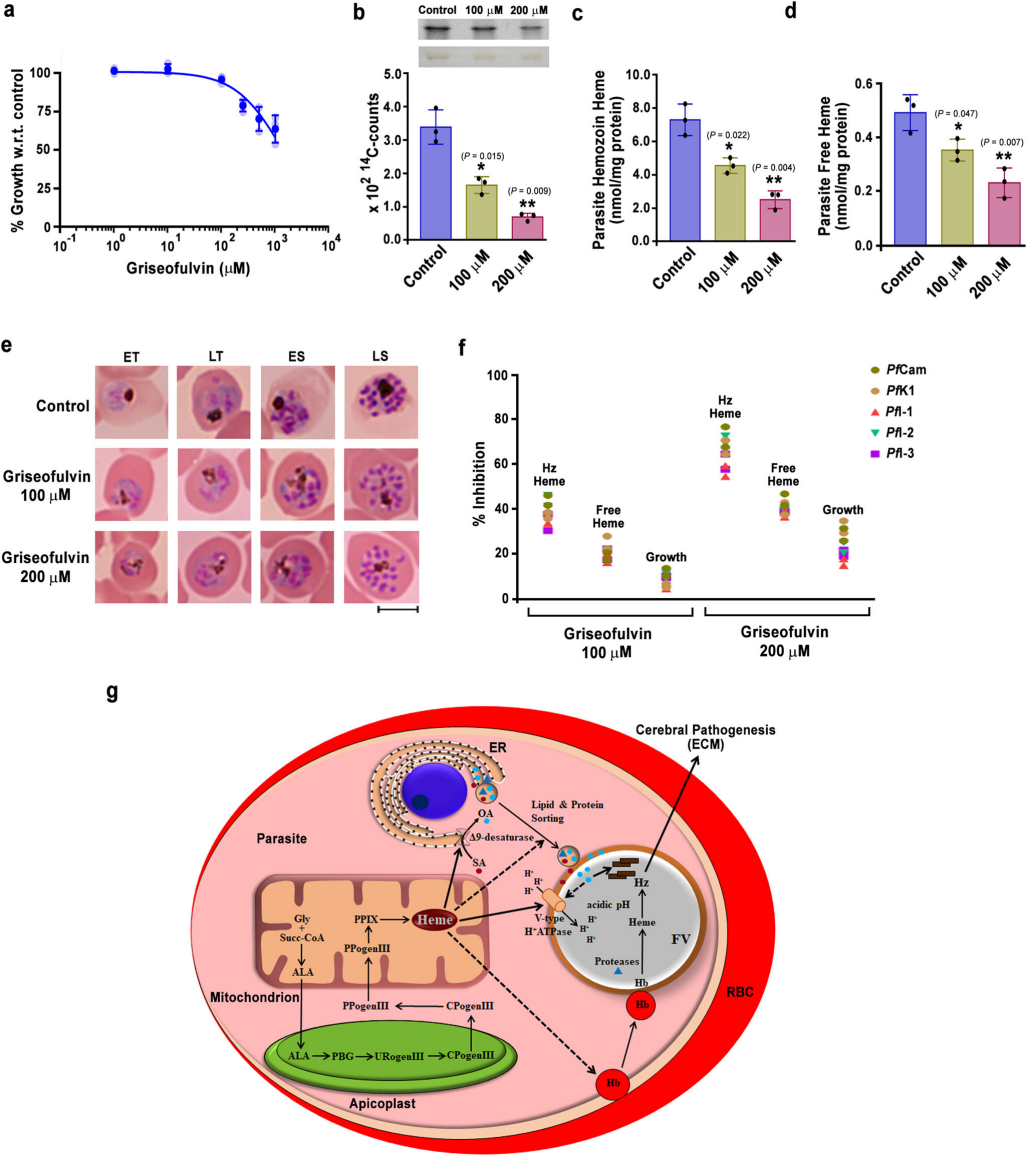
Effect of griseofulvin on *Pf* parasites and model depicting the role of de novo heme in regulating FV integrity and Hz formation. **a** Effect of griseofuvin on *Pf*3D7 parasite growth. The data (mean ± SD; R square = 0.8623) represent three different experiments. Individual data points are shown with the light shaded color. **b** Incorporation of ^14^C-ALA into *Pf*3D7 parasite de novo heme. The radioactive counts represent three different experiments (mean ± SD; *P < 0.05, **P < 0.01, unpaired *t*-test; two-sided). Individual data points are shown as black circles. Phosphorimager and scanned images of TLC performed for ^14^C-ALA labeled parasite free heme are also shown. Full-length scans are provided in the Supplementary Fig. 7. **c** *Pf*3D7 parasite Hz. **d** *Pf*3D7 parasite free heme. The data represent three different experiments (mean ± SD; *P < 0.05, **P < 0.01, unpaired *t*-test; two-sided). For **c** and **d**, individual data points are shown as black circles. **e** Giemsa-stained images of *Pf*3D7 parasites treated with griseofulvin. ET and LT early and late trophozoites; ES and LS early and late schizonts. Images were captured using 100x objective. Scale bar = 5 μm. *n* = 3 independent experiments. **f** Assessment of parasite Hz, free heme and growth in *Pf* clinical isolates. The data represent two different experiments for each clinical isolate. Percentage of inhibition was calculated with respect to untreated DMSO control. Individual data points are shown. **g** De novo heme pathway of malaria parasite and Hz formation in the FV are represented. Solid arrows indicate the effect of de novo heme on V-type H^+^-ATPase, FV pH and OA synthesis affecting hemozoin formation. Dashed arrows represent the probable effects of de novo heme on Hb endocytosis, and protein and lipid trafficking. Gly glycine, Succ-coA succinyl coenzymeA, ALA δ-aminolevulinic acid, PBG prophobilinogen, URogenIII uroporphyrinogen III, CPogenIII coproporphyrinogen III, PPogenIII protoporphyrinogen III, PPIX protoporphyrin IX, ER endoplasmic reticulum. Source data are provided as a Source Data file.

To examine the relevance of our findings in human malaria parasite, we assessed the ability of griseofulvin to inhibit Hz formation in *Pf*3D7 laboratory strain, artemisinin-resistant *Pf* clinical isolate from Cambodia (*Pf*Cam), multidrug-resistant *Pf* clinical isolate from Thailand (*Pf*K1) and three *Pf* clinical isolates that were collected from India (*Pf*I-1, −2 and −3). Unlike the approach of Smith *et al*^58^, we did not preload the host RBCs with griseofulvin for 3 days and use them for parasite invasion, to avoid any off-target effect. We directly treated infected RBCs having *Pf* late rings and early trophozoites with griseofulvin for 72 h and examined Hz content in the schizonts of next cycle. We first performed growth analysis in *Pf*3D7 using different concentrations of griseofulvin. Even at a 1 mM concentration, griseofulvin could not inhibit 50% of the parasite growth (Fig. 10a), a pattern that was consistent with the dispensable nature of parasite heme pathway and similar to that of succinylacetone - another heme pathway inhibitor^34^. We examined the ability of girseofulvin to inhibit de novo heme synthesis by performing ^14^C-ALA labeling at 100 and 200 μM concentrations. The mature RBCs used for culturing *Pf* parasites lack FC and do not synthesize heme. Therefore, heme labeling reflects the effect of griseofulvin on parasite de novo heme synthesis. There was almost 45 and 80% inhibition of ^14^C-ALA incorporation into parasite heme (Fig. 10b) in comparison to 5 and 15% of parasite growth inhibition at 100 and 200 μM concentrations, respectively. More importantly, griseofulvin could inhibit Hz formation in *Pf*3D7 cultures to the extent of ~40 and 70% at 100 and 200 μM concentrations, respectively (Fig. 10c). There was also a significant decrease in free heme levels (Fig. 10d). The decrease in Hz formation could be visualized in Giemsa-stained smears prepared from infected RBCs (Fig. 10e). Griseofulvin inhibited Hz formation in all the clinical isolates that were examined (Fig. 10f). All these evidences suggested that inhibition of de novo heme synthesis by griseofulvin affects Hz levels in human parasites as well, and griseofulvin has the potential to serve as an adjunct drug for cerebral and severe malaria in humans that needs to be evaluated in clinical trials.

## Discussion

Parasite heme pathway has remained enigmatic for more than two decades and the dichotomy between de novo heme and host-heme/porphyrin acquisition pathways in the asexual stages is obscure. Here, we provide an explanation as to why asexual parasite expresses “dispensable” heme pathway enzymes and synthesizes heme despite acquiring host heme. We show that de novo heme pathway is associated with disease virulence and it induces cerebral pathogenesis by promoting Hz formation. Hz is a key malarial PAMP associated with aberrant inflammatory responses, endothelial activation promoting pRBC sequestration, T cell infiltration and neuronal apoptosis. Upon phagocytosis, Hz can induce the production of pro-inflammatory cytokines and chemokines in neutrophils, monocytes, macrophages and DCs, leading to the increased expression of ICAM-1 in endothelial cells and enhanced sequestration of pRBCs. Hz can also trigger IL-1β production by activating NLRP3 inflammasome, and augment endothelial cell damage and loss of BBB integrity. Hz can induce proinflammatory responses in endothelial cells and activate coagulation and complement pathways^12,15^. The KO parasites synthesize less Hz and mice infected with KO parasites are devoid of cerebral complications. The plasma levels of IL-6, TNFα, IFNγ, G-CSF, CCL3 and CCL5 are significantly decreased in KO-infected mice. These cytokines and chemokines are known to be highly elevated in the serum samples of cerebral and severe malaria patients^15,59,60^. There is also a concomitant increase in anti-inflammatory cytokines such as IL-4, IL-10 and IL-13 in the KO-infected mice suggesting an over-all decrease in the systemic inflammation.

The transcript levels of TNFα, IFNγ, CXCL9, CXCL10, CCL2, CCL5 and CCL19 are low in the brain samples of KO-infected mice. CXCL9 and CXCL10 are highly induced in the brain of ECM mice and elevated levels of CXCL10 in plasma and cerebrospinal fluid predict fatal CM in humans^61^. CCL2, CCL5 and CCL19 associated with leukocyte migration are known to be induced by Hz in monocyte-derived DCs, macrophages and neutrophils^12,15,16,62^. Similar decrease was also observed in the transcript levels of ICAM-1, p-selectin, perforin, and granzyme B, the key adhesion and cytotoxic effector molecules. Low transcript levels of HO-1 in KO-infected mice might be due to less inflammation and decreased extracellular heme—the two potent HO-1 inducers^19,20^. Further, there is a decrease in the infiltration of CD8^+^ T cells expressing early activation marker CD69, and proinflammatory and cytotoxic effector molecules such as TNFα^+^, IFNγ^+^, CXCR3^+^, perforin and granzyme B that lead to endothelial leakage, BBB disruption and neuronal damage. There is also a reduction in phospho-NF-κB and NLRP3 inflammasome formation with decreased caspase-1 activation and less production of IL-1β - a key mediator of inflammation, infiltration of immune cells and neuronal apoptosis. Genetic complementation can restore Hz formation in the KO parasites and the mice infected with complemented KO parasites develop ECM.

The decrease in Hz synthesis is reflected in the reduced Hz load in the organs, and decreased free heme levels in the parasites and plasma samples of KO-infected mice. It is suggested that oxidized heme derived from host Hb release during schizont rupture contributes to free plasma heme responsible for severe malaria and CM pathogenesis^19–21^. Interestingly, plasma levels of Hb and heme scavenging proteins like hemopexin in KO-infected mice are comparable with WT-infected mice suggesting that the decreased plasma heme levels might be due to changes in parasite heme/Hz levels. While less Hz formation can be directly associated with reduced labile/free heme levels in KO parasites, the decrease in plasma free heme needs further investigations. Despite thriving in a heme-rich milieu, parasite maintains cytosolic free heme concentrations of ~1.6 μM, comparable with ~0.4–0.6 μM concentrations seen in mammalian cells^63^. It is possible that the parasite could have evolved with protein-/transporter-based mechanisms to dispose excess free heme from the cytosol and this could be the reason for reduced plasma free heme levels in KO-infected mice. For example, HRP capable of binding heme is secreted by the parasite. Altogether, our findings suggest that de novo heme in the blood stages can influence the levels of Hz and free heme—a key PAMP and DAMP associated with malaria pathogenesis.

Chloroquine acts predominantly by inhibiting host Hb-heme polymerization into Hz and it has been suggested that activation of artemisinin requires heme^44,45^. In agreement with decreased Hz formation, the sensitivity of FCKO parasites to chloroquine is compromised. In vitro chloroquine sensitivity is shown to be unaltered in heme pathway KO *Pf* cultures that are treated with chloroquine for 48 h and maintained using mature RBCs^36^. Our in vivo results with *Pb* suggest that FCKO parasites have greater propensity for recrudescence, despite their initial clearance with chloroquine is comparable to WT as observed in *Pf* cultures. It is known that *Pb* mutant parasites defective in Hb proteolysis producing less or no Hz can survive in reticulocytes exhibiting chloroquine resistance^52^. This could be the reason behind the early in vivo recrudescence of FCKO parasites as well. Nevertheless, the in vivo sensitivity to α,β-arteether remains unaltered. Our results suggest that albeit a ~50% decrease, the host-derived labile heme present in the cytosol of FCKO parasites is adequate to activate α,β-arteether and the role of de novo heme is confined to cerebral pathogenesis.

The host Hb degradation in asexual stage parasites can release as much as ~15 mM heme^63^ and therefore, utilization of host heme cannot account for almost 75% decrease in the Hz synthesis of KO parasites. Here, we show the unexpected role of de novo heme in influencing the detoxification of Hb-heme into Hz by regulating the FV integrity in asexual stages (Fig. 10g). FVs of FCKO parasites are compromised in terms of pH, lipid unsaturation and proteins associated with Hz formation. It is known that acidic pH of FV is critical for Hb digestion by proteases and subsequent Hz formation. The decrease in LysoTracker Deep Red uptake suggests that the FCKO FVs are less acidic. Our results also suggest the lower abundance of V-type H^+^ATPase subunits and berghepain-2 in FCKO FVs. V-type H^+^ATPase is a proton pump mainly responsible for maintaining the acidic pH of FV. Targeting V-type H^+^ATPase activity with concanamycin A or bafilomycin A1 can lead to the alkalinisation of FV and inhibition of parasite growth^51^. *Pb* has two isoforms of berghepain—berghepain-1 and −2 of which, berghepain-1 is associated with hepatic merozoite invasion and erythrocyte tropism, and berghepain-2 seems to be involved in Hb digestion^52,53^.

Further, we show reduction in OA synthesis of FCKO parasites with not much change in phospholipids or neutral lipids, suggesting an alteration in the degree of lipid unsaturation that can affect Hz formation. OA synthesis in malaria parasite is catalyzed by a heme-dependent, ER-localized, Δ9-desaturase and it does not occur in uninfected RBCs^48,49^. Our results suggest the specific role of de novo heme in OA synthesis that cannot be compensated by host Hb-heme. The levels of unsaturated fatty acids are also shown to stimulate the function of V-type H^+^ATPase in plants^64^. The decrease in OA synthesis and lipid unsaturation can affect other cellular processes such as membrane homeostasis, protein/lipid trafficking, protein folding, cell signaling etc^65^. Many of these can collectively contribute for the loss of FV integrity and responsible for the lower abundance of V-type H^+^ATPase subunits and berghepain-2 in FCKO FVs. Although host Hb-heme acquisition starts from late ring stages, it is possible that FV integrity and maturation depend on de novo heme. Our results provide functional insights on de novo heme that could be a miniscule in comparison with massive amounts of heme derived from host Hb. Intriguingly, we show it is the de novo heme that influences the detoxification of host heme into Hz and disease pathogenesis. Despite the ability of blood stage parasites to acquire host Hb-heme for survival, lack of de novo heme synthesis renders them less virulent.

It would be interesting to examine whether (i) de novo heme regulates other metabolic pathways or organellar functions and influences the trafficking mechanisms associated with host-heme or Hb uptake and (ii) compartmentalization of de novo and Hb-heme make them functionally different. Similarly, the role of Δ9-desaturase in cerebral pathogenesis and the requirement of ‘dispensable’ biosynthetic heme for ER-localized cytochrome b5-dependent Δ9-desaturase activity need to be understood. Cytochrome b5 also supports minimal ETC activity and pyrimidine biosynthesis that are indispensable for asexual stages. *Plasmodium* has at least two putative isoforms of cytochrome b5 annotated in PlasmoDB and one of them seems to be non-essential for asexual growth. It would be of interest to examine whether the non-essential cytochrome b5 participates in Δ9-desaturase activity, and its heme requirement is satisfied by biosynthetic heme and could not be compensated by Hb-heme. Further, the accessibility of Hb-heme to various organelles and its incorporation in hemoproteins may vary. Besides its role in disease pathogenesis, Hz is also associated with parasite sequestration, manipulation of host immune responses etc. that can exert selection pressure for the existence of molecular mechanisms to optimize its formation. Altogether, our findings suggest the importance of “dispensable” de novo heme pathway in the asexual stages despite its non-essentiality for parasite growth.

The fatality rates in malaria do not correlate with parasite clearance and therefore, targeting parasite virulence becomes important. The malaria deaths that occur despite treating the infected individuals with ACTs and the decreasing efficacy of ACTs that lead to delayed parasite clearance underscore the need of an adjunct therapy in the initial stages of ACT treatment. Until now, the attempts to develop adjunct therapies with candidates acting mainly on host responses did not succeed in clinical trials and only a few candidates are in the pipeline for evaluation in HCM^4,66^. We show that de novo heme in the blood stages can serve as a target for malaria pathogenesis. Griseofulvin—a FDA-approved antifungal drug prevents ECM in mice and delays death due to anemia by inhibiting parasite heme synthesis. The ability of griseofulvin to prevent disease pathogenesis is observed despite the absence of any antimalarial treatment. Griseofulvin treatment does not affect the parasite growth suggesting that ECM protection is not because of the inhibition of parasite mitosis. This is in concurrence with a clinical trial conducted in malaria-infected humans where, griseofulvin treatment did not affect in vivo parasite growth although NMPP formation occurred within the parasite. The participants were rescued early with ACT and therefore, no observations were made on disease severity^58^. Griseofulvin can also prevent ECM in *Pb*-infected mice treated with α,β-arteether at the late stages of infections with onset of neurological symptoms. It inhibits parasite heme synthesis and Hz formation in *Pf* clinical isolates as well.

Our study identifies a unique approach of targeting Hz through de novo heme to mitigate parasite virulence. Since de novo heme pathway is dispensable for asexual stages, there is a possibility of less selection pressure to result in resistance development. Griseofulvin can significantly affect host heme and porphyrin metabolism only at dosages of prolonged duration that are several fold higher than the therapeutic dose, although any subtle impact at lower doses cannot be ruled out. Conversely, malaria treatment is only for a shorter duration. Given the central role played by Hz in malaria pathogenesis, repurposing of griseofulvin can serve as an excellent adjunct to ACTs for cerebral and severe malaria that needs to be evaluated in clinical trials.

## Methods

### Ethics statement

All the studies involving mice were carried out with the approval of Institutional Animal Ethics Committee (ILS/IAEC-57-AH/JAN-16), Institute of Life Sciences, Bhubaneswar, according to the national guidelines framed by “The Committee for the Purpose of Control and Supervision of Experiments on Animals (CPCSEA)”. *P. falciparum* clinical samples were collected with the approval of Institutional Ethics Committee (IEB) / Institutional Review Board (IRB) (94/HEC/19), Institute of Life Sciences, Bhubaneswar.

### Routine propagation of *Pb* in mice and CM experiments

*Pb*ANKA WT and KO parasites were propagated in C57BL/6 male mice of 7–8 weeks old. Peripheral blood parasitemia was monitored by performing light microscopy for Giemsa-stained thin blood smears prepared from tail vein blood. When the blood parasitemia was around 10%, 10^5 ^*P. berghei* ANKA WT or 10^5^/10^7^ ALAS/FC KO parasites were collected and injected intraperitoneally in 7–8 weeks old C57BL/6 male naïve mice to initiate ECM experiments. The male mice were preferred for uniformity in the experiments and the results on parasite growth, ECM protection, decrease in heme and hemozoin levels, and prevention of ECM by griseofulvin were reproducible in 7–8 weeks old C57BL/6 female mice as well. The experiments for which C57BL/6 female mice were used are mentioned in the figure legends. Balb/c male mice of 7–8 weeks old were used to examine growth phenotype, heme and hemozoin levels, and for raising polyclonal sera. Breeding and maintenance of mice were performed at the animal house facility of Institute of Life Sciences, Bhubaneswar, under standard conditions of 25 ± 3 °C temperature, 40–50% relative humidity, and 12 h light/12 h dark cycle. Growth curve analysis was carried out by monitoring the blood parasitemia. The development and progression of ECM were monitored by examining RMCBS for neurological symptoms as described^67^. On the respective days, WT- or KO-infected C57BL/6 mice were assessed for coordination (gait and balance), exploratory behavior (motor performance), strength and tone (body position and limb strength), reflexes and self-preservation (touch escape, pinna reflex, toe pinch and aggression) and hygiene-related behavior (grooming). Each parameter was scored for 0–2 (0 as the lowest and 2 as the highest), for a total RMCBS score of 20. To assess BBB integrity, Evans blue uptake assays were carried out by injecting 200 μl of 2% Evans blue in PBS intravenously and examining the extravasation of dye after one h in the brain of the infected mice that were  transcardially perfused with PBS. The extent of BBB damage was quantified by incubating the brain samples in formamide at 37 °C for 48 h, extracting the Evans blue and measuring the absorbance at 620 nm^19^.

### Histological and immunofluorescence analyses of cerebral pathology

For H&E staining to assess vascular blockage, hemorrhages and demyelination, brain samples were fixed with formalin for 72 h at room temperature. After dehydrating with ethanol and treating with xylene, paraffin embedded blocks were made and sections of 7 μm thickness were prepared using Leica RM2125RT rotary microtome. The sections were then processed and stained with H&E using standard protocols. Immunohistochemical analysis of IgG extravasation in the brain sections was carried out as described^11^. The brain sections of 30 μm thickness were antigen retrieved by treating them at 95 °C for 30 min in sodium citrate buffer pH 6.0, followed by blocking with 3% H_2_O_2_ at room temperature for 30 min to prevent endogenous peroxidase activity. The sections were then incubated with HRP-conjugated goat anti-mouse IgG (Abcam, ab97023) at 1:250 dilution in PBS containing 0.3% Triton X-100 and 0.1% BSA, followed by developing with diaminobenzidine tertrahydrochloride (Vector Labs, SK-4100) and counterstaining with hematoxilin (HiMedia, S058). Immunoflourescence analysis of brain sections for parasite sequestration was carried out as described^11^ by fixing the brain samples in 4% paraformaldehyde in PBS containing 20% sucrose for 24 h at 4 °C and cryoprotecting them for 48 h in PBS containing 20% sucrose. Coronal sections of 30 μm thickness were prepared using Leica CM1850 cryostat microtome and antigen retrieval was carried out by treating them at 95 °C for 30 min in sodium citrate buffer pH 9.0. After blocking with 1% BSA, the sections were incubated with anti-CD31 mouse monoclonal antibody (1:200 dilution) conjugated with Alexa Fluor 594 (SantaCruz, sc-376764) and anti-*Pb*GAPDH rabbit polyclonal serum (1:100 dilution) or anti-mouse CD3 rat monoclonal antibody (Thermo Fisher Scientific, 14-0032-82; 1:100 dilution) for 16 h at 4 °C. The sections were then treated with FITC-conjugated donkey anti-rabbit IgG (SantaCruz, sc-2090; 1:200 dilution) or FITC-conjugated goat anti-rat IgG (SantaCruz, sc-2011; 1:200 dilution), followed by 4′,6-diamidino-2-phenylindole (DAPI) staining. Anti-mouse β-APP rabbit polyclonal antibody (Thermo Fisher Scientific, 51–2700) was used in 1:200 dilution. All the images were captured using Olympus IX83 microscope with DP73 high-performance camera.

### Heme, Hz, hemoglobin and hemopexin estimations

Free heme levels in the plasma samples of WT- and KO-infected mice were quantified using Hemin colorimetric assay kit (BioVision, K672) as per the manufacturer’s protocol. The assay is specific for free heme and it utilizes peroxidase activity of hemin to facilitate the conversion of a colorless probe to a strongly colored compound with absorbance at 570 nm. The quantification of free heme in the parasite lysates was carried out by resuspending the parasite pellets in 5 volumes of hypotonic lysis buffer containing 5 mM Tris pH 7.5 with protease inhibitors and incubating them in ice for 30 min. The lysates were then centrifuged at 20,000 *g* for 20 min, 4 °C, and the supernatants obtained were used for free heme estimation as mentioned above for the plasma samples. The protein content of the hypotonic lysates was measured by Micro BCA protein assay kit (Thermo Fisher Scientific, 23235) and the free heme levels were expressed per mg of protein. The Hz content of the WT and KO parasite pellets was estimated as described^68^. The parasite pellet was resuspended in 1 ml of 100 mM sodium acetate buffer, pH 5.0 and left at 37 °C for overnight, followed by centrifugation at 10,000 *g* for 5 min. The resultant pellet was resuspended in 100 mM Tris buffer pH 8.0 containing 2.5% SDS and incubated at 37 °C for 30 min, followed by centrifugation at 10,000 *g* for 5 min. The pellet obtained was washed once with 100 mM sodium bicarbonate pH 9.2 and then with distilled water. The final Hz pellet was dissolved in 100 mΜ NaOH containing 2.5% SDS, and the absorbance was measured at 405 nm. The supernatants of sodium acetate and Tris SDS steps were collected to estimate the protein content and heme content of the Hz was expressed per mg of total protein. To estimate the Hz content in the spleen and liver of the WT- and KO-infected mice, 50 mg tissue of the respective organs was homogenized in 50 mM Tris pH 8.0 containing 50 mM NaCl, 5 mM CaCl_2_ and 1% Triton X-100, and incubated for 12 h at 37 °C in the presence of proteinase K. The lysates were sonicated and centrifuged at 15,000 *g* for 30 min. The pellets obtained were resuspended with 100 mM sodium bicarbonate containing 2% SDS and sonicated, followed by centrifugation at 15,000 *g* for 15 min. After repeating this step thrice, the pellets were solubilized in 100 mM NaOH containing 2% SDS and 3 mM EDTA, and the absorbance was measured at 405 nm^69^. In parallel, the parasite load in the organs was examined by quantitative PCR (qPCR) analysis carried out with *Pb18SrRNA* primers for the total RNA isolated from 30 mg tissue of the organs using RNeasy Plus Mini Kit (Qiagen, 74134). After normalizing with respect to the parasite load, the total heme content of the Hz isolated was expressed per mg weight of the organ. Hemoglobin and hemopexin levels in the plasma samples of WT- and KO-infected mice were measured by Mouse Hemoglobin (Abcam, ab157715) and Mouse Hemopexin (Abcam, ab157716) ELISA kits as per the manufacturer’s protocols.

### *Pb*ALASKO^*Luc*^, *Pb*FCKO^*Luc*^, *Pb*Control^*Luc*^ and *Pb*FCKO^*+FC*^ parasites

*Pb*ALASKO^*Luc*^ and *Pb*FCKO^*Luc*^ parasites were generated by transfecting WT parasites with GOMO-GFP-Luc plasmid containing GFP-Luc-expressing cassette with m-cherry flanked on either side by 5′- and 3′-UTR regions of the respective genes. The primers utilized were similar to the earlier ones that were used for generating *Pb*ALASKO and *Pb*FCKO^34^ except that the restriction sites used for forward and reverse primers were *SacII* and *NotI* for 5′ UTRs and *XhoI* and *KpnI* for 3′ UTRs, respectively. For *Pb*Control^*Luc*^ parasites, the following sets of forward and reverse primers were used to amplify the 5′- and 3′-UTRs of small subunit ribosomal RNA (*ssurRNA*): 5′UTR (F) – GCCACCGCGGGAAATACGACCAATATGTAATTATTGGATAATAATTG; 5′UTR (R) – GCCCGCGGCCGCCTACTGGCAAGATCAACCAGGTTACTATATATA; 3′UTR (F) – GCCACTCGAGGAGGCTTATCCTTCCTGATAAAGTG; 3′UTR (R) – GCCCGGTACCCAAAATACTAACCCACTATGTGCAATGTGC. The restriction sites are underlined. The 5′ and 3′ UTRs were cloned into GOMO-GFP-Luc plasmid and transfection was carried out in WT parasites. The transfections were performed with 4D-Nucleofector using P5 Primary Cell 4D-Nucleofector reagent (Lonza, V4XP-5012). The transfected parasites were selected with pyrimethamine and clonal selection was carried out by performing limiting dilutions. To perform *FC* complementation, *Pb*FCKO^*Luc*^-infected mice were treated with 5-fluorocytosine to remove the pyrimethamine-selectable h*DHFR*-y*FCU* fusion gene from *Pb*FCKO^*Luc*^ parasites. The recovery of selectable marker-free *Pb*FCKO^*Luc*^ parasites was confirmed by the loss of m-cherry fluorescence. The parasites were then transfected with pL1102 plasmid in which RFP under *Pbeef1α* promoter was replaced with *Pb*FC under its native promoter. In brief, pL1102 plasmid was digested with *AflII* and *XbaI* to remove RFP and its promoter, followed by cloning of −906 to +1547 sequence representing *Pb*FC promoter and its coding sequence that was PCR amplified with the following forward and reverse primers: GCAACTTAAGTGGAGCATTTTCATGCTCATAAATATATTC and GCAATCTAGATTACCAGCCACTTAGATTTTTTTCAATAAT. The restriction sites are underlined. The transfected *Pb*FCKO^*Luc*^ parasites having FC reintroduced in *c-ssurRNA* locus (*Pb*FCKO^*+FC*^) were selected with pyrimethamine and clonal selection was carried out by limiting dilution.

### In vivo bioluminescence imaging

In vivo bioluminescence imaging for mice infected with WT and KO parasite lines expressing luciferase was carried out as described^70^. For whole body imaging, 7–8 weeks old C57BL/6 mice were injected with Luc-expressing 10^5^ WT or 10^7^ KO parasites. On day 8 post-infection, mice were injected intraperitoneally with D-luciferin substrate (100 mg/kg animal weight in 200 μl of PBS), VivoGlo (Promega, P1041), and imaged after 5 min using in vivo Imaging System IVIS Lumina *XR* with medium binning, 10 s exposure and 12.5 FOV, under XGI-8 gas anesthesia system. For ex vivo imaging, transcardial perfusion was carried out for infected mice with cold PBS after injecting D-luciferin, and the organs were dissected out and imaged.

### Analyses of cytokines, chemokines and chemokine receptors

Bio-Plex assays for cytokines and chemokines were carried out in Bio-Plex 200 system using Bio-Plex Pro Mouse Cytokine Grp I Panel 23-Plex assay kit (Bio-Rad, M60009RDPD) following manufacturer’s protocol. The plasma samples utilized for the assays were prepared from the infected mouse blood collected on day7/8 post-infection. For transcript levels, total RNA was isolated from the brain samples of WT and KO-infected mice that were collected after a thorough perfusion with cold PBS. qPCR analyses were performed using QuantiFast SYBR Green RT-PCR Kit (Qiagen, 204154) on StepOne Real-Time PCR System (Applied Biosystems). The primers used were listed in Supplementary Table 2. Expression levels were normalized with GAPDH and fold changes for the transcripts of KO-infected mice with respect to WT were calculated using 2^(−ΔΔ*Ct*)^ method. To examine the transcript levels of parasite proteins, total RNA was isolated from the parasite pellets and the expression levels were normalized with respect to parasite GAPDH.

### Flow cytometry

Flow cytometry analyses of T cells in the brain samples of WT- and KO-infected mice were carried out as described^71^. Mice were anesthetized and transcardially perfused with PBS, and the brain samples were dissected out and harvested in RPMI-1640 medium containing 10% FBS. For preparing single cell suspensions, the samples were minced and digested in RPMI-1640 medium containing 0.05% Collagenase D and 2U/ml DNase I for 30 min at room temperature, and passed through 70 μm nylon cell strainer, followed by 5 min of incubation on ice. Brain homogenates were then overlaid on 30% Percoll cushion and centrifuged at 400 *g* for 20 min at room temperature. The leukocyte pellets obtained were resuspended in 1 ml of RBC lysis buffer (155 mM NH_4_Cl, 10 mM NaHCO_3_ and 0.1 mM EDTA; pH 7.3) and incubated on ice for 5 min to remove any residual RBCs. The pellets were then washed with RPMI-1640, counted and stained for various markers. For intracellular markers like TNFα, IFNγ, perforin and granzyme B, staining was carried out after fixing the cells with 2% paraformaldehyde. Single fluorochrome-stained cells were used to compensate for the spectral overlap. Flow cytometry was performed with BD LSRFortessa and CytoFLEX S and the data were analyzed using FlowJo™ v10.6.1. The following fluorescent dye-conjugated antibodies were used for staining: anti-mouse CD3-FITC (clone 17A2; Thermo Fisher Scientific, 11-0032-82; 0.25 μg/10^6^ cells in 100 μl volume), anti-mouse CD4-PE (clone RM4-5; Thermo Fisher Scientific, 12-0043-82; 0.125 μg/10^6^ cells in 100 μl volume), anti-mouse CD8-PerCP-Cyanine5.5 (clone 53–6.7; Thermo Fisher Scientific, 45-0081-82; 0.25 μg/10^6^ cells in 100 μl volume), anti-mouse CD69-Brilliant Violet 421 (clone H1.2F3; BioLegend, 104527; 0.25 μg/10^6^ cells in 100 μl volume), anti-mouse CXCR3-PE (clone CXCR3-173; Thermo Fisher Scientific, 12-1831-82; 0.25 μg/10^6^ cells in 100 μl volume), anti-mouse Perforin-PE (clone S16009A; BioLegend, 154305; 0.50 μg/10^6^ cells in 100 μl volume), anti-mouse Granzyme B-APC (clone NGZB; Thermo Fisher Scientific, 17-8898-82; 0.125 μg/10^6^ cells in 100 μl volume), anti-mouse IFNγ-eFluor 450 (clone XMG1.2; Thermo Fisher Scientific, 48-7311-82; 0.50 μg/10^6^ cells in 100 μl volume), anti-mouse TNFα-eFluor 450 (clone MP6-XT22; Thermo Fisher Scientific, 48-7321-82; 0.25 μg/10^6^ cells in 100 μl volume).

### FV isolation and analyses

In *P. berghei*, the Hz containing vacuoles tend to remain as small discrete vacuoles until the late schizont stage and they coalesce only at the time of schizont rupture. Because of this, purifying the FVs for *P. berghei* trophozoites is difficult using the standard percoll protocol that is followed for *P. falciparum*, wherein the small vacuoles coalesce in the late ring stages itself. Hence, we resorted ourselves to the isolation of FVs that are released during the schizont rupture as described with slight modifications^72^. Infected blood containing WT or FCKO parasites was collected around 22:00 h, centrifuged at 1000 g for 5 min to remove plasma and buffy coat, and washed twice with RPMI-1640 medium containing 10% FBS. The washed cells were resuspended in 10 volumes of RPMI-1640 medium containing 10% FBS and then incubated at 37 °C for overnight in a CO_2_ incubator. The maturation of schizonts was monitored by Giemsa smears and the FVs released in the culture supernatant during schizont rupture were collected in the next day around 09:00 h. In brief, the cultures were centrifuged at 200 *g* for 5 min to remove the RBCs. After repeating this step twice, the supernatant devoid of RBCs was centrifuged at 400 *g* for 10 min to collect the FVs that are free of merozoites. The FV pellet was then washed twice with PBS by centrifuging at 3000 *g* for 3 min and stored at −20 °C. The purity of FVs was tested under microscope with 100x objective.

### Microscopy analyses of FV pH, lipid staining, Hz content and Hz dynamics

To examine the LysoTracker Deep Red uptake in the live parasites, 10–20 μl of infected blood was collected from tail vein of the infected mice when the peripheral blood parasitemia was around 5–10% and resuspended in heparinised RPMI-1640 medium containing 10% FBS. After washing twice, the cells were incubated with 100 nM LysoTracker Deep Red (Thermo Fisher Scientific, L12492) for 30 min at 37 °C in RPMI-1640 medium containing 10% FBS. The cells were then washed thrice with RPMI-1640 medium without phenol red, resuspended in the same medium and examined immediately under 100x objective using Olympus IX83 microscope with DP73 high-performance camera at 1600 × 1200 resolution using TRITC filter. BODIPY 493/503 (Thermo Fisher Scientific, D3922) and Nile Red (Sigma-Aldrich, 19123) staining for parasitized RBCs were carried out as described^73^. 10–20 μl of infected mouse blood was collected from the tail vein in Hank’s balanced salt solution (HBSS) and centrifuged at 2000 *g* for 3 min. The cell pellet obtained was resuspended in 200 μl of HBSS containing BODIPY 493/503 (10 μg/ml) or Nile Red (2 μg/ml) and incubated at 37 °C for 30 min. The cells were then washed twice and resuspended in HBSS. The images were acquired under 100x objective using Olympus IX83 microscope with DP73 high-performance camera at 1600 × 1200 resolution using FITC/TRITC filters. The images for WT and KO were acquired under identical exposure conditions and the fluorescent signal intensities were quantified using ImageJ software v1.52a. To examine the Hz content, bright field and DIC images were taken under 100× objective. Live imaging of Hz dynamics was carried out under 100x objective by acquiring 15 frames per second at 1600 × 1200 resolution. The video files were processed using VSDC video editor v6.4.2.108 and VideoPad v4.30 NCH softwares.

### Labeling studies with ^14^C-ALA, ^32^P-orthophosphoric acid and ^14^C-SA

[4-^14^C]-ALA (ARC 1550), ^32^P-orthophosphoric acid (ARC 0103) and [1-^14^C]-SA (ARC 025) were procured from American Radiolabeled Chemicals, Inc. The infected blood samples were collected, centrifuged at 1000 g for 5 min to remove plasma and buffy coat, and washed twice with RPMI-1640 medium containing 10% FBS. The washed cells were resuspended in 10 volumes of RPMI-1640 medium containing 10% FBS and then incubated at 37 °C in a CO_2_ incubator with the respective radioactive compounds. For ^14^C-ALA labeling, blood samples were collected from griseofulvin treated and control WT-infected mice around 16:00 h and the labeling was carried out for 9 h at a radioactivity of 1 μCi/ml. The infected RBCs were then centrifuged, washed with PBS and the parasites were isolated by saponin (Sigma-Aldrich, S4521) treatment. After washing the parasite pellet with PBS for four times, free heme present in the parasites was extracted using ethylacetate:glacial acetate (4:1) followed by 1.5 N HCl and water washes to remove porphyrins and ALA as described^34^. The upper phase was separated, dried under nitrogen stream, dissolved in methanol and analyzed by TLC on silica gel using the mobile phase 2,6-lutidine and water (5∶3 v/v) in ammonia atmosphere. The intensity of radiolabelling was scanned using Amersham Typhoon 5 Biomolecular Imager by exposing the TLC sheets to phosphorimager screen and the radioactive counts were measured using PerkinElmer MicroBeta^2^ 2450 Microplate Counter. For ^32^P-orthophosphoric acid and ^14^C-SA labeling, the blood sample collection and incubation were carried out as mentioned for the FV isolation. In addition to the isolation of the secreted FVs, the infected RBC pellets were subjected to saponin treatment for the isolation of the radiolabelled total parasites. The labeling was carried out for ^32^P-orthophosphoric acid and ^14^C-SA at radioactivities of 50 μCi/ml and 2 μCi/ml, respectively. All these experiments were typically carried out in a total volume of 4 ml, carefully matching in terms of parasitized RBCs and hematocrit between WT and FCKO.

### Lipid analyses

Lipid extraction for phospholipid analysis was carried out as described^74^. FV and parasite pellets were extracted with chloroform:methanol (2:1) and washed twice with water. The lower organic phase was collected and analyzed by TLC on silica gel using the mobile phase chloroform:ethanol:water:tri-ethylamine (30:35:7:35 v/v). 5 μl of the sample was used to take radioactive counts. For fatty acid analysis, the lipids extracted as mentioned above were dried, dissolved in 50 μl of toluene and subjected to mild methanolysis/methylation by the addition of 375 μl of methanol and 75 μl of 8% HCl in methanol. After incubating at 45 °C for 16 h, FAMEs were extracted by the addition of 250 μl of hexane and 250 μl of water^75^. The hexane layer containing FAMEs was analyzed by TLC on silica gel impregnated with 0.5% methanolic silver nitrate using the mobile phase light petroleum ether:acetone:formic acid (97:2:1 v/v) to separate FAMEs derived from unsaturated fatty acids^76^. In this separation, SAME lacking double bond migrates faster as an uppermost top band followed by OAME and other unsaturated FAMEs that are separated based on their degree of unsaturation and the length of fatty acyl chain. The respective standards were used for all the lipids. 5 μl of the hexane layer was used to take radioactive counts. For GC-MS analysis, 1 µl of hexane containing the FAMEs was injected in split mode (50:1). The GC-MS analysis was performed using Agilent 7890B GC coupled with 240 Ion Trap MS. The column used for analysis was Agilent VF-5MS (30 m length × 0.25 mm internal diameter (ID) x 0.25 µm film thickness). The oven temperature was programmed from 140 °C (5 min hold), increased at rate of 4 °C/min to 240 °C. Helium was used as carrier gas with 1 ml/min flow rate. The mass spectrometer was operated in full scan mode from 40 to 500 m/z and NIST library search was performed to identify the compounds using Agilent Workstation Software v7.0.1. The set integration parameters of NIST search and identification include peak width value of 4, tangent % value of 10, slope sensitivity (Signal/Noise) value of 50 with peak size reject counts of 5000 and a default threshold of 500.

### Proteomics analyses

To examine the protein content of the FVs from WT and FCKO parasites, proteins were extracted from three different preparations of WT and FCKO FVs using 25 mM ammonium bicarbonate containing 6 M urea. The respective WT and FCKO FV protein extracts were pooled separately, followed by treatment with DTT and iodoacetamide. The urea concentration was then reduced to 0.6 M by performing dilution with 25 mM ammonium bicarbonate. For LC-MS/MS, in-solution trypsin (SCIEX) digestion was carried out for 200 μg total protein, followed by sample clean up with Waters Oasis SPE cartridge. The samples were subjected to micro flow reverse-phase LC in Eksigent Ekspert Nano LC 425 system (SCIEX) that was directly connected to a tandem quadrupole time-of-flight SCIEX TripleTOF 5600+ ESI-mass spectrometer. The samples were concentrated using a SCIEX Micro Trap Cartridge (Chrome XP; C18-CL, 5-μm, 120-Å pore size). The trap cartridge was washed with 0.1% (v/v) formic acid and 2% (v/v) acetonitrile in water, and the concentrated peptides were then separated using a SCIEX capillary reverse-phase column (ChromeXP, 3C18-CL-120, 3 μm, 120 Å and 0.3 × 150 mm) at a flow rate of 5 μl/min using the following solvents: solvent A—98% water and 2% acetonitrile containing 0.1% formic acid (v/v), and solvent B—98% acetonitrile and 2% water containing 0.1% formic acid (v/v). The gradient parameters were set at 2 to 50% of solvent B in 28 min, followed by 50 to 90% of solvent B in 1 min, sustaining 90% of solvent B for 3 min and then 90–5% B in 0.5 min with a final re-equilibration with 2% of solvent B for 2.5 min. Mass spectra and tandem mass spectra were recorded in positive-ion and high-sensitivity mode with a full scan resolution of 35,000 (full width at half maximum) and the ion source was operated with the following parameters: IonSpray Voltage Floating (ISVF) = 5500; Ion Source Gas 1 (GS1) = 25; Ion Source Gas 1 (GS2) = 22; Curtain Gas Flow (CUR) = 30. The precursor ions were fragmented in a collision cell containing nitrogen as a collision gas. The calibrations for TOF MS spectra and TOF MS/MS spectra were performed by injecting 100 fmol beta-galactosidase digest (SCIEX). The peptide spectra were recorded over a mass/charge (m/z) range of 350 to 1250, and MS/MS spectra were recorded over an m/z range of 150–1600 in data-dependent acquisition (DDA) mode. Data acquisition was achieved using Analyst TF1.7.1. software and DDA was performed to obtain MS/MS spectra for the 15 most abundant parent ions following each survey MS1 scan (250-ms acquisition time per MS1 scan and 50-ms acquisition time per MS/MS). Dynamic exclusion features were set to an exclusion mass width of 50 mDa and an exclusion duration of 6 s. The acquired MS/MS data were annotated using Paragon algorithm (ProteinPilot Software Version 5.0.2, SCIEX) against the reference proteomes of *Plasmodium berghei* (UP000074855, Taxonomy 5823; UP000219974, Taxonomy 5821) available at Uniprot (https://www.uniprot.org/) with the following parameters: TripleTOF 5600 instrument; alkylation of cysteines by iodoacetamide; trypsin enzyme digestion; ID Focus on biological modifications and the detected protein threshold [Conf] set at >10%. The mass spectrometry proteomics data of LC-MS/MS have been deposited to the ProteomeXchange Consortium via the PRIDE partner repository with the dataset identifier PXD031736.

Fourplex iTRAQ labeling was carried out as per the manufacturer’s protocol (iTRAQ kit, AbSciex, Cat No. 4381664). FVs were solubilized in 0.5 M triethylammonium bicarbonate (TEAB) containing 0.1% sodium dodecyl sulfate (SDS) and 5 mM reducing reagent tris-(2-carboxyethyl) phosphine (TCEP), and incubated at 60 °C for 1 h followed by treatment with methyl methane thiosulfonate (MMTS) for 10 min at room temperature. 50 and 100 μg total protein were used to perform trypsin digestion at 37 °C for 16 h. Two sets of four-plex reactions were carried out for three different preparations of WT and FCKO FVs along with two internal controls. The digested peptides of WT and FCKO FVs were labeled with either 115, 116 or 117 isobaric tag, and isobaric tag 114 was used exclusively to label the internal controls that had equivalent representation of all the samples for normalization. The samples were spiked with bovine serum albumin (BSA) to ensure the loading accuracy. The samples labeled with different isobaric tags were pooled together as one single sample for individual reaction set and vacuum concentrated. After performing sample clean up with Waters Oasis SPE cartridge, the peptides were separated and the mass spectra and tandem mass spectra were recorded as mentioned for LC-MS/MS analysis using Eksigent Ekspert Nano LC 425 system (SCIEX) coupled with a tandem quadrupole time-of-flight SCIEX TripleTOF 5600+ ESI-mass spectrometer. The peptide spectra were recorded over a mass/charge (m/z) range of 350 to 1250, and MS/MS spectra were recorded over an m/z range of 100 to 1600. Protein identification and quantification were performed using Paragon algorithm (ProteinPilot Software Version 5.0.2, SCIEX) with a false discovery rate (FDR) ≤1% against the reference proteome of *Mus musculus* (UP000000589, Taxonomy 10090) available at Uniprot (https://www.uniprot.org/). Proteins having peptides identified with 95% confidence or above were considered for further analysis. Proteins were quantified by summing the reporter ion intensities across the spectra matching to each protein and normalizing them with respect to the internal control labeled with isobaric tag 114. The normalized reporter ion intensities were used to calculate the fold changes of α and β chains of mouse hemoglobin in *Pb*FCKO FVs with respect to *Pb*WT FVs. The mass spectrometry proteomics data of iTRAQ have been deposited to the ProteomeXchange Consortium via the PRIDE partner repository with the dataset identifier PXD031738.

### Griseofulvin and α,β-arteether treatment in mice

Griseofulvin (Sigma-Aldrich) was prepared by dissolving 1 or 2 mg in 40 μl DMSO and then making up the volume to 200 μl with 10% solutol HS 15 (Sigma-Aldrich) in saline. The mixture was vortexed thoroughly for 10 min to form an emulsion and injected intraperitoneally into the mice. All single dose injections were carried out at 06:00 h and double dose injections were carried out at 06:00 h and 18:00 h for the respective days. Control mice were injected with the solvent. α,β-arteether (E MAL (75 mg/ml); Themis Medicare Ltd) was administered intramuscularly. For experiments involving the combination of α,β-arteether and griseofulvin, α,β-arteether was administered intramuscularly at 06:00 h on day 6, followed by two doses of griseofulvin at 24 h interval (at 09:00 h on day 6 and 7).

### In vitro *Pf* culture maintenance and treatment

*Pf* cultures were maintained at 37 °C in RPMI-1640 medium (Gibco, 23400-013) containing 0.5% AlbuMAX-II (Thermo Fisher Scientific, 11021037) using O^+ve^ RBCs of 5% hematocrit under 90% N_2_, 5% O_2_ and 5% CO_2_. Griseofulvin was dissolved in DMSO and added at appropriate concentrations to 5 ml cultures that were synchronized for late rings and early trophozoites with 5% sorbitol (w/v) (Sigma-Aldrich, 240850). The final concentration of DMSO in cultures did not exceed 0.2% (v/v). The initial parasitemia at the time of griseofulvin addition was around 0.5–1%. The cultures were harvested after 72 h when the parasites were predominantly in schizonts and saponin lysis was carried out to isolate the parasites from infected RBCs. ^14^C-ALA labeling in griseofulvin-treated *Pf* cultures was carried out for 24 h by adding ^14^C-ALA (1 μCi/ml culture volume) at 48 h post-treatment. ^14^C-radiolabelled de novo heme present in the parasites was extracted using ethylacetate:glacial acetate (4:1), and heme and Hz analyses were carried out as mentioned for *Pb*. The parasite growth was monitored by performing flow cytometry for the infected RBCs stained with 0.5X SYBR Green I (Thermo Fischer Scientific, S7563) for 30 min at 37 °C and by examining Giemsa smears prepared for the cultures. The *Pf* clinical strains used for griseofulvin treatment were revived from the glycerolyte stocks prepared directly from the patient blood using RPMI-1640 medium containing 10% human O^+ve^ serum. The clinical samples were collected with the approval of Institutional Ethics Committee (IEB) / Institutional Review Board (IRB) (94/HEC/19), Institute of Life Sciences, Bhubaneswar, from febrile patients who visited Ispat General Hospital, Rourkela, India, after obtaining their informed consent. The *Pf* infections were confirmed by microscopic examination of Giemsa-stained thick and thin blood smear, rapid diagnostic test and PCR for *Pf18S rRNA*. *Pf*Cam clinical isolate (IPC 5202) was from a human patient with malaria in Battambang province, western Cambodia. *Pf*K1 was isolated from a patient in Thailand.

### Western blot analyses and other procedures

Western analyses were carried out using standard procedures. Brain samples were homogenized in 50 mM Tris-Cl buffer, pH 7.5 containing 5 mM EDTA, 50 mM NaCl, 5 mM DTT, 0.1% Np-40, 50 mM NaF, 1 mM PMSF, 1 mM Na_3_VO_4_, and 1x Halt Protease Inhibitor Cocktail (Thermo Fisher Scientific), followed by centrifugation at 18,000 *g* for 20 min at 4 °C. The supernatants were collected and quantified for total protein. The following antibodies were used: anti-mouse NF-κB p65 (Thermo Fisher Scientific, 14-6731-81; 1:1000 dilution); anti-mouse Phospho-NF-κB p65 (Ser536) (Thermo Fisher Scientific, MA5-15160; 1:1000 dilution); anti-mouse NLRP3 (Thermo Fisher Scientific, PA5-20838; 1:1000 dilution); anti-mouse Phospho-NLRP3 (Ser295) (Thermo Fisher Scientific, PA5-105071; 1:1000 dilution); anti-mouse Caspase 1 (Thermo Fisher Scientific, 14-9832-82; 1:500 dilution); anti-mouse Cleaved Caspase-1 (Asp296) (Cell Signaling Technology, 89332; 1:1000 dilution); anti-mouse IL-1β (Thermo Fisher Scientific, 701304; 1:250 dilution); anti-mouse Cleaved IL-1β (Cell Signaling Technology, 52718; 1:1000 dilution) and anti-mouse β-Actin (Cell Signaling Technology, 3700; 1:2000 dilution). Blots were developed using Pierce ECL Western Blotting Substrate (Thermo Fisher Scientific). Polyclonal sera raised against recombinant *Pf*FC in mouse and recombinant *Pb*GAPDH in rabbit were used at 1:500 dilution. Polyclonal sera raised against recombinant *Pb*V-type H^+^ATPase subunits B and G in mouse were used at 1:1000 dilution. The parasite lysates for Western analysis of FC were prepared by resuspending the parasite pellets isolated by saponin lysis in 50 mM Tris pH 8.0 containing 500 mM NaCl, 0.5% Triton X-100 and protease inhibitors, followed by sonication in mice. The lysates were then centrifuged at 15,000 *g* for 20 min at 4 °C and the supernatants were used. For Western analysis of V-type ATPase subunits, parasite and food vacuole pellets were resuspended in 50 mM Tris pH 8.0 containing 1 M NaCl, 1% Triton X-100 and 1x Halt Protease Inhibitor Cocktail, followed by sonication in ice. In vitro cytoadherence studies were performed under static conditions using mouse brain endothelial cell line b.End5 (Sigma-Aldrich, 96091930) pre-stimulated with recombinant mouse TNF-α (50 ng/ml) (Sigma-Aldrich, T7539) for 16 h as described^77^. Parasitized RBCs infected with *Pb*Control^*Luc*^ and *Pb*FCKO^*Luc*^ trophozoites and schizonts expressing m-cherry were purified using Nycodenz density gradient centrifugation. In vitro cytoadherence of parasitized-RBCs was examined after 90 min of incubation with endothelial cells at 37 °C. To assess the parasite load in peripheral circulation, qPCR analyses were carried out using *Pb*GAPDH primers with the total RNA prepared from 100 μl of whole blood collected by retro-orbital bleeding from the infected mice treated with respective drugs. The Δ*C*_*t*_ and ΔΔ*C*_*t*_ values for the parasite load were calculated with respect to mouse GAPDH.

### Statistical analyses

All the graphs were plotted using GraphPad Prism Version 7.00 software and the statistical analyses were carried out using unpaired Welch’s *t*-test (two-sided), two-way ANOVA and log-rank (Mantel–Cox) test. For two-way ANOVA, Tukey test was performed and the multiple comparisons were corrected using statistical hypothesis testing. n.s not signiicant, *P < 0.05, **P < 0.01, ***P < 0.001. The non-linear regression fit was carried out for inhibitor versus response curve and the R-squared value was determined using GraphPad Prism Version 7.00 software.

### Reporting summary

Further information on research design is available in the Nature Research Reporting Summary linked to this article.

### Supplementary information

Supplementary Information

Description of Additional Supplementary Files

Supplementary Data 1

Supplementary Data 2

Supplementary Data 3

Supplementary Movie 1

Supplemantary Movie 2

Supplementary Movie 3

Reporting Summary

### Source data

Source Data

## Data Availability

Source data are provided with this paper. The data that support the findings of this study are also available in figshare^78^. 10.6084/m9.figshare.19354475. For proteomics analyses, reference proteomes of *Plasmodium berghei* (UP000074855, Taxonomy 5823; UP000219974, Taxonomy 5821) and *Mus musculus* (UP000000589, Taxonomy 10090) were accessed through Uniprot website. The mass spectrometry proteomics data of LC-MS/MS and iTRAQ have been deposited to the ProteomeXchange Consortium via the PRIDE partner repository with the dataset identifiers PXD031736 and PXD031738, respectively. Source data are provided with this paper.
