# Author Correction: Malaria parasite heme biosynthesis promotes and griseofulvin protects against cerebral malaria in mice

**DOI:** 10.1038/s41467-024-49627-w

**Published:** 2024-06-25

**Authors:** Manjunatha Chandana, Aditya Anand, Sourav Ghosh, Rahul Das, Subhashree Beura, Sarita Jena, Amol Ratnakar Suryawanshi, Govindarajan Padmanaban, Viswanathan Arun Nagaraj

**Affiliations:** 1 Infectious Disease Biology, Institute of Life Sciences, Bhubaneswar, 751023 Odisha India; 2 School of Biotechnology, Kalinga Institute of Industrial Technology, Bhubaneswar, 751024 Odisha India; 3 Regional Centre for Biotechnology, Faridabad, 121001 Haryana India; 4 Department of Biochemistry, Indian Institute of Science, Bangalore, 560012 Karnataka India

**Keywords:** Mechanisms of disease, Parasite biology, Malaria

Correction to: *Nature Communications* 10.1038/s41467-022-31431-z, published online 12 July 2022

The original version of this Article contained an error in Fig. 5a, in which the Giemsa staining image of ‘*Pb*ALASKO’ in the first row was inadvertently duplicated in the second row of ‘*Pb*FCKO’. This has been corrected in both the PDF and HTML versions of the Article.

