# Supplementary material for: Malaria parasite heme biosynthesis promotes and griseofulvin protects against cerebral malaria in mice
Source: Nat Commun. 2022 Jul 12;13:4028. doi: 10.1038/s41467-022-31431-z (PMC9276668; doi:10.1038/s41467-022-31431-z)
Supplement: Supplementary file 1 — Supplementary Information [file 41467_2022_31431_MOESM1_ESM.pdf]

Supplementary Information

Malaria parasite heme biosynthesis promotes and griseofulvin protects against cerebral malaria in mice

Manjunatha Chandana, Aditya Anand, Sourav Ghosh, Rahul Das, Subhashree Beura, Sarita Jena, Amol Ratnakar Suryawanshi, Govindarajan Padmanaban and Viswanathan Arun Nagaraj

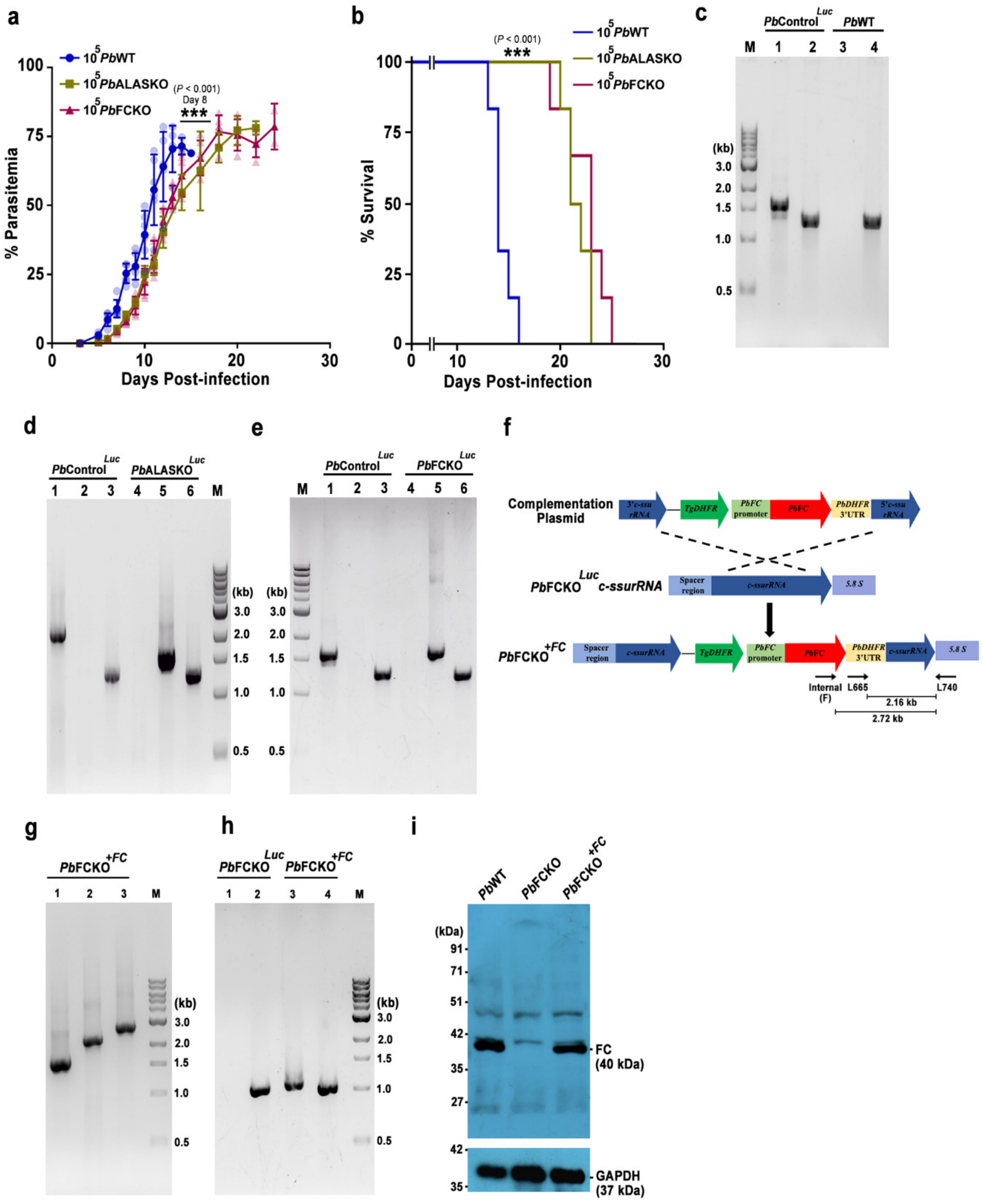

Supplementary Figure 1: Growth characterization of heme pathway KO parasites in Balb/c mice and confirmation of site-specific integrations in *Pb*Control^{Luc}, *Pb*KO^{Luc} and *Pb*FCKO^{+FC} parasites. a, Growth analysis in Balb/c mice (n=6). 10^5 parasites were used for infections. The data represent two different batches (mean $\pm$ SD; ***$P < 0.001$, Two-way ANOVA). Individual data points are shown with the respective light shaded colors. **b,** Mortality curves (n=6) (***$P < 0.001$, log-rank (Mantel-Cox) test). **c,** Confirmation of site-

specific integration in *Pb*Control^{Luc}. Lane M: 1 kb ladder; Lane 1 and 3: integration product (1.56 kb) obtained using *Pb*Control Integration (F) primer and GFP (R) primer; Lane 2 and 4: *GAPDH* product (1.25 kb). *Pb*Control Integration (F) primer is 100 bp upstream to the 5'-UTR sequence of *ssurRNA*. **d,** Confirmation of site-specific integration in *Pb*ALASKO^{Luc}. Lane 1 and 4: *ALAS* product (2.11 kb); Lane 2 and 5: integration product (1.60 kb) obtained using *Pb*ALAS Integration (F) primer and GFP (R) primer; Lane 3 and 6: *GAPDH* product (1.25 kb); Lane M: 1 kb ladder. *Pb*ALAS Integration (F) primer is 888 bp upstream to the coding region of *ALAS*. **e,** Confirmation of site-specific integration for *Pb*FCKO^{Luc}. Lane M: 1 kb ladder; Lane 1 and 4: *FC* product (1.54 kb); Lane 2 and 5: integration product (1.62 kb) obtained using *Pb*FC Integration (F) primer and GFP (R) primer; Lane 3 and 6: *GAPDH* product (1.25 kb). *Pb*FC Integration (F) primer is 906 bp upstream to the coding region of FC. The integration (F) primer sequences are absent in the 5'UTRs of the plasmids used for the generation of KOs. **c-e,** n = 2 independent experiments. **f,** Recombination strategy followed to perform *FC* complementation in *Pb*FCKO^{Luc} parasites. *FC* with its native promoter is reintroduced into *c-ssurRNA* of *Pb*FCKO^{Luc} parasites through stable integration. Black arrows represent the position of primers used for the confirmation of site-specific integration in *Pb*FCKO^{+FC} parasites. **g,** Genomic DNA PCR confirmation for *FC* complementation in *Pb*FCKO^{+FC} parasites. Lane 1: *FC* product (1.54 kb); Lane 2: Integration at *c-ssurRNA* locus confirmed with L665 (*Pb*DHFR 3'UTR-specific) and L740 (*5.8 S*-specific) primers (2.16 kb); Lane 3: Integration at *c-ssurRNA* locus confirmed with *Pb*FC internal (F) and L740 primers (2.72 kb); Lane M: 1 kb ladder. n = 3 independent experiments. **h,** RT-PCR confirmation for *FC* complementation. Lane 1 and 3: *FC* product (1.05 kb); Lane 2 and 4: GAPDH product (1.01 kb). *Pb*FCKO^{Luc} was used as a control. Lane M: 1 kb ladder. n = 3 independent experiments. **i,** Western confirmation for *FC* complementation using parasite FC antibody. 50 μg of total protein was used. For control, the same blot was stripped and re-probed with parasite GAPDH antibody. n = 2 independent experiments. Source data are provided as a Source Data file.

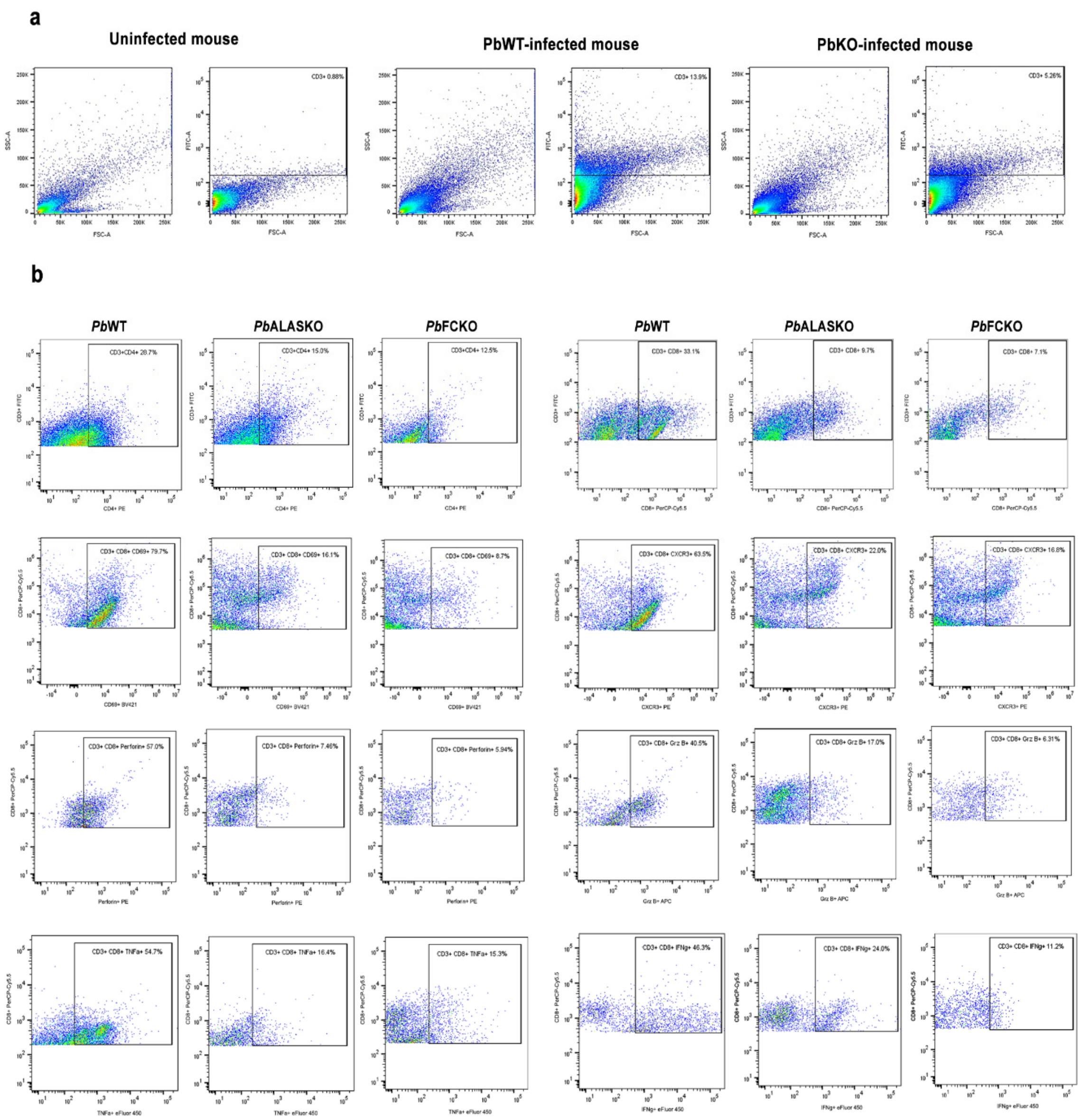

Supplementary Figure 2: Representative flow cytometry plots. a, Gating strategy used for flow cytometry experiments. Representative plots for uninfected control, *Pb*WT- and *Pb*KO-infected mouse brain samples are provided. The total events acquired from the side scatter and forward scatter were gated for CD3 positivity. The gated CD3$^+$ cells were further analysed for CD3$^+$ CD4$^+$ and CD3$^+$ CD8$^+$ double positive T cells, and CD3$^+$ CD8$^+$ CD69$^+$, CD3$^+$ CD8$^+$ CXCR3$^+$, CD3$^+$ CD8$^+$ perforin$^+$, CD3$^+$ CD8$^+$ granzyme B$^+$, CD3$^+$ CD8$^+$ TNFα$^+$ and CD3$^+$ CD8$^+$ IFNγ$^+$ triple positive T cells. **b,** Dot plots from flow cytometry analyses representing T cells in the brain samples of WT- and KO-infected mice. The data represent CD3$^+$ CD4$^+$ and CD3$^+$ CD8$^+$ double positive T cells, and CD3$^+$ CD8$^+$ CD69$^+$, CD3$^+$ CD8$^+$ CXCR3$^+$, CD3$^+$ CD8$^+$ perforin$^+$, CD3$^+$ CD8$^+$ granzyme B$^+$, CD3$^+$ CD8$^+$ TNFα$^+$ and CD3$^+$ CD8$^+$ IFNγ$^+$ triple positive T cells, obtained for *Pb*WT-, *Pb*ALASKO- and *Pb*FCKO-infected mice. Due to the less yield of T cells, the experiments have to be performed in different batches for various markers and the plots provided are the representative ones from different batches. Gating and spectral-overlap compensation were performed for the individual samples based on the corresponding

unstained and single-fluorophore stained cells. For *Pb*KO-infected mouse brain samples, cells were acquired from the pooled single cell suspensions since the desired triple positive T cell populations were less. The percentages shown for $CD3^+$ $CD4^+$ and $CD3^+$ $CD8^+$ double positive T cells are with respect to the $CD3^+$ cells. The percentages shown for $CD3^+$ $CD8^+$ $CD69^+$, $CD3^+$ $CD8^+$ $CXCR3^+$, $CD3^+$ $CD8^+$ perforin$^+$, $CD3^+$ $CD8^+$ granzyme B$^+$, $CD3^+$ $CD8^+$ TNFα^+ and $CD3^+$ $CD8^+$ IFNγ^+ triple positive T cells are with respect to the $CD3^+$ $CD8^+$ double positive T cells. Based on the percentages of different subsets and the total number of cells obtained in the single cell suspensions, the number of respective cells per mouse brain were calculated.

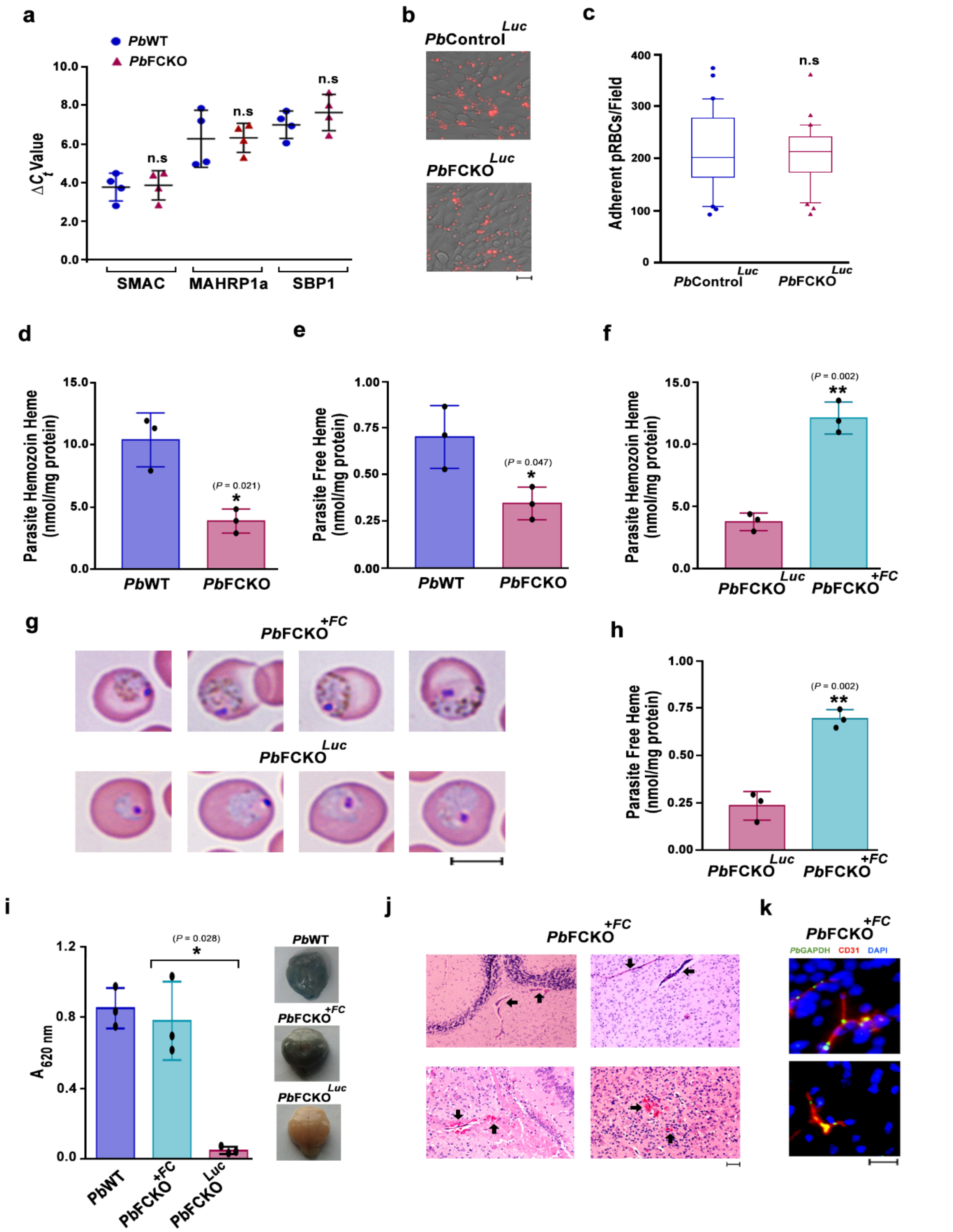

Supplementary Figure 3: *In vitro* sequestration of *Pb*FCKO^{Luc} parasites, Hz and free heme levels in *Pb*FCKO parasites from Balb/c mice, restoration of Hz and free heme levels in *Pb*FCKO^{+FC} parasites, and cerebral pathogenesis in *Pb*FCKO^{+FC}-infected mice. **a,** qPCR analysis of the RNA expression levels for SMAC, MAHRP1a and SBP1 in *Pb*WT and *Pb*FCKO parasites. The ΔC_t values obtained with respect to parasite GAPDH are plotted. n = 4 independent experiments (mean $\pm$ SD; n.s - not significant, unpaired t-test; two-sided).

b, Live fluorescence imaging for *in vitro* cytoadherence of *Pb*Control^{Luc}- and *Pb*FCKO^{Luc}-pRBCs with TNFα-prestimulated mouse brain endothelial cells. m-cherry fluorescence of the parasites was captured using 20x objective. Scale bar = 20 µm. n = 3 independent experiments. **c,** Number of adherent pRBCs per field counted using 20x objective. n = 3 independent experiments. Box and whisker plots display 10th and 90th percentile as the whiskers, 25th-75th percentile as the boxes and median as the centre line (n.s - not significant, unpaired t-test; two-sided). Minima and maxima for *Pb*Control^{Luc}- and *Pb*FCKO^{Luc}-pRBCs are 93 and 373, and 94 and 361, respectively. **d,e,** Hz and free heme levels in *Pb*WT and *Pb*FCKO parasites isolated from Balb/c mice (n=3) (mean $\pm$ SD; *$P<0.05$, unpaired t-test; two-sided). **f,** Hz levels in *Pb*FCKO^{Luc} and *Pb*FCKO^{+FC} parasites isolated from C57BL/6 mice (n=3). (mean $\pm$ SD, **$P<0.01$, unpaired t-test; two-sided) **g,** Giemsa-stained images of *Pb*FCKO^{+FC} parasites showing Hz content. Images were captured using 100x objective. Scale bar = 5 µm. n = 3 independent experiments. **h,** Free heme levels in *Pb*FCKO^{Luc} (n=3) and *Pb*FCKO^{+FC} (n=3) parasites (mean $\pm$ SD, **$P<0.01$, unpaired t-test; two-sided). **i,** Quantification of Evans blue in the brain samples of C57BL/6 female mice infected with *Pb*WT (n=3), *Pb*FCKO^{Luc} (n=3) and *Pb*FCKO^{+FC} (n=3) parasites (mean $\pm$ SD; *$P<0.05$, unpaired t-test; two-sided). Whole brain images are shown for Evans blue extravasation. **j,** H&E staining of the brain sections from *Pb*FCKO^{+FC}-infected mice. Black arrows indicate intracerebral and petechial hemorrhages, and thrombosed blood vessels. Images were captured using 10x objective. Scale bar = 50 µm. n = 2 independent experiments. **k,** Immunofluorescence analysis of parasite accumulation in brain sections of *Pb*FCKO^{+FC} parasite-infected mice. Images were captured using 20x objective. Scale bar = 20 µm. n = 2 independent experiments. For **d, e, f, h** and **i,** individual data points are shown as black circles. Source data are provided as a Source Data file.

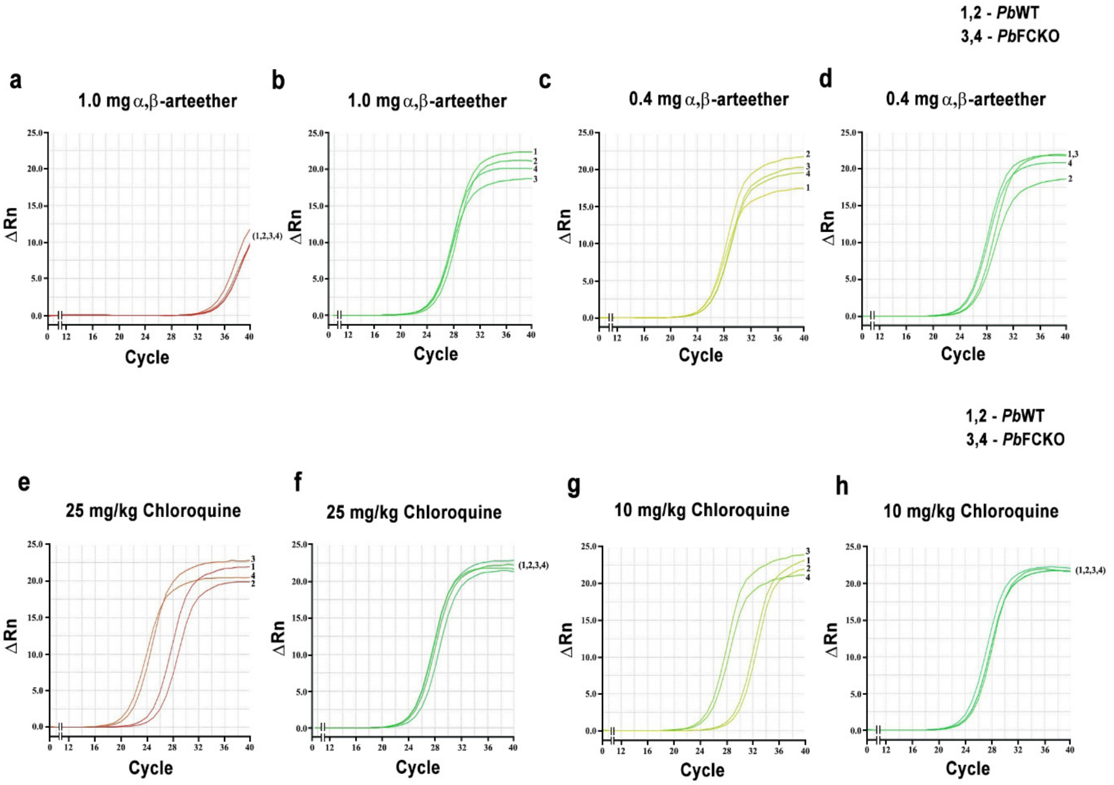

Supplementary Figure 4: Assessment of parasite load in α,β-arteether and chloroquine treated WT- and KO-infected mice. a, qPCR analyses of parasite load using *Pb*GAPDH primers for the infected mice treated with 1 mg/mouse dose of α,β-arteether with total RNA isolated from the whole blood. The amplification curves represent primer dimers indicating the absence of detectable parasites. **b,** qPCR analyses for mouse GAPDH control. **c,** qPCR analyses of parasite load using *Pb*GAPDH primers for the infected mice treated with 0.4 mg/mouse dose of α,β-arteether. **d,** qPCR analyses for mouse GAPDH control. The $\Delta\Delta C_t$ value obtained for *Pb*FCKO with respect to *Pb*WT for 0.4 mg dosage was 1.43 $\pm$ 0.16 (mean $\pm$ SD). **e,** qPCR analyses of parasite load using *Pb*GAPDH primers for the infected mice treated with 25 mg/kg dose of chloroquine. **f,** qPCR analyses for mouse GAPDH control. **g,** qPCR analyses of parasite load using *Pb*GAPDH primers for the infected mice treated with 10 mg/kg dose of chloroquine. **h,** qPCR analyses for mouse GAPDH control. The $\Delta\Delta C_t$ values obtained for *Pb*FCKO with respect to *Pb*WT were -4.02 $\pm$ 0.91 and -4.19 $\pm$ 0.43 for 25 mg/kg and 10 mg/kg doses, respectively (mean $\pm$ SD). The C_t values obtained for mouse GAPDH were comparable between *Pb*WT- and *Pb*FCKO-infected mice. 1,2 - *Pb*WT-infected mice; 3,4 - *Pb*FCKO-infected mice.

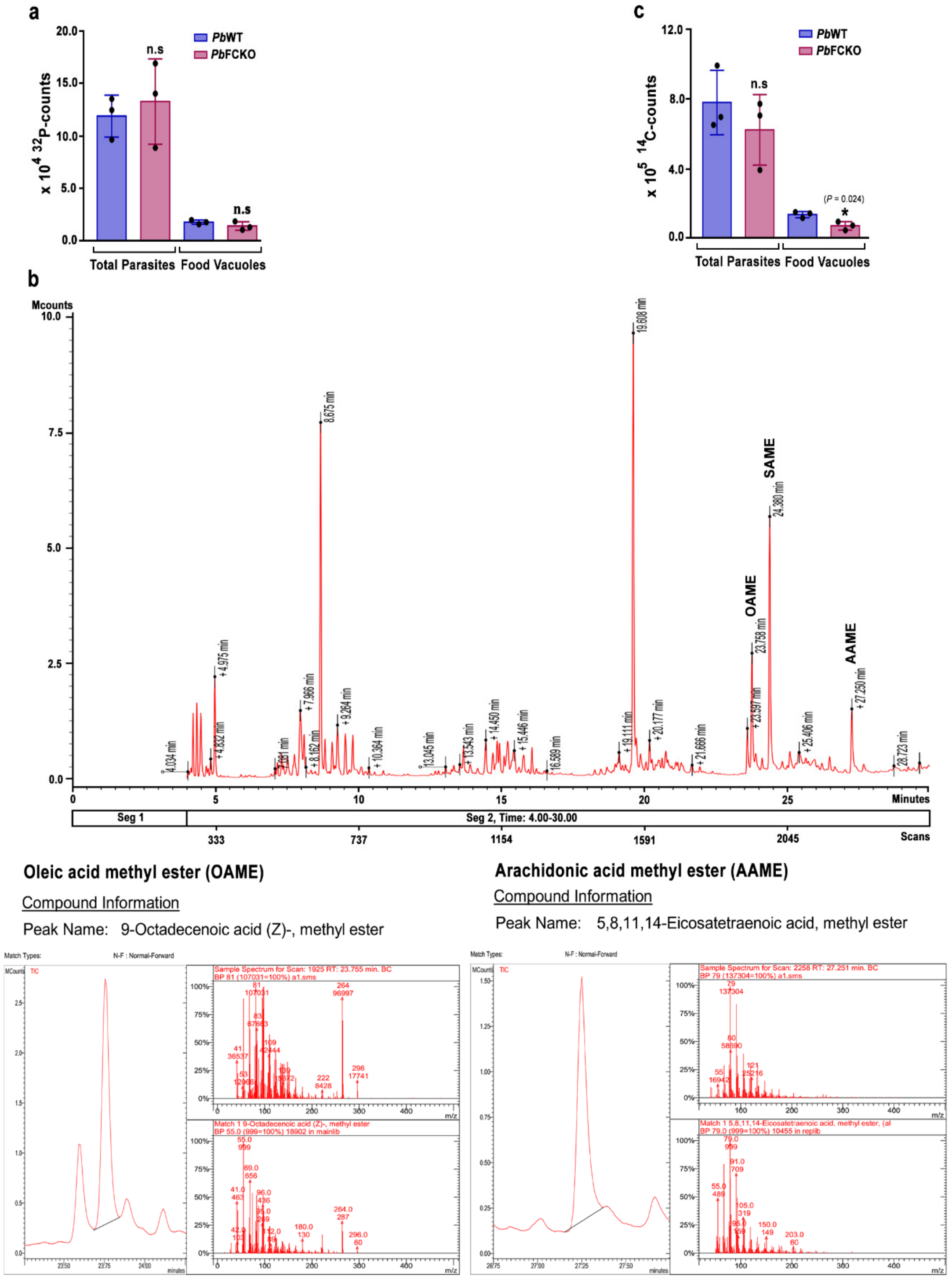

Supplementary Figure 5: Radiolabelling of phospholipids and FAMEs, and GC-MS analysis of FAMEs prepared from *Pb* FVs. a, Radioactive counts for ^{32}P-orthophosphoric acid radiolabelled phospholipids of WT and FCKO total parasites and FVs. The data represent three different experiments (mean $\pm$ SD; n.s - not significant, unpaired t-test; two-sided). Individual data points are shown as black circles. **b,** GC-MS analysis of FAMEs prepared from *P. berghei* FVs. The total ion chromatogram for the entire run and the extracted chromatogram

peaks for the methyl esters of oleic acid and arachidonic acid and their mass spectra are shown. OAME - oleic acid methyl ester; SAME - stearic acid methyl ester; AAME - arachidonic acid methyl ester. The entire set of compounds that could be identified and their respective peak areas and retention times are given in Supplementary Table 1. **c,** Radioactive counts for ^{14}C-SA radiolabelled FAMEs of WT and FCKO total parasites and FVs. The data represent three different experiments (mean $\pm$ SD; n.s - not significant, **P*<0.05, unpaired t-test; two-sided). Individual data points are shown as black circles. Source data are provided as a Source Data file.

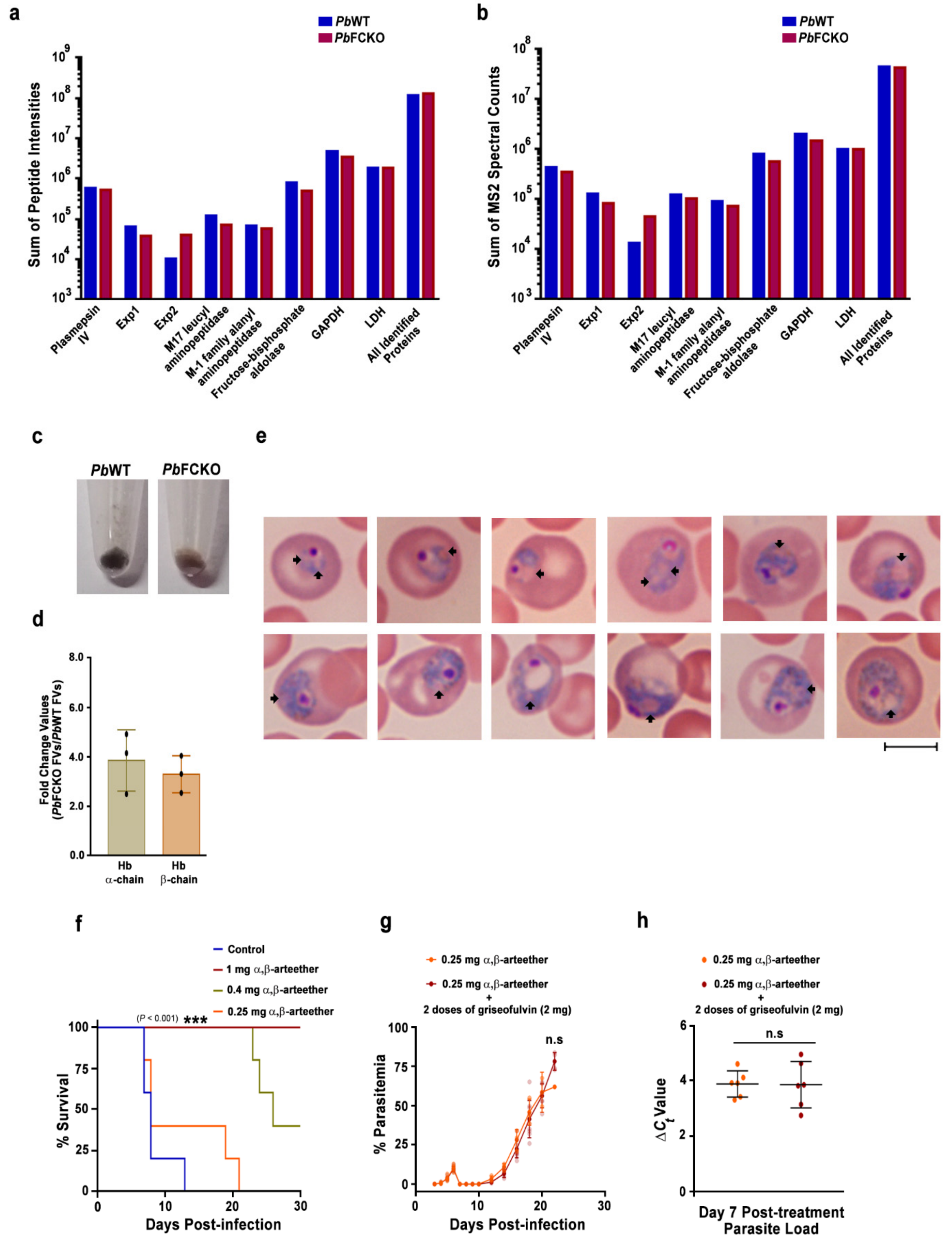

Supplementary Figure 6: Proteomic analyses of *Pb* FVs and assessment of α,β-arteether and griseofulvin combination in WT-infected C57BL/6 mice. a, Peptide intensities of FV proteins. Data represent the sum of peptide intensities of eight proteins known to be present in the FV preparations including the characteristic FV proteins. Sum of peptide intensities of all the identified proteins is also shown. **b,** MS2 spectral counts of FV proteins. Data represent the

sum of MS2 spectral counts for the respective proteins. Sum of MS2 spectral counts of all the identified proteins is also shown. LDH - lactate dehydrogenase. For **a** and **b,** all the peptides identified for the respective proteins with 95% confidence or above are included. **c,** Images of FV preparations from *Pb*WT and *Pb*FCKO parasites. **d,** Fold change values for α and β chains of Hb in *Pb*FCKO FVs calculated based on the ratio of reporter ion intensities. Two sets of four-plex iTRAQ reactions were carried out for 50 and 100 μg total protein from three different FV preparations of WT and FCKO parasites. The data represent mean $\pm$ SD values for the fold changes and the individual data points are shown as black circles. The reporter ion intensities obtained for Hb α and β chains for two sets of iTRAQ reactions are provided in Supplementary Data 3. **e,** Giemsa-stained images of *Pb*FCKO parasites showing the presence of translucent vesicles. Such translucent vesicles was observed in several *Pb*FCKO parasites. Images were captured using 100x objective. Scale bar = 5 μm. n = 3 independent experiments. **f,** Dose optimization studies performed with α,β-arteether. For each dose, 5 female mice were used. (****P*<0.001, log-rank (Mantel-Cox) test). **g,** Growth analysis of mice (n = 6) treated with α,β-arteether and griseofulvin combination. 10^5 parasites were used to initiate WT infections. The data represent two different batches (mean $\pm$ SD; n.s - not significant, Two-way ANOVA). Individual data points are shown with the respective light shaded colors. **h,** qPCR analysis of parasite load in *Pb*WT-infected mice treated with α,β-arteether and griseofulvin combination. ΔC_t values obtained for parasite GAPDH with respect to mouse GAPDH for α,β-arteether treatment alone (n=6) and α,β-arteether treatment in combination with griseofulvin (n=6) are plotted. (mean $\pm$ SD; n.s - not significant, unpaired t-test; two-sided). Source data are provided as a Source Data file.

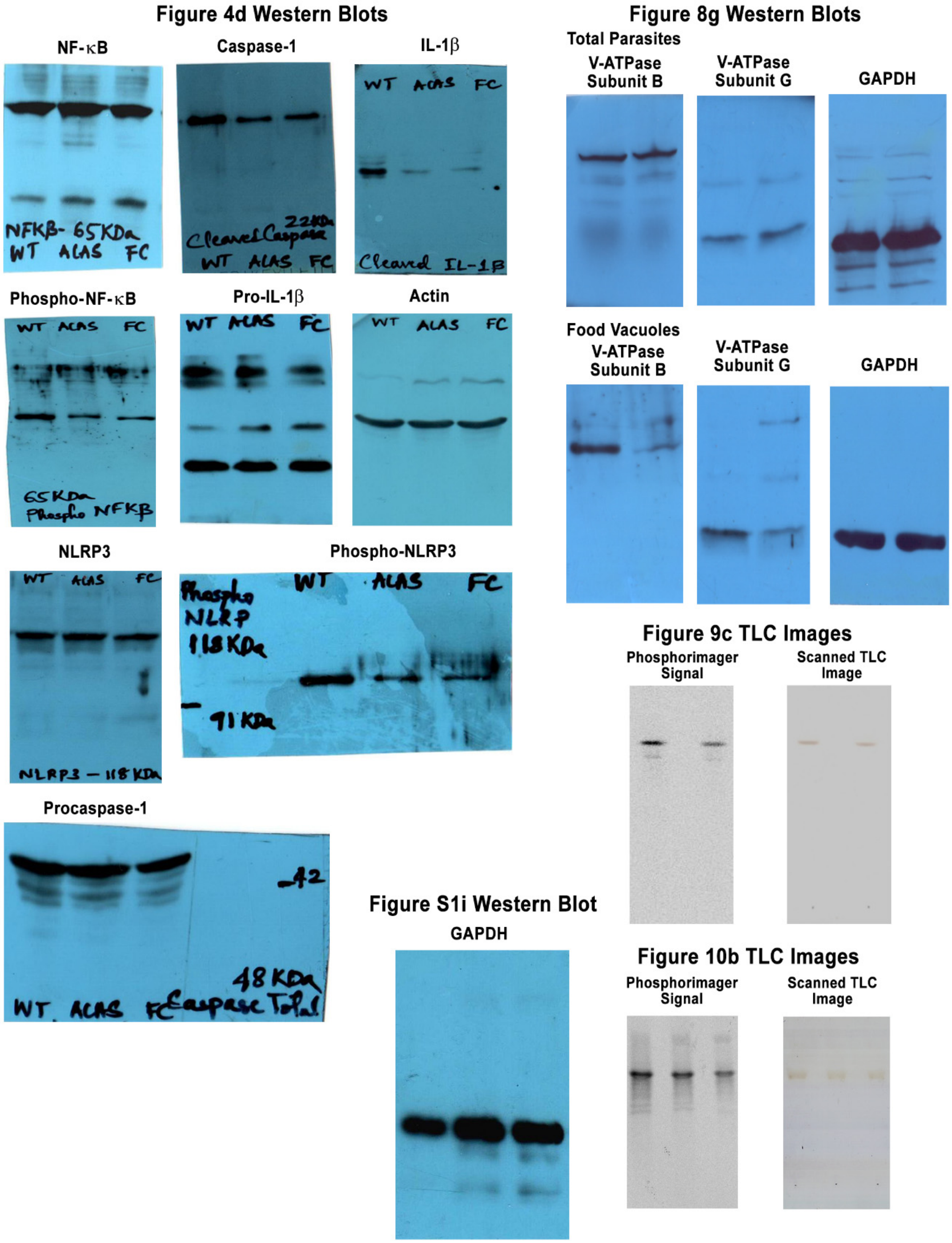

Supplementary Figure 7: Full-length images of Western blots, TLC scans and phosphorimager scans.

Supplementary Table 1. List of compounds identified in GC-MS analysis of the FAMEs prepared from *Pb* FVs. Methyl esters of oleic acid and arachidonic acid are highlighted in green. The compounds are listed in the order of their retention time.

S.No	Compound Name	Retention Time (min)	Area
1	11-Methyldodecanol	4.217	2.69E+06
2	11-Methyldodecanol	4.347	3.65E+06
3	11-Methyldodecanol	4.486	4.59E+06
4	Nonadecane	4.832	1.13E+06
5	Ethaneperoxoic acid 1-cyano-1-[2-(2-phenyl-1,3-dioxolan-2-yl)ethyl]pentyl ester	4.975	5.77E+06
6	3-Ethyl-3-methylnonadecane	7.313	9.05 E+05
7	1s,4R,7R,11R-1,3,4,7-Tetramethyltricyclo[5.3.1.0(4,11)]undec-2-en-8-one	7.965	6.89E+06
8	Nonadecane	8.097	2.22E+06
9	Phenol, 2,4-bis(1,1-dimethylethyl)	8.674	2.37E+07
10	11- Methyldodecanol	8.828	2.35E+06
11	11- Methyldodecanol	9.078	2.47E+06
12	2-Hexyl-1-dodecanol	9.265	4.81E+06
13	11- Methyldodecanol	9.540	2.94E+06
14	11- Methyldodecanol	9.801	2.18E+06
15	Tritetracontane	13.669	1.40E+06
16	11- Methyldodecanol	14.450	2.60E+06
17	2-Hexyl-1-decanol	14.850	1.55E+06

18	11- Methyldodecanol	14.932	1.58E+06
19	2-Hexyl-1-decanol	15.092	6.14E+05
20	11- Methyldodecanol	16.066	1.65E+06
21	14-methyl-pentadecanoic acid, methyl ester	19.608	3.30E+07
22	Benzenepropanoic acid, 3,5-bis(1,1-dimethylethyl)-4-hydroxy-, methyl ester	19.711	5.08E+05
23	Tritetracontane	20.177	1.58E+06
24	Methyl 9-cis, 11-trans-octadecadienoate	23.597	2.98E+06
25	9-Octadecenoic acid(Z)-,methylester	23.758	7.82E+06
26	Methyl stearate	24.380	1.88E+07
27	Tritetracontane	25.084	1.60 E+06
28	5,8,11,14-Eicosatetraenoicacid, methyl ester, (5Z,8Z,11Z,14Z)-	27.249	4.94E+06

Supplementary Table 2: Details of the primers used for qPCR and PCR analyses.

Genes	5’-3’ Sequence
Mouse GAPDH (F)	AAGGTCATCCCAGAGCTGAA
Mouse GAPDH (R)	CTGCTTCACCACCTTCTTGA
Mouse TNFα (F)	AAGCCTGTAGCCCACGTCGTA
Mouse TNFα (R)	GGCACCACTAGTTGGTTGTCTTTG
Mouse IFNγ (F)	TCAAGTGGCATAGATGTGGAAGAA
Mouse IFNγ (R)	TGGCTCTGCAGGATTTTCATG
Mouse CXCL9 (F)	GCCATGAAGTCCGCTGTTCT
Mouse CXCL9 (R)	GGGTTCCTCGAACTCCACACT
Mouse CXCL10 (F)	GACGGTCCGCTGCAACTG
Mouse CXCL10 (R)	GCTTCCCTATGGCCCTCATT
Mouse CCL2 (F)	GTCACCTGCTGCTACTCATTC
Mouse CCL2 (R)	GCTTGAGGTGGTTGTGGAAAA
Mouse CCL5 (F)	CATCCTCACTGCAGCCGCC
Mouse CCL5 (R)	CCAAGCTGGCTAGGACTAGAG
Mouse CCL19 (F)	ATGTGAATCACTCTGGCCCAGGAA
Mouse CCL19 (R)	AAGCGGCTTTATTGGAAGCTCTGC
Mouse CCL20 (F)	CGACTGTTGCCTCTCGTACA
Mouse CCL20 (R)	GAGGAGGTTCACAGCCCTTT
Mouse CCL21 (F)	TGAGCTATGTGCAAACCCTGAGGA
Mouse CCL21 (R)	TGAGGGCTGTGTCTGTTCAGTTCT
Mouse CXCR3 (F)	AATGCCACCCATTGCCAGTAC
Mouse CXCR3 (R)	AGCAGTAGGCCATGACCAGAAG

Mouse CCR7 (F)	TCATTGCCGTGGTGGTAGTCTTCA
Mouse CCR7 (R)	ATGTTGAGCTGCTTGCTGGTTTCG
Mouse Perforin (F)	CACAGTAGAGTGTCCGATGTA
Mouse Perforin (R)	CTTGGTTCCCGAAGAGCAGAT
Mouse Granzyme B (F)	CCTCCTGCTACTGCTGAC
Mouse Granzyme B (R)	GTCAGCACAAAGTCCTCTC
Mouse ICAM-1 (F)	GCCTCCGGACTTTCGATCTT
Mouse ICAM-1 (R)	GTCAGGGGTGTCGAGCTTTG
Mouse P-selectin (F)	GTCCACGGAGAGTTTGGTGT
Mouse P-selectin (R)	AAGTGGTGTTCGGACCAAAG
Mouse HO-1 (F)	GAGCCTGAATCGAGCAGAAC
Mouse HO-1 (R)	CCTTCAAGGCCTCAGACAAA
Mouse HO-2 (F)	TGGGTTGCCCTCTTAACAAA
Mouse HO-2 (R)	GTTTGTGCTGCCCTCACTCT
*Pb*GAPDH (F)	ATGGCAATAACAAAAGTCGGAATTAATGG
*Pb*GAPDH (R)	TTAATTTTTGGTGATGTGGATAGCCAAATC
*Pb*GAPDH (qPCR F)	AATTAAAGAAGCATCTGAGGGTCCAC
*Pb*GAPDH (qPCR R)	TTGAATATCCCCATTCATTGTCATACC
*Pb*VATPaseSubA (qPCR F)	GGGAATAGTATATAAGGTTGCGGGG
*Pb*VATPaseSubA (qPCR R)	CCTGTTTTAGTTACAGGATCTCCTAC
*Pb*VATPaseSubB (qPCR F)	CGAGTAAATGCGTTGGCGGC
*Pb*VATPaseSubB (qPCR R)	CCACACACTTCTAATATTTGTCCTTGTCG
*Pb*VATPaseSubG (qPCR F)	GGCACAAAGCAAAGGATCCAACG
*Pb*VATPaseSubG (qPCR R)	GCTCAATTTTAGTCACAATCTCATCTTC

*Pb*Berghepain-2 (qPCR F)	GCCATCATATTAGACCCGAGGAGG
*Pb*Berghepain-2 (qPCR R)	GTCATCATTCGAATAATTTTCAACATTTCC
*Pb*SMAC (qPCR F)	CCCTCTGAAGAGTTCAATTTTTATAATAGTACG
*Pb*SMAC (qPCR R)	TTATATGGAAGTGAAATAAGCGAGTAC
*Pb*MAHRP1a (qPCR F)	GTGTTAAAATAACACTTTTCTTTGTAAACC
*Pb*MAHRP1a (qPCR R)	TCAGTTAGTTCTATGGGTAGTAGTGCCTC
*Pb*SBP1 (qPCR F)	CACATTTTACCTTTCTTTATTTTTTTATTAGG
*Pb*SBP1 (qPCR R)	TCATTTTTTTTTAAATTTCTTTTCGGTCTTATTTGCC
*Pb*ALAS (F)	ATGAGAAAGAAAAAAGCATTAAAGGTGAGTC
*Pb*ALAS (R)	TTAAAGCTTCATTTCGATTTTGTTTTTTTTGTTG
*Pb*FC (F)	ATGGATATAGACGATTTCTTAAAATGTAACAATTTAAAC
*Pb*FC (R)	GCAATCTAGATTACCAGCCACTTAGATTTTTTTCAATAAT
*Pb*Control Integration (F)	TAGTAGTGAATTAATAATTTTTTGTATTTTATC
*Pb*ALAS Integration (F)	GCATGCCACACCAATACATGCTAAACA
*Pb*FC Integration (F)	GCAACTTAAGTGGAGCATTTTCATGCTCATAAATATATTC
*Pb*FC Internal (F)	GAAACCTTAGATGACATTGAAATTACTTATAAAC
GFP (R)	ACCTTCACCCTCTCCACTGAC
L665	GTTGAAAAATTAAAAAAAAAC
L740	CTAAGGTACGCATATCATGG
