# Supplementary material for: Malaria parasite heme biosynthesis promotes and griseofulvin protects against cerebral malaria in mice
Source: Nat Commun. 2022 Jul 12;13:4028. doi: 10.1038/s41467-022-31431-z (PMC9276668; doi:10.1038/s41467-022-31431-z)
Supplement: Supplementary file 2 — Description of Additional Supplementary Files [file 41467_2022_31431_MOESM2_ESM.pdf]

Description of Additional Supplementary Files

Supplementary Data 1: *Pb*WT FV proteome and gene ontology clustering. Proteins that are identified only in WT FVs, but not in FCKO FVs are highlighted in green. The entire peptide summary along with the identified sequences is also provided.

Supplementary Data 2: *Pb*FCKO FV proteome and gene ontology clustering. Proteins that are identified only in FCKO FVs, but not in WT FVs are highlighted in blue. The entire peptide summary along with the identified sequences is also provided.

Supplementary Data 3: Reporter ion intensities of Hb α and β chains. The reporter ion intensities obtained for Hb α and β chains from two sets of iTRAQ reactions performed with WT and FCKO FV protein preparations are highlighted in green. Reaction 1 and 2 were performed with 50 μg and 100 μg of total protein, respectively. The reporter ion intensities were normalized with respect to the internal control labelled with isobaric tag 114.

Supplementary Movie 1: Live imaging of hemozoin dynamics in *Pb*WT parasite.

Supplementary Movie 2: Live imaging of hemozoin dynamics in *Pb*ALASKO parasite.

Supplementary Movie 3: Live imaging of hemozoin dynamics in *Pb*FCKO parasite.
