# Supplementary material for: Malaria parasite heme biosynthesis promotes and griseofulvin protects against cerebral malaria in mice
Source: Nat Commun. 2022 Jul 12;13:4028. doi: 10.1038/s41467-022-31431-z (PMC9276668; doi:10.1038/s41467-022-31431-z)
Supplement: Supplementary file 9 — Reporting Summary [file 41467_2022_31431_MOESM9_ESM.pdf]

nature portfolio

Corresponding author(s): Viswanathan Arun Nagaraj

Last updated by author(s): May 17, 2022

Reporting Summary

Nature Portfolio wishes to improve the reproducibility of the work that we publish. This form provides structure for consistency and transparency in reporting. For further information on Nature Portfolio policies, see our Editorial Policies and the Editorial Policy Checklist.

Statistics

For all statistical analyses, confirm that the following items are present in the figure legend, table legend, main text, or Methods section.

n/a	Confirmed	
☐	☒	The exact sample size (*n*) for each experimental group/condition, given as a discrete number and unit of measurement
☐	☒	A statement on whether measurements were taken from distinct samples or whether the same sample was measured repeatedly
☐	☒	The statistical test(s) used AND whether they are one- or two-sided<br>*Only common tests should be described solely by name; describe more complex techniques in the Methods section.*
☐	☒	A description of all covariates tested
☐	☒	A description of any assumptions or corrections, such as tests of normality and adjustment for multiple comparisons
☐	☒	A full description of the statistical parameters including central tendency (e.g. means) or other basic estimates (e.g. regression coefficient) AND variation (e.g. standard deviation) or associated estimates of uncertainty (e.g. confidence intervals)
☐	☒	For null hypothesis testing, the test statistic (e.g. *F*, *t*, *r*) with confidence intervals, effect sizes, degrees of freedom and *P* value noted<br>*Give P values as exact values whenever suitable.*
☒	☐	For Bayesian analysis, information on the choice of priors and Markov chain Monte Carlo settings
☒	☐	For hierarchical and complex designs, identification of the appropriate level for tests and full reporting of outcomes
☒	☐	Estimates of effect sizes (e.g. Cohen's *d*, Pearson's *r*), indicating how they were calculated

Our web collection on statistics for biologists contains articles on many of the points above.

Software and code

Policy information about availability of computer code

Data collection	No software was used.
Data analysis	All the graphs were plotted using GraphPad Prism Version 7.00 software and the statistical analyses were carried out using unpaired Welch's t-test (two-sided), two-way ANOVA and log-rank (Mantel-Cox) test. For two-way ANOVA, Tukey test was performed and the multiple comparisons were corrected using statistical hypothesis testing. The non-linear regression fit was carried out for inhibitor versus response curve and the R-squared value was determined using GraphPad Prism Version 7.00 software. Flow cytometry data were analysed using FlowJo™ v10.6.1. The video files were processed using VSDC video editor v6.4.2.108 and VideoPad v4.30 NCH softwares. The fluorescent signal intensities were quantified using ImageJ software v1.52a. For GCMS analysis, NIST library search was performed to identify the compounds using Agilent Workstation Software v7.0.1. Proteomics mass spectrometry data acquisition was achieved using Analyst TF1.7.1. software. MS/MS data were annotated using Paragon algorithm (ProteinPilot Software Version 5.0.2, SCIEX).

For manuscripts utilizing custom algorithms or software that are central to the research but not yet described in published literature, software must be made available to editors and reviewers. We strongly encourage code deposition in a community repository (e.g. GitHub). See the Nature Portfolio guidelines for submitting code & software for further information.

Data

Policy information about availability of data

All manuscripts must include a data availability statement. This statement should provide the following information, where applicable:

- Accession codes, unique identifiers, or web links for publicly available datasets
- A description of any restrictions on data availability
- For clinical datasets or third party data, please ensure that the statement adheres to our policy

Source data are provided with this paper. The data that support the findings of this study are also available in figshare. doi: 10.6084/m9.figshare.19354475. For proteomics analyses, reference proteomes of Plasmodium berghei (UP000074855 (https://www.uniprot.org/uniprot/?query=plasmodium%20berghei&fil=proteome%3AUP000074855+AND+organism%3A%22Plasmodium+berghei+%28strain+Anka%29+%5B5823%5D%22&sort=score), Taxonomy 5823; UP000219974 (https://www.uniprot.org/uniprot/?query=plasmodium%20berghei&fil=proteome%3AUP000219974+AND+organism%3A%22Plasmodium+berghei+%5B5821%5D%22&sort=score), Taxonomy 5821) and Mus musculus (UP000000589 (https://www.uniprot.org/uniprot/?query=mus%20musculus&fil=proteome%3AUP000000589+AND+organism%3A%22Mus+musculus+%28Mouse%29+%5B10090%5D%22&sort=score), Taxonomy 10090) were accessed through Uniprot (https://www.uniprot.org/) website. The mass spectrometry proteomics data of LC-MS/MS and iTRAQ have been deposited to the ProteomeXchange Consortium via the PRIDE partner repository with the dataset identifiers PXD031736 (http://www.ebi.ac.uk/pride/archive/projects/PXD031736) and PXD031738 (http://www.ebi.ac.uk/pride/archive/projects/PXD031738), respectively.

Field-specific reporting

Please select the one below that is the best fit for your research. If you are not sure, read the appropriate sections before making your selection.

☒ Life sciences ☐ Behavioural & social sciences ☐ Ecological, evolutionary & environmental sciences

For a reference copy of the document with all sections, see nature.com/documents/nr-reporting-summary-flat.pdf

Life sciences study design

All studies must disclose on these points even when the disclosure is negative.

Sample size	There is a vast literature describing cerebral malaria experiments in mice including the assessment of inflammation parameters, cerebral pathogenesis, protection phenotype etc. Here is the list of some of the relevant references provided in the study: Pamplona et al., Nat Med 2007 (PMID: 17496899); Bapista et al., Infect Immun 2010 (PMID: 20605973); Ferreira et al., Cell 2011 (PMID: 21529713); Lin et al., J Exp Med 2015(PMID: 25941254); Strangward et al., PLoS Pathog 2017(PMID: 28273147); Niz et al., Nat Commun 2017 (PMID: 27225796); Raulf et al., Cell Rep 2019 (PMID: 31269448). Therefore, the number of mice required for our experiments were decided based on the available literature. In addition, we determined the number of mice required for each experiment by performing power analysis using G*Power 3.1.9.7 with the mean and standard deviation values obtained from pilot studies for the respective parameters. The effect sizes were determined and an alpha value of 0.05 and a power of 0.80 were used to determine the group size. The cerebral malaria mortality in wildtype parasite-infected mice was adjusted by including 10% extra mice. For experiments that do not involve mice such as quantification of fluorescence intensity, measurement of radiolabelling, effect of griseofulvin on Pf3D7 cultures etc., at least three independent experiments were carried out - a practice that is common across the research publications describing similar experiments. For experiments carried out with food vacuoles, three different preparations from independent mice infected with the respective parasites were used - once again, a common practice across the research publications (Palacpac et al., J Cell Sci 2004 (PMID: 15020675); Gratraud et al., PLoS One 2009 (PMID: 19707292); Nagaraj et al., Plos Pathog 2013 (PMID: 23935500); El-Assaad et al., Infect Immun 2013 (PMID: 23940206); Harding et al., Nat Commun 2020). For clinical isolate experiments, five different isolates were used to examine the effect observed for Pf3D7. All these were sufficient to perform statistical comparison, and ensure statistical significance and reproducibility of the observations made.
Data exclusions	No data were excluded from the analysis.
Replication	The reproducibility of the experimental results was verified by (i) performing the experiments in multiple independent batches (ii) examining the results with independent mice and (iii) confirming the findings independently by at least two different authors. The exact details of the number of mice, experiments etc are provided in the respective figure legends. All attempts at replication were successful.
Randomization	Random
Blinding	The important findings such as cerebral malaria protection in the heme pathway knockout parasites, decreased hemozoin formation in the knockout parasites, and griseofulvin protection were examined by blinding the parasite strains or samples at least once to confirm the results. However, subsequent blinding for every single experiment was difficult because different parasite strains have to be injected in different numbers (10^5 for wild type and 10^7 for knockout parasite). Nevertheless, the infection experiments were initiated by one author whereas, the data were acquired by another author to whom the groups were not disclosed. In addition, wildtype/ knockout phenotype of the parasites in infected mice displaying ECM/non-ECM features was routinely confirmed in all the experiments by performing PCR during which the DNA samples were blinded and PCR analyses were carried out by an author who was not aware of the experiment results.

Reporting for specific materials, systems and methods

We require information from authors about some types of materials, experimental systems and methods used in many studies. Here, indicate whether each material, system or method listed is relevant to your study. If you are not sure if a list item applies to your research, read the appropriate section before selecting a response.

Materials & experimental systems

n/a	Involved in the study
☐	☒ Antibodies
☐	☒ Eukaryotic cell lines
☒	☐ Palaeontology and archaeology
☐	☒ Animals and other organisms
☐	☒ Human research participants
☒	☐ Clinical data
☒	☐ Dual use research of concern

Methods

n/a	Involved in the study
☒	☐ ChIP-seq
☐	☒ Flow cytometry
☒	☐ MRI-based neuroimaging

Antibodies

Antibodies used

1) anti-CD31 mouse monoclonal antibody conjugated with Alexa Fluor 594 (SantaCruz, sc-376764; 1:200 dilution; Lot number: D2518)
2) anti-PbGAPDH rabbit polyclonal serum (1:100 dilution for immunohistochemistry and 1:500 for Western)
3) anti-mouse CD3 rat monoclonal antibody (Invitrogen, 14-0032-82; 1:100 dilution)
4) FITC-conjugated donkey anti-rabbit IgG (SantaCruz, sc-2090; 1:200 dilution; Lot number: 11213)
5) FITC-conjugated goat anti-rat IgG (SantaCruz, sc-2011; 1:200 dilution)
6) Anti-mouse beta-APP rabbit polyclonal antibody (Thermo Fisher Scientific, 51-2700; 1:200 dilution; Lot number: UK294411)
7) Anti-mouse CD3-FITC (clone 17A2; Thermo Fisher Scientific, 11-0032-82; 0.25 microgram / 10^6 cells in 100 microlitre volume; Lot number: 2373704)
8) Anti-mouse CD4-PE (clone RM4-5; Thermo Fisher Scientific, 12-0043-82; 0.125 microgram / 10^6 cells in 100 microlitre volume)
9) Anti-mouse CD8-PerCP-Cyanine5.5 (clone 53-6.7; Thermo Fisher Scientific, 45-0081-82; 0.25 microgram / 10^6 cells in 100 microlitre volume; Lot number: 2151510)
10) Anti-mouse CD69-Brilliant Violet 421 (clone H1.2F3; BioLegend, 104527; 0.25 microgram / 10^6 cells in 100 microlitre volume; Lot number: B246551)
11) Anti-mouse CXCR3-PE (clone CXCR3-173; Thermo Fisher Scientific, 12-1831-82; 0.25 microgram / 10^6 cells in 100 microlitre volume; Lot number: 1995421)
12) Anti-mouse Perforin-PE (clone S16009A; BioLegend, 154305; 0.50 microgram / 10^6 cells in 100 microlitre volume; Lot number: B262752)
13) Anti-mouse Granzyme B-APC (clone NGZB; Thermo Fisher Scientific, 17-8898-82; 0.125 microgram / 10^6 cells in 100 microlitre volume; Lot number: 4342183)
14) Anti-mouse IFNγ-eFluor 450 (clone XMG1.2; Thermo Fisher Scientific, 48-7311-82; 0.50 microgram / 10^6 cells in 100 microlitre volume; Lot number: 2026274)
15) Anti-mouse TNFα-eFluor 450 (clone MP6-XT22; Thermo Fisher Scientific, 48-7321-82; 0.25 microgram / 10^6 cells in 100 microlitre volume; Lot number: 1995421)
16) Anti-mouse NF-kappa B p65 (Thermo Fisher Scientific, 14-6731-81; 1:1000 dilution; Lot number: 2163864)
17) Anti-mouse Phospho-NF-kappa B p65 (Ser536) (Thermo Fisher Scientific, MA5-15160; 1:1000 dilution; Lot number: UH28295-78)
18) Anti-mouse NLRP3 (Thermo Fisher Scientific, PA5-20838; 1:1000 dilution; Lot number: VB2933205)
19) Anti-mouse Phospho-NLRP3 (Ser295) (Thermo Fisher Scientific, PA5-105071; 1:1000 dilution; Lot number: VA29276-63)
20) Anti-mouse Caspase 1 (Thermo Fisher Scientific, 14-9832-82; 1:500 dilution; Lot number: 2045370)
21) Anti-mouse Cleaved Caspase-1 (Asp296) (Cell Signaling Technology, 89332; 1:1000 dilution; Lot number: 1)
22) Anti-mouse IL-1beta (Thermo Fisher Scientific, 701304; 1:250 dilution; Lot number: 2070330)
23) Anti-mouse Cleaved IL-1beta (Cell Signaling Technology, 52718; 1:1000 dilution; Lot number: 1)
24) Anti-mouse β-Actin (Cell Signaling Technology, 3700; 1:2000 dilution)
25) PfFC polyclonal serum (1:500 dilution)
26) PbV-type H+ATPase subunits B (1:1000 dilution)
27) PbV-type H+ATPase subunits G (1:1000 dilution)
28) HRP-conjugated goat anti-mouse IgG (Abcam, ab97023; 1:250 dilution; Lot number: GR215612-4)

Validation

1) anti-CD31 mouse monoclonal antibody conjugated with Alexa Fluor 594 : https://datasheets.scbt.com/sc-376764.pdf
2) anti-PbGAPDH rabbit polyclonal serum : Present Study
3) anti-mouse CD3 rat monoclonal antibody : https://www.thermofisher.com/order/genome-database/dataSheetPdf?producttype=antibody&productsubtype=antibody_primary&productId=14-0032-82&version=223
4) FITC-conjugated donkey anti-rabbit IgG : https://datasheets.scbt.com/sc-2090.pdf
5) FITC-conjugated goat anti-rat IgG : https://datasheets.scbt.com/sc-2011.pdf
6) Anti-mouse beta-APP rabbit polyclonal antibody : https://www.thermofisher.com/order/genome-database/dataSheetPdf?producttype=antibody&productsubtype=antibody_primary&productId=51-2700&version=223
7) Anti-mouse CD3-FITC : https://www.thermofisher.com/order/genome-database/dataSheetPdf?producttype=antibody&productsubtype=antibody_primary&productId=11-0032-82&version=223
8) Anti-mouse CD4-PE : https://www.thermofisher.com/order/genome-database/dataSheetPdf?producttype=antibody&productsubtype=antibody_primary&productId=12-0042-82&version=223
9) Anti-mouse CD8-PerCP-Cyanine5.5 : https://www.thermofisher.com/order/genome-database/dataSheetPdf?

producttype=antibody&productsubtype=antibody_primary&productId=45-0081-82&version=223
10) Anti-mouse CD69-Brilliant Violet 421 : https://www.biolegend.com/de-de/products/brilliant-violet-421-anti-mouse-cd69-antibody-7358?pdf=true&displayInline=true&leftRightMargin=15&topBottomMargin=15&filename=Brilliant%20Violet%20421%E2%84%A2%20anti-mouse%20CD69%20Antibody.pdf
11) Anti-mouse CXCR3-PE : https://www.thermofisher.com/order/genome-database/dataSheetPdf?producttype=antibody&productsubtype=antibody_primary&productId=12-1831-82&version=223
12) Anti-mouse Perforin-PE : https://www.biolegend.com/it-it/products/pe-anti-mouse-perforin-antibody-15255?pdf=true&displayInline=true&leftRightMargin=15&topBottomMargin=15&filename=PE%20anti-mouse%20Perforin%20Antibody.pdf
13) Anti-mouse Granzyme B-APC : https://www.thermofisher.com/order/genome-database/dataSheetPdf?producttype=antibody&productsubtype=antibody_primary&productId=17-8898-82&version=223
14) Anti-mouse IFNγ-eFluor 450 : https://www.thermofisher.com/order/genome-database/dataSheetPdf?producttype=antibody&productsubtype=antibody_primary&productId=48-7311-82&version=223
15) Anti-mouse TNFα-eFluor 450 : https://www.thermofisher.com/order/genome-database/dataSheetPdf?producttype=antibody&productsubtype=antibody_primary&productId=48-7321-82&version=223
16) Anti-mouse NF-kappa B p65: https://www.thermofisher.com/order/genome-database/dataSheetPdf?producttype=antibody&productsubtype=antibody_primary&productId=14-6731-81&version=223
17) Anti-mouse Phospho-NF-kappa B p65 (Ser536): https://www.thermofisher.com/order/genome-database/dataSheetPdf?producttype=antibody&productsubtype=antibody_primary&productId=MA5-15160&version=223
18) Anti-mouse NLRP3: https://www.thermofisher.com/order/genome-database/dataSheetPdf?producttype=antibody&productsubtype=antibody_primary&productId=PA5-20838&version=223
19) Anti-mouse Phospho-NLRP3 (Ser295): https://www.thermofisher.com/order/genome-database/dataSheetPdf?producttype=antibody&productsubtype=antibody_primary&productId=PA5-105071&version=223
20) Anti-mouse Caspase 1: https://www.thermofisher.com/order/genome-database/dataSheetPdf?producttype=antibody&productsubtype=antibody_primary&productId=14-9832-82&version=223
21) Anti-mouse Cleaved Caspase-1 (Asp296): https://www.cellsignal.com/products/primary-antibodies/cleaved-caspase-1-asp296-e2g2i-rabbit-mab/89332
22) Anti-mouse IL-1beta: https://www.thermofisher.com/order/genome-database/dataSheetPdf?producttype=antibody&productsubtype=antibody_primary&productId=701304&version=223
23) Anti-mouse Cleaved IL-1beta: https://www.cellsignal.com/products/primary-antibodies/cleaved-il-1b-asp117-antibody-mouse-specific/52718
24) Anti-mouse β-Actin: https://www.cellsignal.com/products/primary-antibodies/b-actin-8h10d10-mouse-mab/3700
25) PfFC polyclonal serum (1:500 dilution) (Mitochondrial localization of functional ferrochelatase from Plasmodium falciparum; Molecular and Biochemical Parasitology 168(1):109-12; DOI:10.1016/j.molbiopara.2009.05.008 and Present Study)
26) PbV-type H+ATPase subunits B: Present Study
27) PbV-type H+ATPase subunits G: Present Study
28) HRP-conjugated goat anti-mouse IgG: https://www.abcam.com/goat-mouse-igg-hl-hrp-ab97023.html

Eukaryotic cell lines

Policy information about cell lines

Cell line source(s)	Pb ANKA (MRA-311) deposited by Thomas F. McCutchan, mouse brain endothelial cell line b.End5 (Sigma-Aldrich, 96091930), PfCam clinical isolate (IPC 5202) (MRA-1240) deposited by Didier Menard, strain K1 (MRA-159) deposited by Dennis E. Kyle
Authentication	1) Pb ANKA (MRA-311) - Authenticated strain from Malaria Research and Reference Reagent Resource Center (MR4), ATCC, Manassas, Virginia. We further authenticated it by sequencing at least 10 genes from this strain including GAPDH, ALAS, FC, 18srRNA. In addition, cerebral malaria phenotype was confirmed.<br>2) Mouse brain endothelial cell line b.End5 (Sigma-Aldrich, 96091930) - Authenticated by morphology.<br>3) PfCam clinical isolate (IPC 5202) (MRA-1240) - Authenticated strain from Malaria Research and Reference Reagent Resource Center (MR4), ATCC, Manassas, Virginia. We further authenticated it by verifying artemisinin resistance.<br>4) Strain K1 (MRA-159) - Authenticated strain from Malaria Research and Reference Reagent Resource Center (MR4), ATCC, Manassas, Virginia. We further authenticated it by verifying chloroquine resistance.
Mycoplasma contamination	Negative
Commonly misidentified lines (See ICLAC register)	No commonly misidentified cell lines were used.

Animals and other organisms

Policy information about studies involving animals; ARRIVE guidelines recommended for reporting animal research

Laboratory animals	C57BL/6 male and female mice; Balb/c male mice; 7-8 weeks old; Breeding and maintenance of mice were performed at the animal house facility of Institute of Life Sciences, Bhubaneswar, under standard conditions of 25 +/- 3ºC temperature, 40-50% relative humidity, and 12 h light / 12 h dark cycle. Parasite strains procured from Malaria Research and Reference Reagent Resource Center (MR4), ATCC Manassas Virginia: Pb ANKA (MRA-311) deposited by Thomas F. McCutchan, PfCam clinical isolate (IPC 5202 (MRA-1240) deposited by Didier Menard, and strain K1 (MRA-159) deposited by Dennis E. Kyle.
Wild animals	No wild animals were used in the study.
Field-collected samples	No field collected samples were used in the study.

Ethics oversight	All the studies involving mice were carried out with the approval of Institutional Animal Ethics Committee (ILS/IAEC-57-AH/JAN-16), Institute of Life Sciences, Bhubaneswar, according to the national guidelines framed by "The Committee for the Purpose of Control and Supervision of Experiments on Animals (CPCSEA)".

Note that full information on the approval of the study protocol must also be provided in the manuscript.

Human research participants

Policy information about studies involving human research participants

Population characteristics	This work does not include any data from human research participants. We have used three clinical isolates that were earlier collected from malaria-infected patients and used them to perform in vitro experiments. The details are given in methods section. Pfl-1: Clinical isolate from Male, 17 years old. Pfl-2: Clinical isolate from Male, 37 years old; Pfl-3: Clinical isolate from Female, 25 years old. Further, covariate-relevant population characteristics do not influence the results of this study since the cryopreserved clinical isolates were used only for in vitro culture treatment studies.
Recruitment	Patients were recruited based on the confirmation for P. falciparum infections by light microscopy, rapid diagnostic test and PCR. We have excluded children below 10 years of age and adults above 60 years, mixed infection with another Plasmodium species, patients with human immunodeficiency virus (HIV) infection or other immunosuppressive disorders, hepatitis, evidence of clinically significant cardiovascular, pulmonary, metabolic, gastrointestinal, neurological, endocrine diseases, malignant disorders and pregnancy. Blood samples were collected and cryopreserved from patients aged between 10-60 years (male and female) who visited Ispat General Hospital with febrile episodes and confirmed for malaria by RDT and/or light microscopy. PCR analysis was also carried out to confirm P. falciparum mono-infections.
Ethics oversight	The clinical samples were collected with the approval of Institutional Ethics Committee (IEB) / Institutional Review Board (IRB) (94/HEC/19), Institute of Life Sciences, Bhubaneswar.

Note that full information on the approval of the study protocol must also be provided in the manuscript.

Flow Cytometry

Plots

Confirm that:

- [x] The axis labels state the marker and fluorochrome used (e.g. CD4-FITC).
- [x] The axis scales are clearly visible. Include numbers along axes only for bottom left plot of group (a 'group' is an analysis of identical markers).
- [x] All plots are contour plots with outliers or pseudocolor plots.
- [x] A numerical value for number of cells or percentage (with statistics) is provided.

Methodology

Sample preparation	Mice were anesthetized and transcardially perfused with PBS, and the brain samples were dissected out and harvested in RPMI-1640 medium containing 10% FBS. For preparing single cell suspensions, the samples were minced and digested in RPMI-1640 medium containing 0.05% Collagenase D and 2U/ml DNase I for 30 minutes at room temperature, and passed through 70 micron nylon cell strainer, followed by 5 minutes of incubation on ice. Brain homogenates were then overlaid on 30% Percoll cushion and centrifuged at 400 g for 20 minutes at room temperature. The leukocyte pellets obtained were resuspended in 1ml of RBC lysis buffer (155 mM NH4Cl, 10 mM NaHCO3 and 0.1 mM EDTA; pH 7.3) and incubated on ice for 5 minutes to remove any residual RBCs. The pellets were then washed with RPMI-1640, counted and stained for various markers.
Instrument	BD LSRFortessa; CytoFLEX S
Software	FlowJo™ v10.6.1
Cell population abundance	These studies were performed with leukocytes isolated from P. berghei infected mice with standard protocol that has been followed for experimental cerebral malaria. The average total yield of cells in the single cell suspensions prepared from one cerebral malaria WT-infected mouse brain was around 10^6 of heterogenous nature with ~15% of the cells showing CD3 positivity.
Gating strategy	The total events acquired from the side scatter and forward scatter were gated for CD3 positivity. Unstained controls for the respective fluorophores were used in all the experiments to eliminate the background fluorescence and negative populations. This was performed for all the markers that were tested. Single fluorochrome-stained cells were used to compensate for the spectral overlap. The fluorophores used for different markers are provided in the Methods section.

- [x] Tick this box to confirm that a figure exemplifying the gating strategy is provided in the Supplementary Information.
