# Supplementary material for: Malaria parasite heme biosynthesis promotes and griseofulvin protects against cerebral malaria in mice
Source: Nat Commun. 2022 Jul 12;13:4028. doi: 10.1038/s41467-022-31431-z (PMC9276668; doi:10.1038/s41467-022-31431-z)
Supplement: Supplementary file 10 — Source Data [file 41467_2022_31431_MOESM10_ESM.zip › Source Data/GCMS for Lipid Analysis/Compound Report.pdf]

Sample ID:	A1	Operator:	
Instrument ID:	MS Instrument #1	Last Calibration:	None
Measurement Type:	Area	Calibration Type:	External Standard
Acquisition Date:	10/20/2020 1:52 PM	Data File:	d:\201020_ils\a1.sms
Calculation Date:	10/20/2020 5:01 PM	Method:	d:\ms\method\fa.mth
Sample Type:	Analysis		
Inj. Sample Notes:	None		

Compound Information

Peak Name: 11-Methyldodecanol
Result Index: 1 Peak Number: 1 CAS Number: 85763-57-1 TIC

Identification

Parameter	Specification	Actual	Status
Search Type	Library Search		
Retention Time		4.217 min.	
1st Match Library		mainlib	
1st Match Entry No.		31330	
2nd Match Library		mainlib	
2nd Match Entry No.		32127	
3rd Match Library		replib	
3rd Match Entry No.		963	
Forward Match	N-F >= 500	827	Pass
Reverse Match		827	

Integration and Quantitation

Parameter	Specification	Actual	Status
Quan Ions	RIC		
RF Used	1.000		
Area	>=5000	2.686e+6	Pass
Height		1.313e+6	
Amount		2685699 Counts	

Match Types: N-F : Normal-Forward

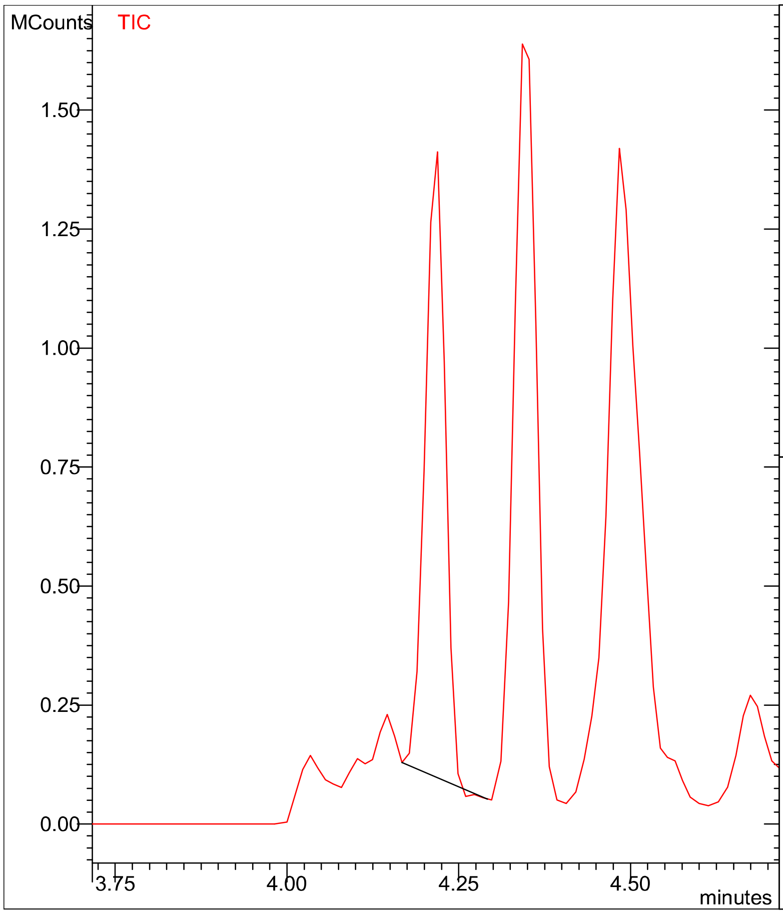

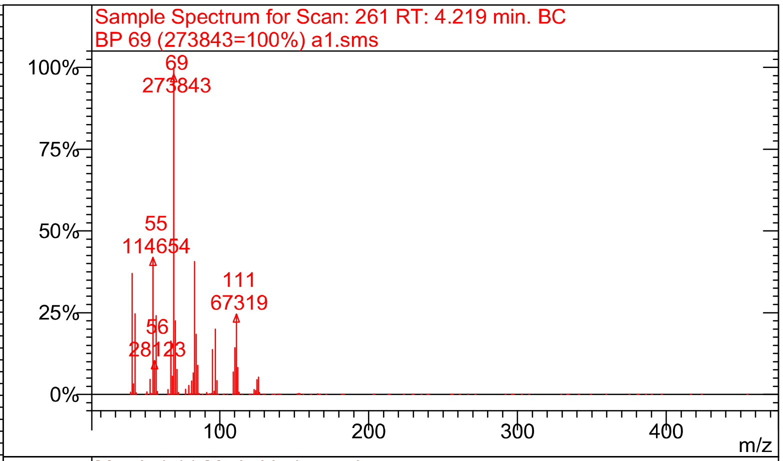

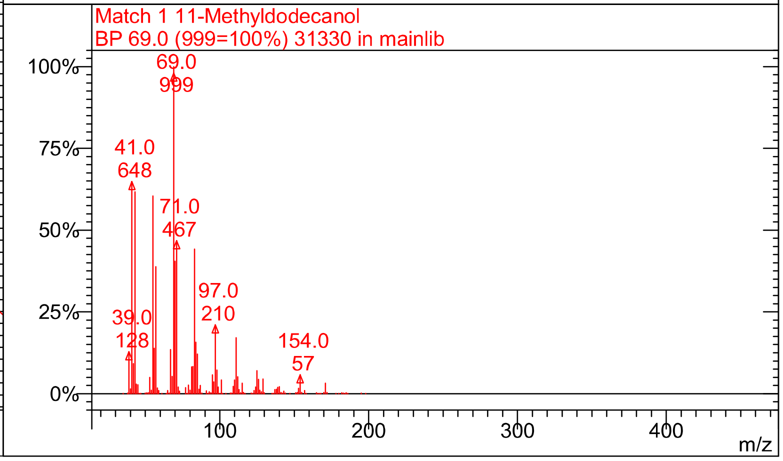

Seg 2, <No Description>, Time: 4.00-30.00

Sample ID:	A1	Operator:	
Instrument ID:	MS Instrument #1	Last Calibration:	None
Measurement Type:	Area	Calibration Type:	External Standard
Acquisition Date:	10/20/2020 1:52 PM	Data File:	d:\201020_ils\a1.sms
Calculation Date:	10/20/2020 5:01 PM	Method:	d:\ms\method\fa.mth
Sample Type:	Analysis		
Inj. Sample Notes:	None		

Compound Information

Peak Name:	11-Methyldodecanol		
Result Index:	2	Peak Number: 2	CAS Number: 85763-57-1	TIC

Identification

Parameter	Specification	Actual	Status
Search Type	Library Search		
Retention Time		4.347 min.	
1st Match Library		mainlib	
1st Match Entry No.		31330	
2nd Match Library		mainlib	
2nd Match Entry No.		32127	
3rd Match Library		replib	
3rd Match Entry No.		963	
Forward Match	N-F >= 500	816	Pass
Reverse Match		816	

Integration and Quantitation

Parameter	Specification	Actual	Status
Quan Ions	RIC		
RF Used	1.000		
Area	>=5000	3.650e+6	Pass
Height		1.589e+6	
Amount		3650015 Counts	

Match Types: N-F : Normal-Forward

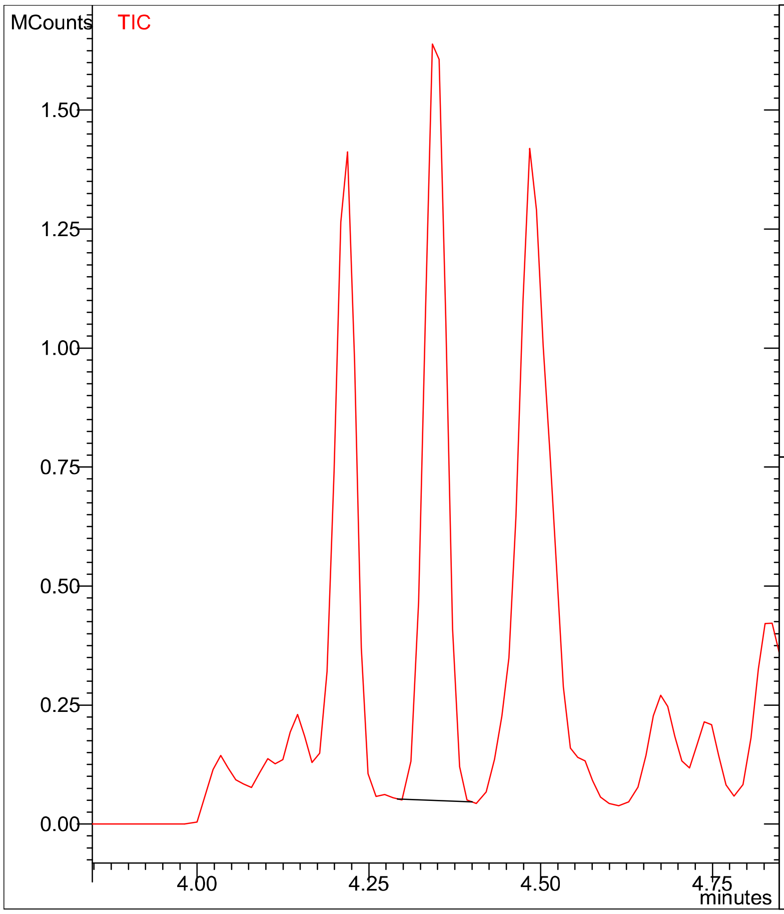

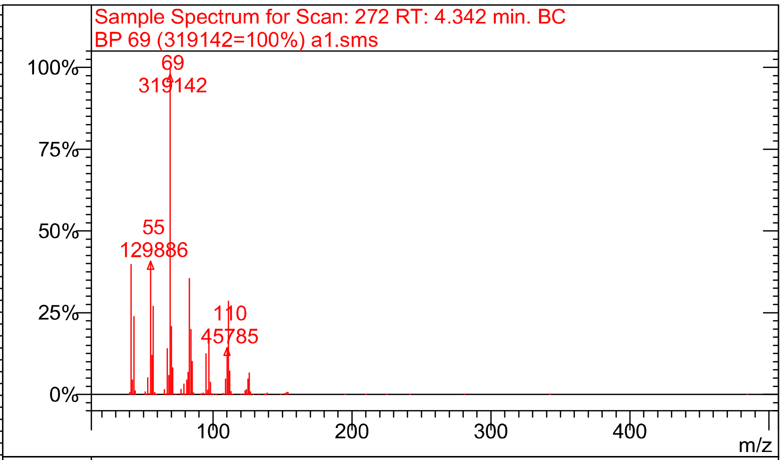

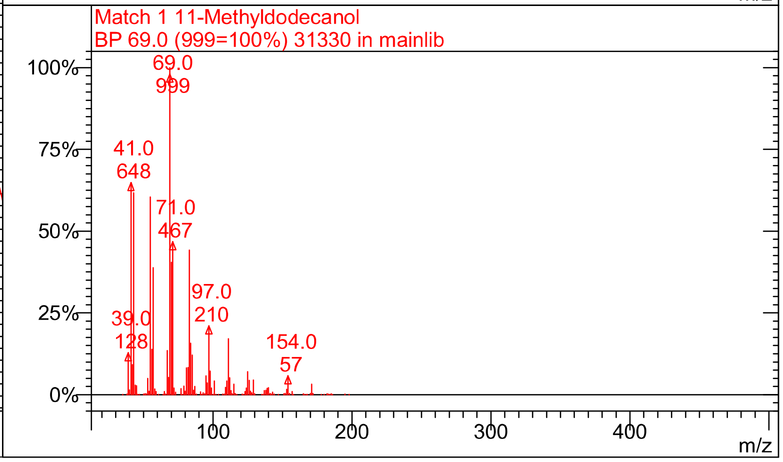

Seg 2, <No Description>, Time: 4.00-30.00

Sample ID:	A1	Operator:	
Instrument ID:	MS Instrument #1	Last Calibration:	None
Measurement Type:	Area	Calibration Type:	External Standard
Acquisition Date:	10/20/2020 1:52 PM	Data File:	d:\201020_ils\a1.sms
Calculation Date:	10/20/2020 5:01 PM	Method:	d:\ms\method\fa.mth
Sample Type:	Analysis		
Inj. Sample Notes:	None		

Compound Information

Peak Name: 11-Methyldodecanol

Result Index: 3 Peak Number: 3 CAS Number: 85763-57-1 TIC

Identification

Parameter	Specification	Actual	Status
Search Type	Library Search		
Retention Time		4.486 min.	
1st Match Library		mainlib	
1st Match Entry No.		31330	
2nd Match Library		mainlib	
2nd Match Entry No.		32127	
3rd Match Library		mainlib	
3rd Match Entry No.		23390	
Forward Match	N-F >= 500	838	Pass
Reverse Match		838	

Integration and Quantitation

Parameter	Specification	Actual	Status
Quan Ions	RIC		
RF Used	1.000		
Area	>=5000	4.594e+6	Pass
Height		1.375e+6	
Amount		4593788 Counts	

Match Types: N-F : Normal-Forward

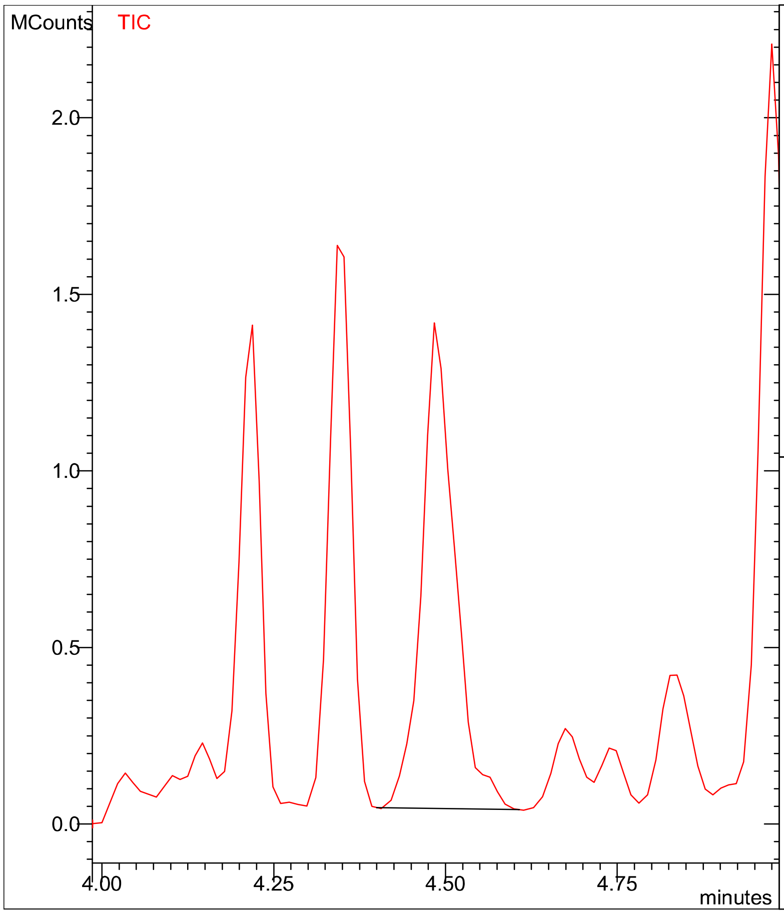

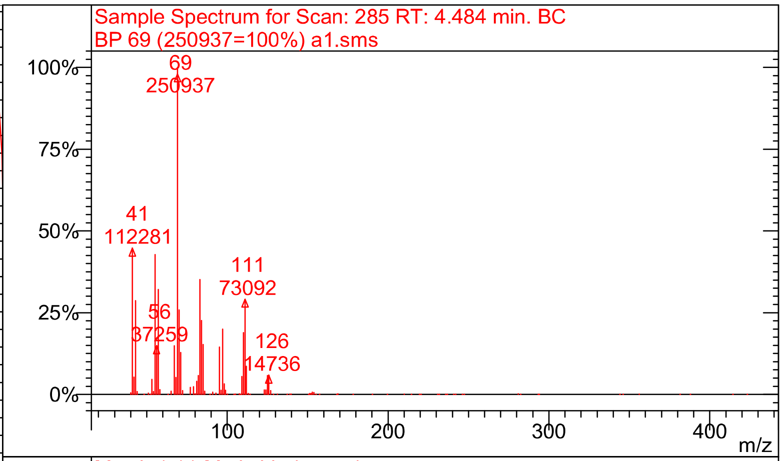

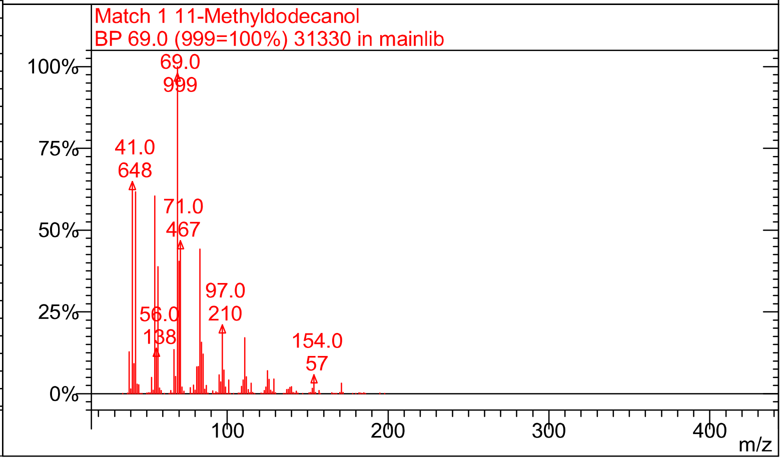

Seg 2, <No Description>, Time: 4.00-30.00

Sample ID:	A1	Operator:	
Instrument ID:	MS Instrument #1	Last Calibration:	None
Measurement Type:	Area	Calibration Type:	External Standard
Acquisition Date:	10/20/2020 1:52 PM	Data File:	d:\201020_ils\a1.sms
Calculation Date:	10/20/2020 5:01 PM	Method:	d:\ms\method\fa.mth
Sample Type:	Analysis		
Inj. Sample Notes:	None		

Compound Information

Peak Name: Nonadecane
Result Index: 4 Peak Number: 4 CAS Number: 629-92-5 TIC

Identification

Parameter	Specification	Actual	Status
Search Type	Library Search		
Retention Time		4.832 min.	
1st Match Library		replib	
1st Match Entry No.		5836	
2nd Match Library		replib	
2nd Match Entry No.		5861	
3rd Match Library		replib	
3rd Match Entry No.		5814	
Forward Match	N-F >= 500	893	Pass
Reverse Match		893	

Integration and Quantitation

Parameter	Specification	Actual	Status
Quan Ions	RIC		
RF Used	1.000		
Area	>=5000	1.126e+6	Pass
Height		365032	
Amount		1126061 Counts	

Match Types: N-F : Normal-Forward

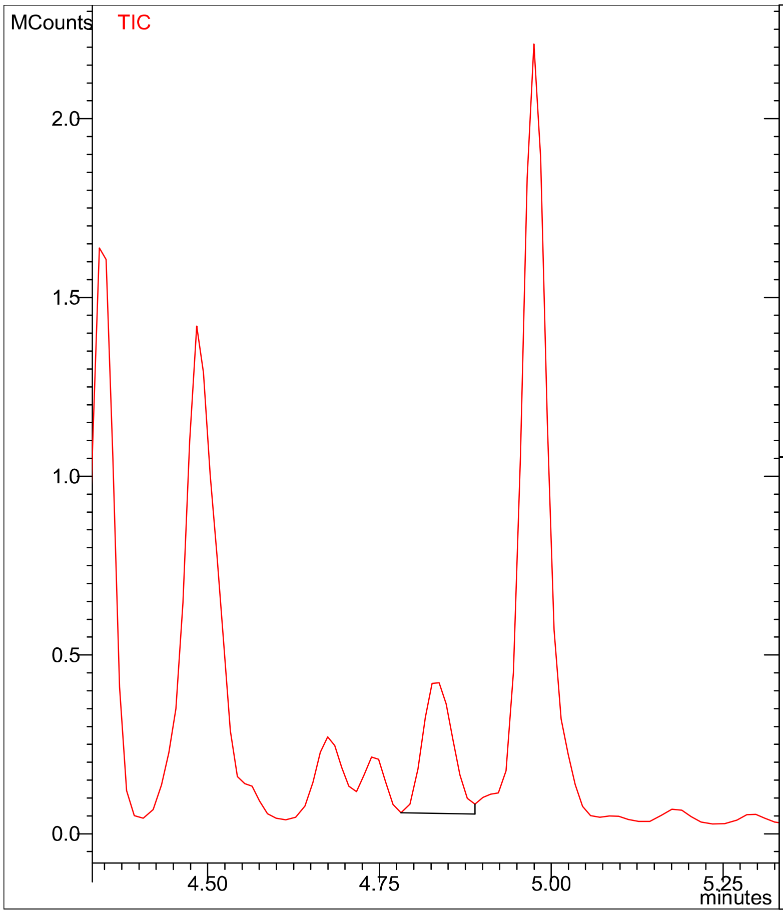

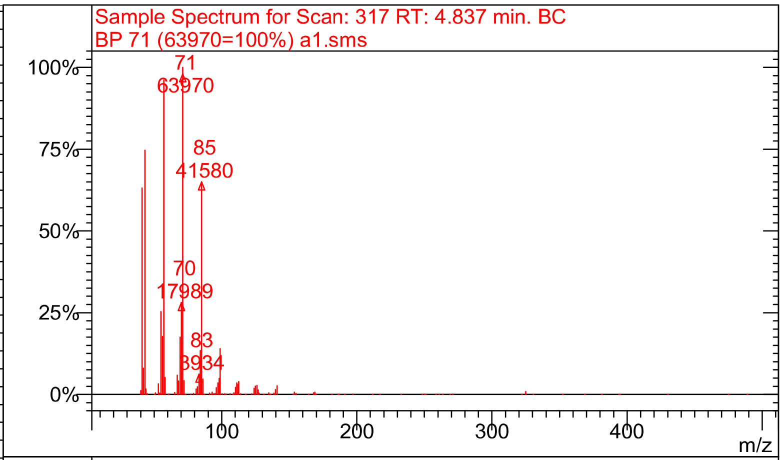

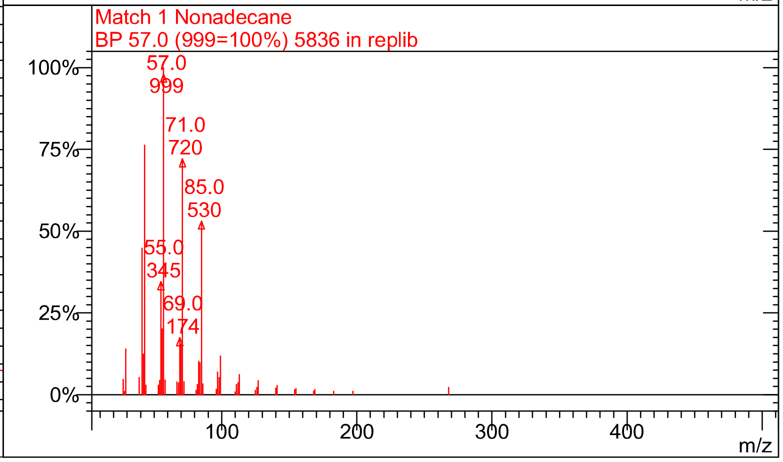

Seg 2, <No Description>, Time: 4.00-30.00

Sample ID:	A1	Operator:	
Instrument ID:	MS Instrument #1	Last Calibration:	None
Measurement Type:	Area	Calibration Type:	External Standard
Acquisition Date:	10/20/2020 1:52 PM	Data File:	d:\201020_ils\a1.sms
Calculation Date:	10/20/2020 5:01 PM	Method:	d:\ms\method\fa.mth
Sample Type:	Analysis		
Inj. Sample Notes:	None		

Compound Information

Peak Name: Ethaneperoxoic acid, 1-cyano-1-[2-(2-phe

Result Index: 5 Peak Number: 5 CAS Number: 58422-92-7 TIC

Identification

Parameter	Specification	Actual	Status
Search Type	Library Search		
Retention Time		4.975 min.	
1st Match Library		mainlib	
1st Match Entry No.		121991	
2nd Match Library		mainlib	
2nd Match Entry No.		121990	
3rd Match Library		mainlib	
3rd Match Entry No.		121886	
Forward Match	N-F >= 500	850	Pass
Reverse Match		992	

Integration and Quantitation

Parameter	Specification	Actual	Status
Quan Ions	RIC		
RF Used	1.000		
Area	>=5000	5.766e+6	Pass
Height		2.157e+6	
Amount		5766211 Counts	

Match Types: N-F : Normal-Forward

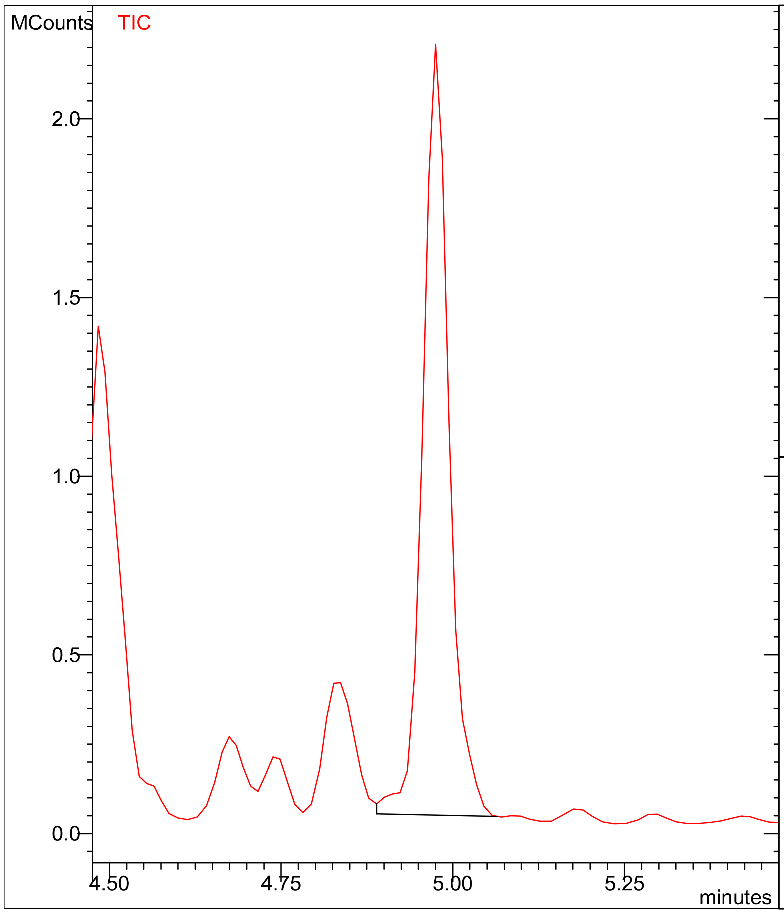

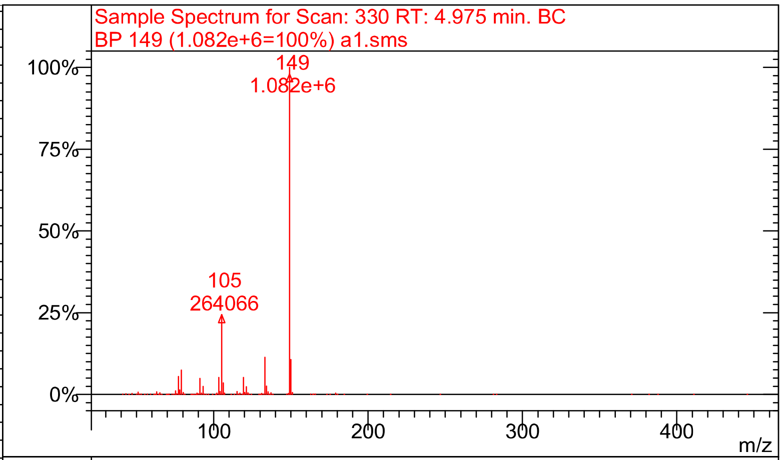

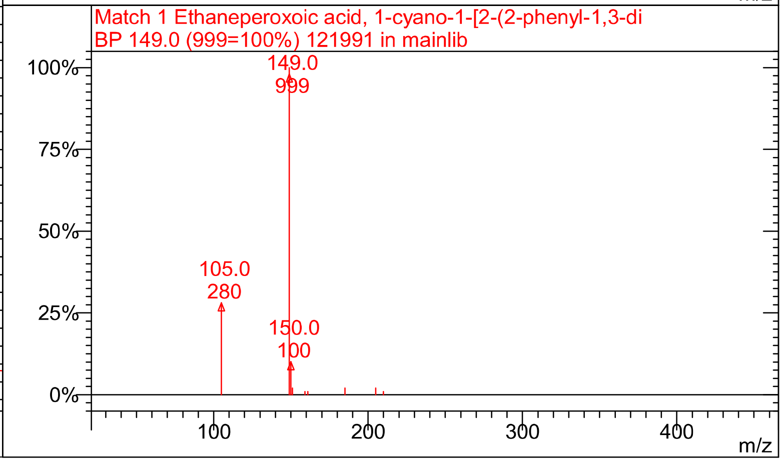

Seg 2, <No Description>, Time: 4.00-30.00

Sample ID:	A1	Operator:	
Instrument ID:	MS Instrument #1	Last Calibration:	None
Measurement Type:	Area	Calibration Type:	External Standard
Acquisition Date:	10/20/2020 1:52 PM	Data File:	d:\201020_ils\a1.sms
Calculation Date:	10/20/2020 5:01 PM	Method:	d:\ms\method\fa.mth
Sample Type:	Analysis		
Inj. Sample Notes:	None		

Compound Information

Peak Name: 3-Ethyl-3-methylnonadecane
Result Index: 6 Peak Number: 6 CAS Number: None TIC

Identification

Parameter	Specification	Actual	Status
Search Type	Library Search		
Retention Time		7.313 min.	
1st Match Library		mainlib	
1st Match Entry No.		49776	
2nd Match Library		replib	
2nd Match Entry No.		2226	
3rd Match Library		mainlib	
3rd Match Entry No.		7471	
Forward Match	N-F >= 500	660	Pass
Reverse Match		772	

Integration and Quantitation

Parameter	Specification	Actual	Status
Quan Ions	RIC		
RF Used	1.000		
Area	>=5000	905394	Pass
Height		395208	
Amount		905394 Counts	

Match Types: N-F : Normal-Forward

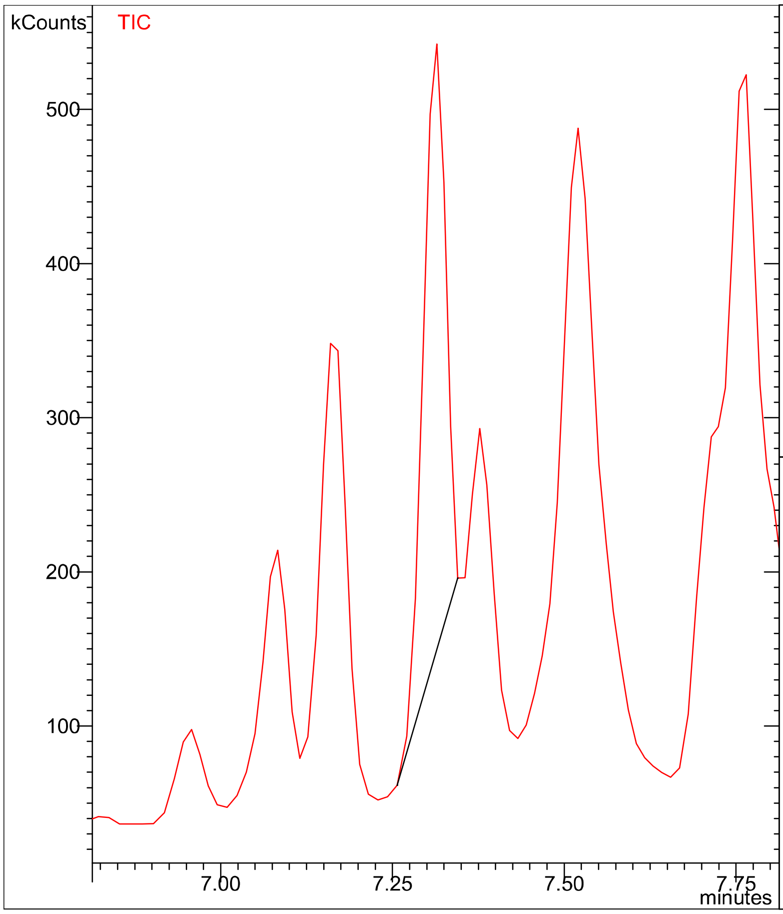

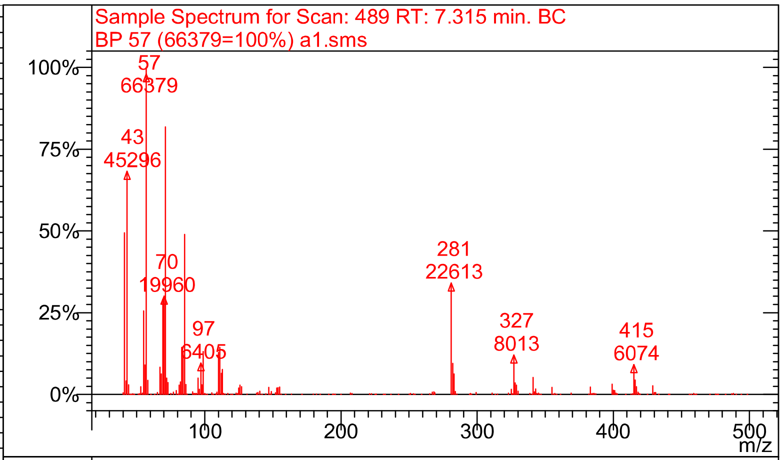

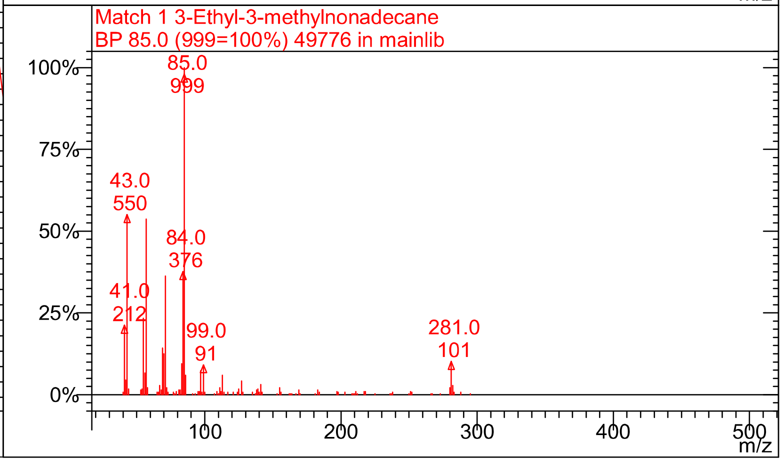

Seg 2, <No Description>, Time: 4.00-30.00

Sample ID:	A1	Operator:	
Instrument ID:	MS Instrument #1	Last Calibration:	None
Measurement Type:	Area	Calibration Type:	External Standard
Acquisition Date:	10/20/2020 1:52 PM	Data File:	d:\201020_ils\a1.sms
Calculation Date:	10/20/2020 5:01 PM	Method:	d:\ms\method\fa.mth
Sample Type:	Analysis		
Inj. Sample Notes:	None		

Compound Information

Peak Name:	1s,4R,7R,11R-1,3,4,7-Tetramethyltricyclo			
Result Index:	7	Peak Number: 7	CAS Number: 137235-42-8	TIC

Identification

Parameter	Specification	Actual	Status
Search Type	Library Search		
Retention Time		7.965 min.	
1st Match Library		mainlib	
1st Match Entry No.		133723	
2nd Match Library		mainlib	
2nd Match Entry No.		144785	
3rd Match Library		mainlib	
3rd Match Entry No.		171503	
Forward Match	N-F >= 500	731	Pass
Reverse Match		748	

Integration and Quantitation

Parameter	Specification	Actual	Status
Quan Ions	RIC		
RF Used	1.000		
Area	>=5000	6.890e+6	Pass
Height		1.354e+6	
Amount		6889571 Counts	

Match Types: N-F : Normal-Forward

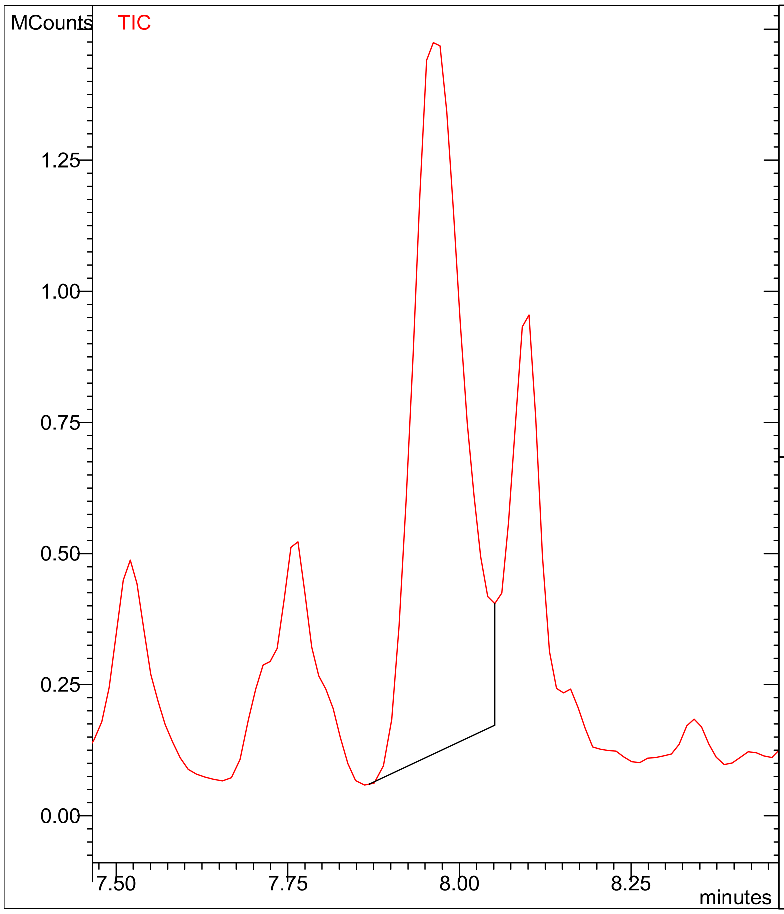

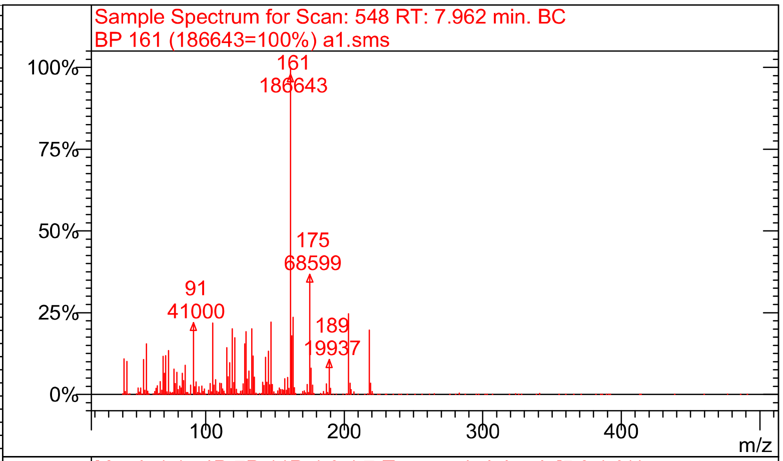

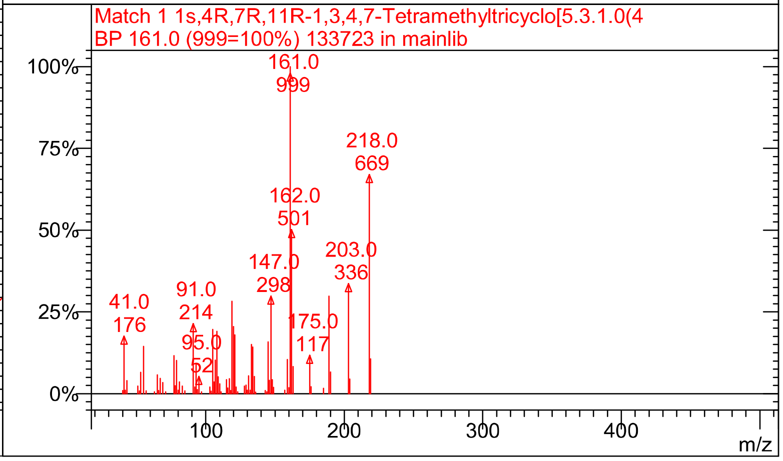

Seg 2, <No Description>, Time: 4.00-30.00

Sample ID:	A1	Operator:	
Instrument ID:	MS Instrument #1	Last Calibration:	None
Measurement Type:	Area	Calibration Type:	External Standard
Acquisition Date:	10/20/2020 1:52 PM	Data File:	d:\201020_ils\a1.sms
Calculation Date:	10/20/2020 5:01 PM	Method:	d:\ms\method\fa.mth
Sample Type:	Analysis		
Inj. Sample Notes:	None		

Compound Information

Peak Name:	Nonadecane		
Result Index:	8	Peak Number: 8	CAS Number: 629-92-5	TIC

Identification

Parameter	Specification	Actual	Status
Search Type	Library Search		
Retention Time		8.097 min.	
1st Match Library		replib	
1st Match Entry No.		5836	
2nd Match Library		mainlib	
2nd Match Entry No.		23579	
3rd Match Library		mainlib	
3rd Match Entry No.		22711	
Forward Match	N-F >= 500	800	Pass
Reverse Match		850	

Integration and Quantitation

Parameter	Specification	Actual	Status
Quan Ions	RIC		
RF Used	1.000		
Area	>=5000	2.215e+6	Pass
Height		753782	
Amount		2214696 Counts	

Match Types: N-F : Normal-Forward

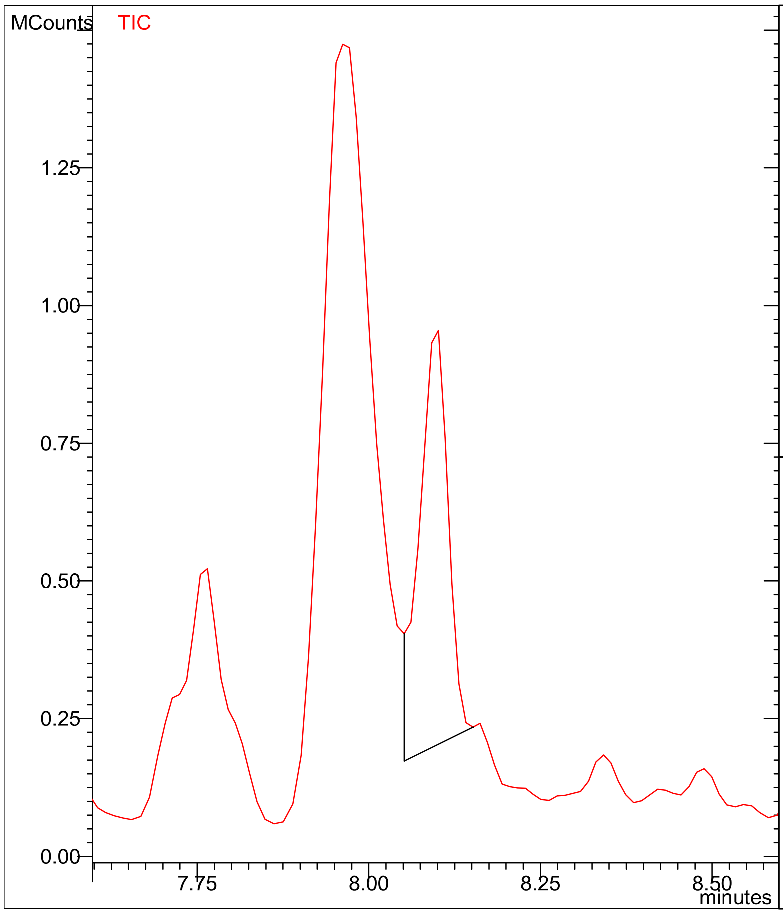

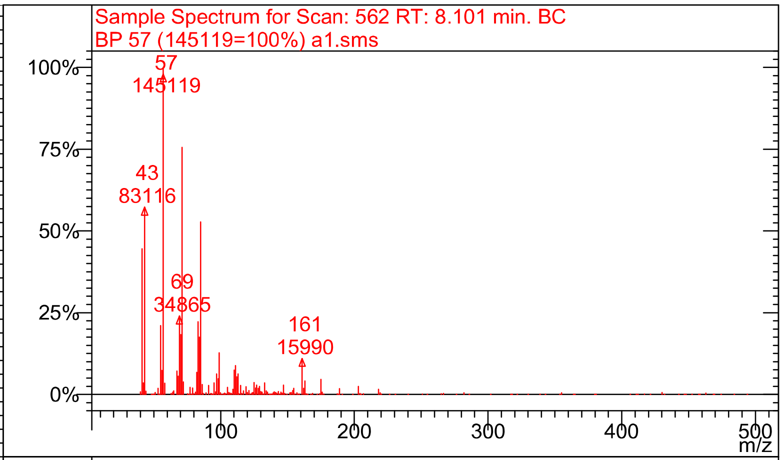

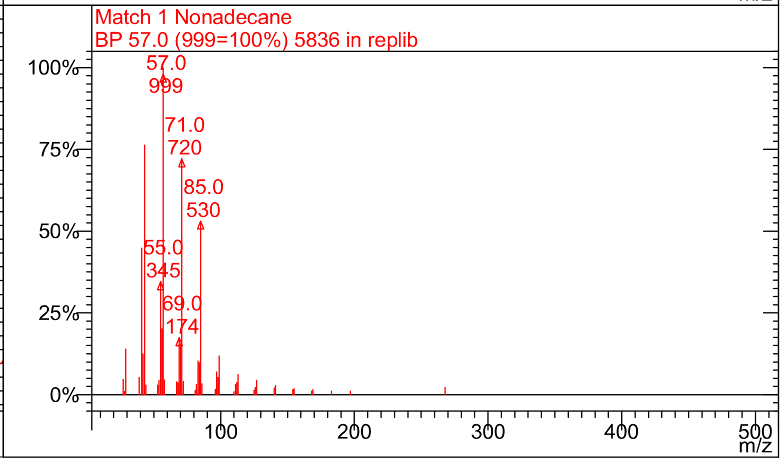

Seg 2, <No Description>, Time: 4.00-30.00

Sample ID:	A1	Operator:	
Instrument ID:	MS Instrument #1	Last Calibration:	None
Measurement Type:	Area	Calibration Type:	External Standard
Acquisition Date:	10/20/2020 1:52 PM	Data File:	d:\201020_ils\a1.sms
Calculation Date:	10/20/2020 5:01 PM	Method:	d:\ms\method\fa.mth
Sample Type:	Analysis		
Inj. Sample Notes:	None		

Compound Information

Peak Name:	Phenol, 2,4-bis(1,1-dimethylethyl)-			
Result Index:	9	Peak Number: 9	CAS Number: 96-76-4	TIC

Identification

Parameter	Specification	Actual	Status
Search Type	Library Search		
Retention Time		8.674 min.	
1st Match Library		mainlib	
1st Match Entry No.		156338	
2nd Match Library		replib	
2nd Match Entry No.		25771	
3rd Match Library		replib	
3rd Match Entry No.		25767	
Forward Match	N-F >= 500	908	Pass
Reverse Match		908	

Integration and Quantitation

Parameter	Specification	Actual	Status
Quan Ions	RIC		
RF Used	1.000		
Area	>=5000	2.368e+7	Pass
Height		7.631e+6	
Amount		23679834 Counts	

Match Types: N-F : Normal-Forward

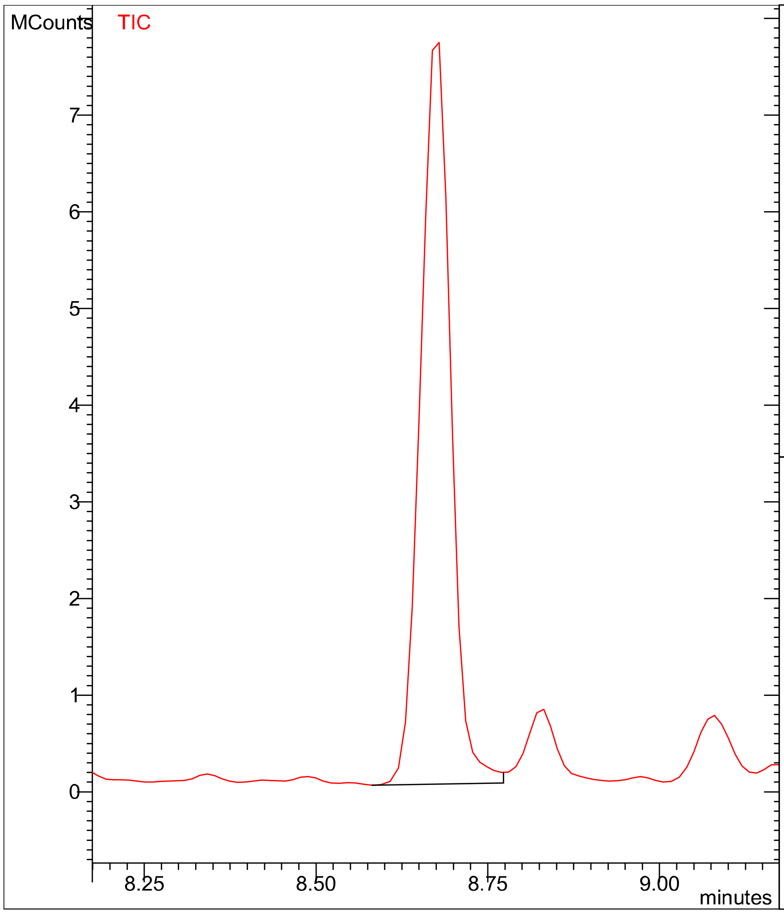

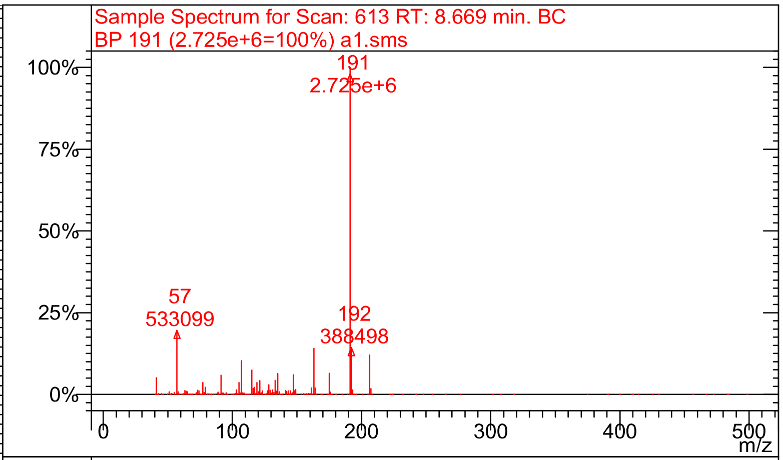

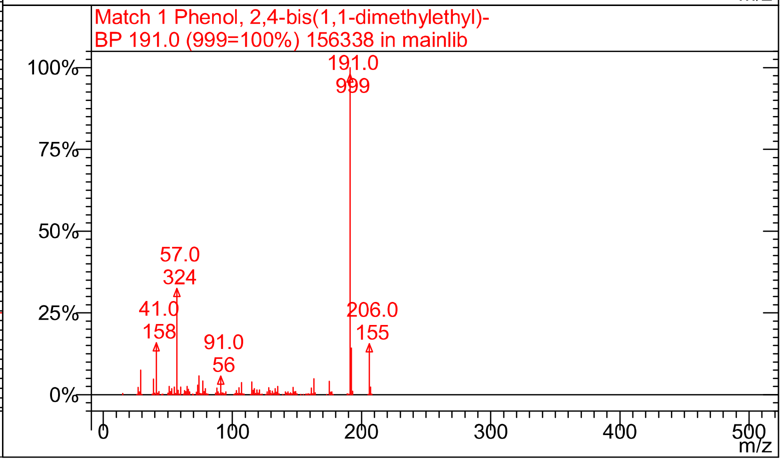

Seg 2, <No Description>, Time: 4.00-30.00

Sample ID:	A1	Operator:	
Instrument ID:	MS Instrument #1	Last Calibration:	None
Measurement Type:	Area	Calibration Type:	External Standard
Acquisition Date:	10/20/2020 1:52 PM	Data File:	d:\201020_ils\a1.sms
Calculation Date:	10/20/2020 5:01 PM	Method:	d:\ms\method\fa.mth
Sample Type:	Analysis		
Inj. Sample Notes:	None		

Compound Information

Peak Name:	11-Methyldodecanol			
Result Index:	10	Peak Number: 10	CAS Number: 85763-57-1	TIC

Identification

Parameter	Specification	Actual	Status
Search Type	Library Search		
Retention Time		8.828 min.	
1st Match Library		mainlib	
1st Match Entry No.		31330	
2nd Match Library		mainlib	
2nd Match Entry No.		18916	
3rd Match Library		replib	
3rd Match Entry No.		963	
Forward Match	N-F >= 500	808	Pass
Reverse Match		834	

Integration and Quantitation

Parameter	Specification	Actual	Status
Quan Ions	RIC		
RF Used	1.000		
Area	>=5000	2.349e+6	Pass
Height		754532	
Amount		2348927 Counts	

Match Types: N-F : Normal-Forward

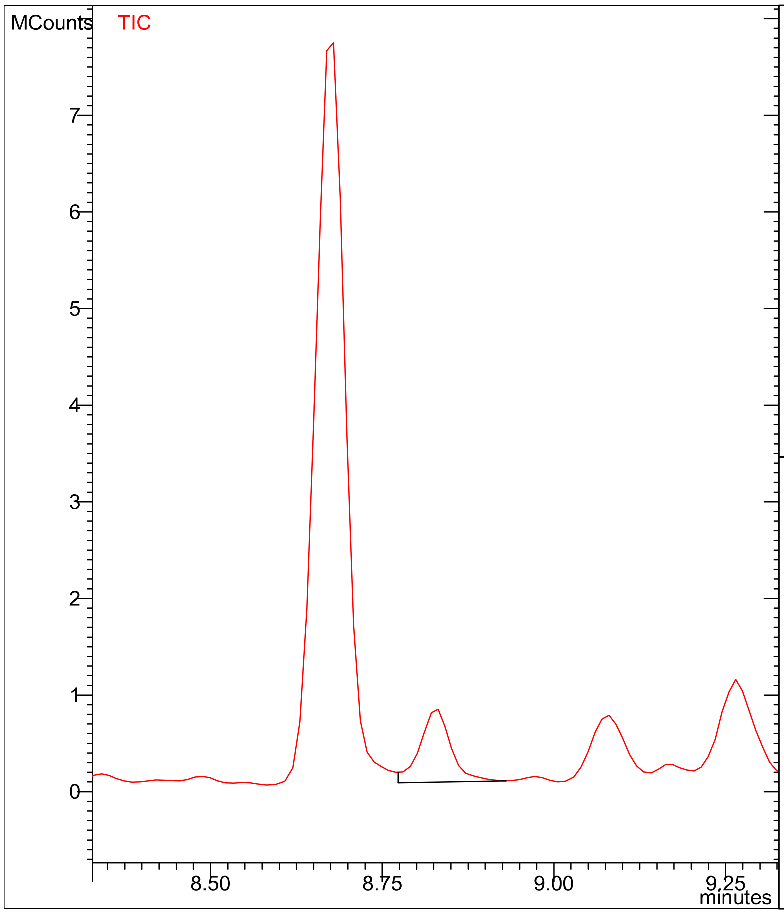

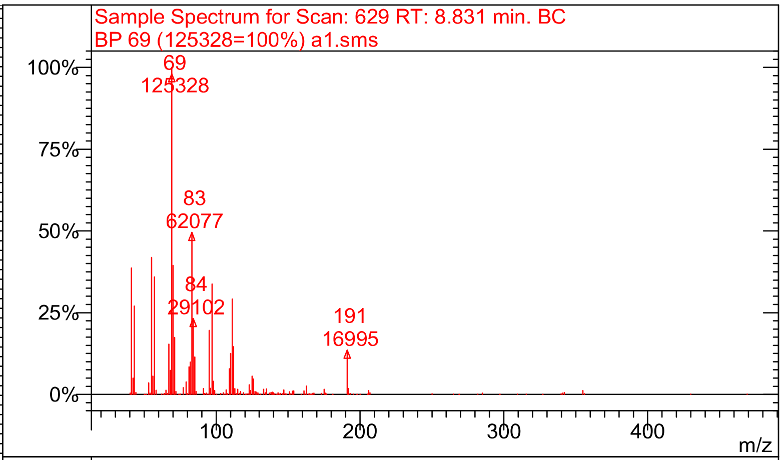

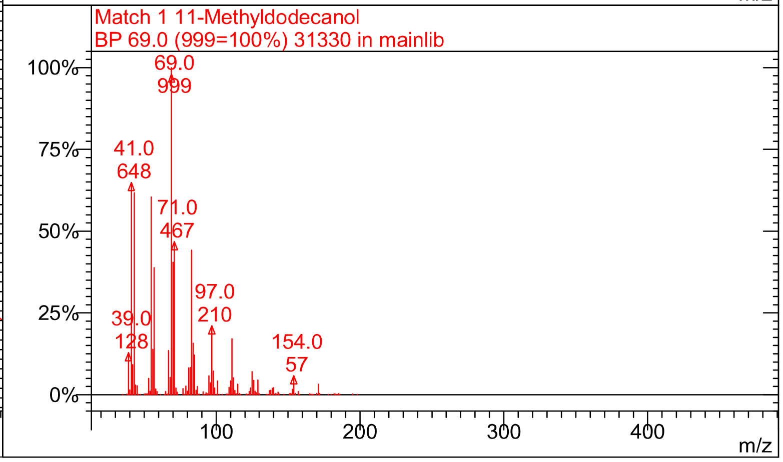

Seg 2, <No Description>, Time: 4.00-30.00

Sample ID:	A1	Operator:	
Instrument ID:	MS Instrument #1	Last Calibration:	None
Measurement Type:	Area	Calibration Type:	External Standard
Acquisition Date:	10/20/2020 1:52 PM	Data File:	d:\201020_ils\a1.sms
Calculation Date:	10/20/2020 5:01 PM	Method:	d:\ms\method\fa.mth
Sample Type:	Analysis		
Inj. Sample Notes:	None		

Compound Information

Peak Name: 11-Methyldodecanol
Result Index: 11 Peak Number: 11 CAS Number: 85763-57-1 TIC

Identification

Parameter	Specification	Actual	Status
Search Type	Library Search		
Retention Time		9.078 min.	
1st Match Library		mainlib	
1st Match Entry No.		31330	
2nd Match Library		replib	
2nd Match Entry No.		5738	
3rd Match Library		mainlib	
3rd Match Entry No.		18839	
Forward Match	N-F >= 500	815	Pass
Reverse Match		822	

Integration and Quantitation

Parameter	Specification	Actual	Status
Quan Ions	RIC		
RF Used	1.000		
Area	>=5000	2.468e+6	Pass
Height		693366	
Amount		2467639 Counts	

Match Types: N-F : Normal-Forward

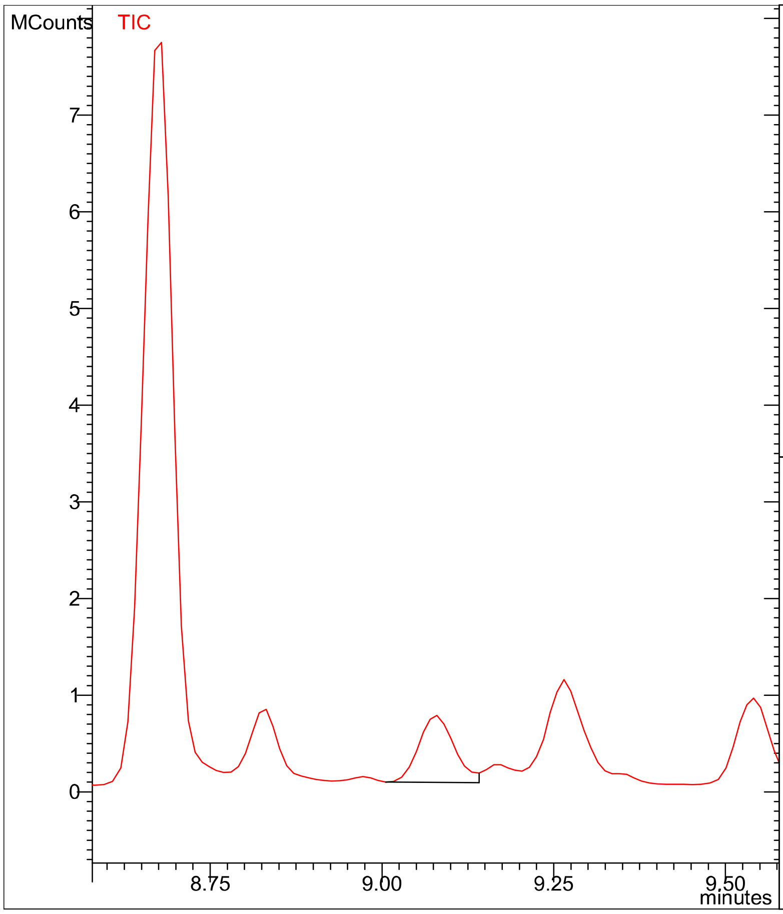

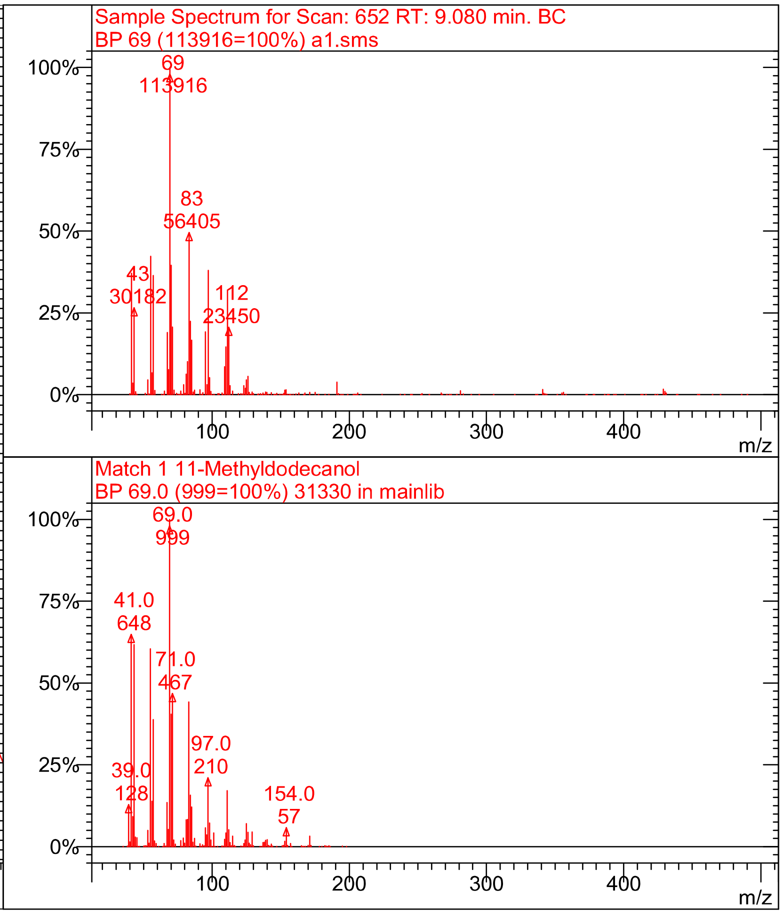

Seg 2, <No Description>, Time: 4.00-30.00

Sample ID:	A1	Operator:	
Instrument ID:	MS Instrument #1	Last Calibration:	None
Measurement Type:	Area	Calibration Type:	External Standard
Acquisition Date:	10/20/2020 1:52 PM	Data File:	d:\201020_ils\a1.sms
Calculation Date:	10/20/2020 5:01 PM	Method:	d:\ms\method\fa.mth
Sample Type:	Analysis		
Inj. Sample Notes:	None		

Compound Information

Peak Name:	1-Dodecanol, 2-hexyl-			
Result Index:	12	Peak Number: 12	CAS Number: 110225-00-8	TIC

Identification

Parameter	Specification	Actual	Status
Search Type	Library Search		
Retention Time		9.265 min.	
1st Match Library		mainlib	
1st Match Entry No.		22630	
2nd Match Library		replib	
2nd Match Entry No.		6096	
3rd Match Library		replib	
3rd Match Entry No.		5843	
Forward Match	N-F >= 500	855	Pass
Reverse Match		855	

Integration and Quantitation

Parameter	Specification	Actual	Status
Quan Ions	RIC		
RF Used	1.000		
Area	>=5000	4.810e+6	Pass
Height		1.072e+6	
Amount		4809809 Counts	

Match Types: N-F : Normal-Forward

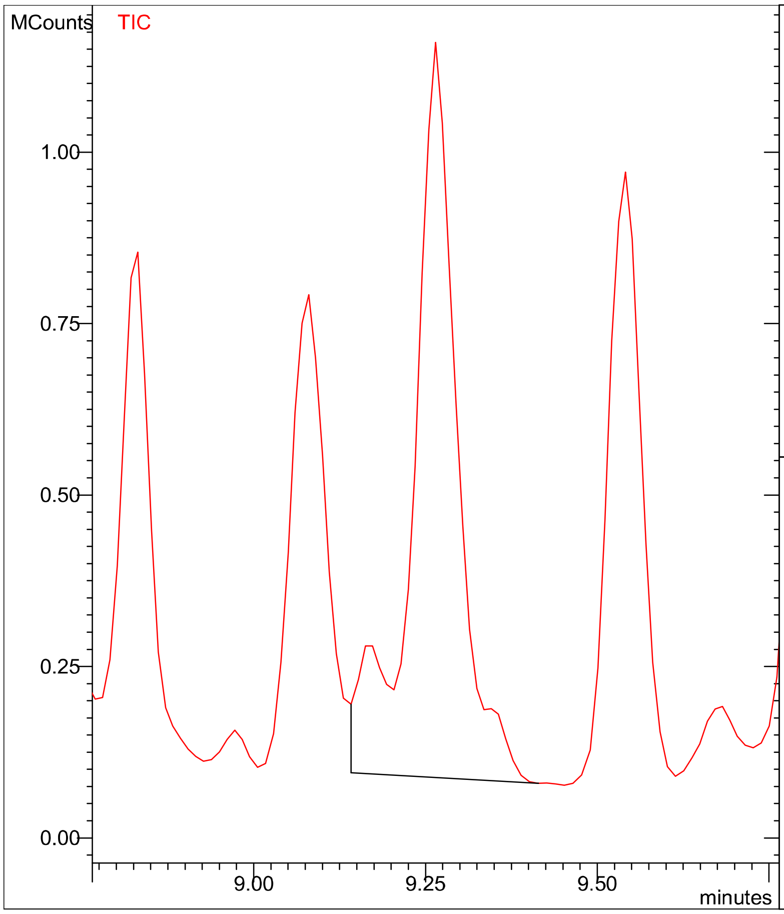

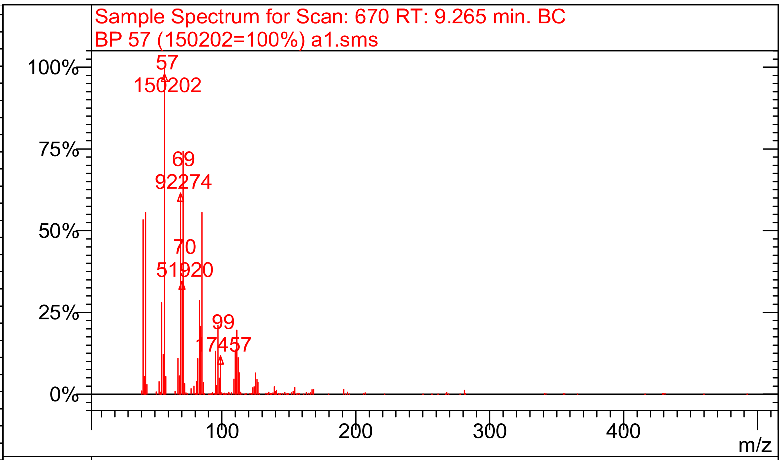

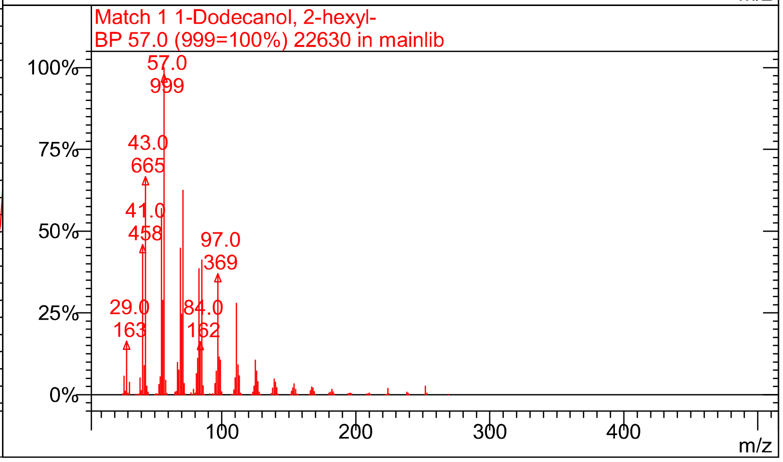

Seg 2, <No Description>, Time: 4.00-30.00

Sample ID:	A1	Operator:	
Instrument ID:	MS Instrument #1	Last Calibration:	None
Measurement Type:	Area	Calibration Type:	External Standard
Acquisition Date:	10/20/2020 1:52 PM	Data File:	d:\201020_ils\a1.sms
Calculation Date:	10/20/2020 5:01 PM	Method:	d:\ms\method\fa.mth
Sample Type:	Analysis		
Inj. Sample Notes:	None		

Compound Information

Peak Name: 11-Methyldodecanol
Result Index: 13 Peak Number: 13 CAS Number: 85763-57-1 TIC

Identification

Parameter	Specification	Actual	Status
Search Type	Library Search		
Retention Time		9.540 min.	
1st Match Library		mainlib	
1st Match Entry No.		31330	
2nd Match Library		replib	
2nd Match Entry No.		5738	
3rd Match Library		mainlib	
3rd Match Entry No.		18829	
Forward Match	N-F >= 500	838	Pass
Reverse Match		838	

Integration and Quantitation

Parameter	Specification	Actual	Status
Quan Ions	RIC		
RF Used	1.000		
Area	>=5000	2.943e+6	Pass
Height		886506	
Amount		2943392 Counts	

Match Types: N-F : Normal-Forward

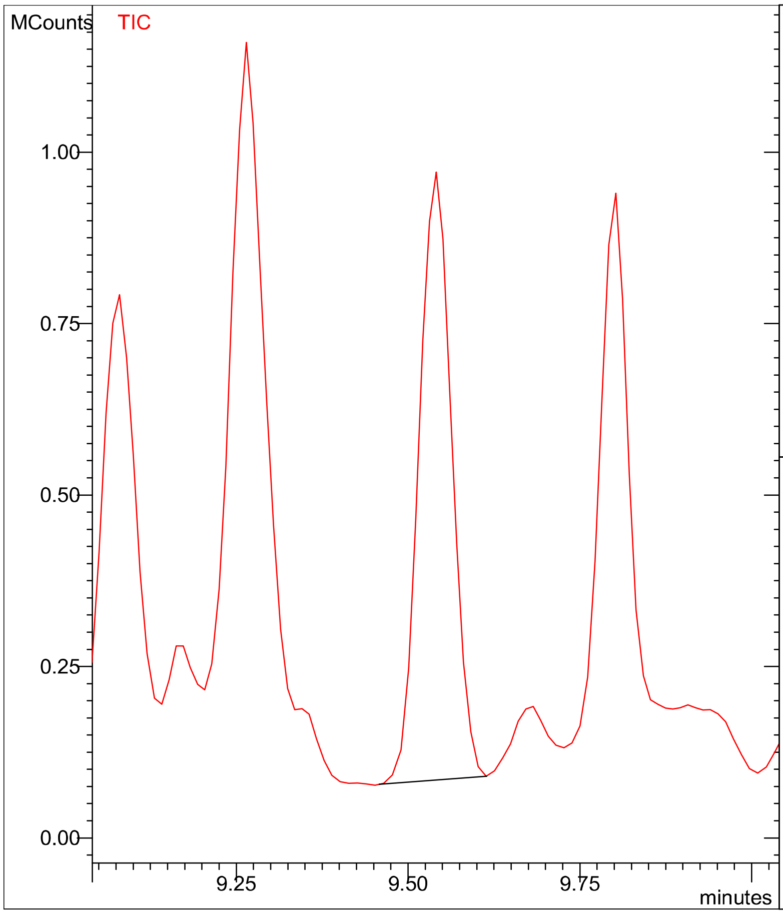

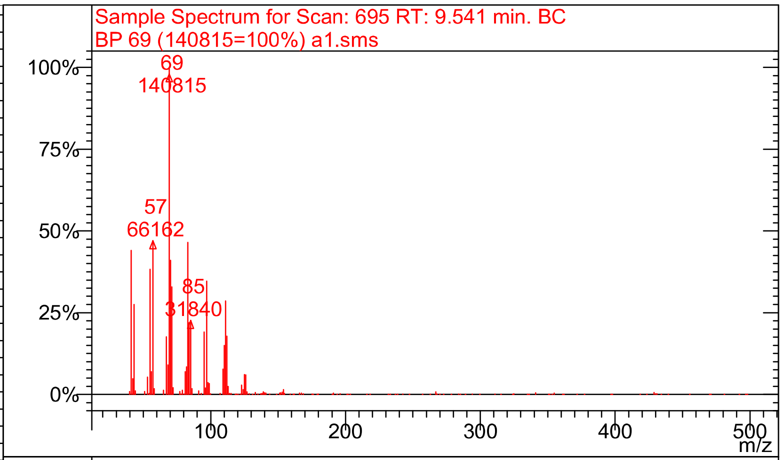

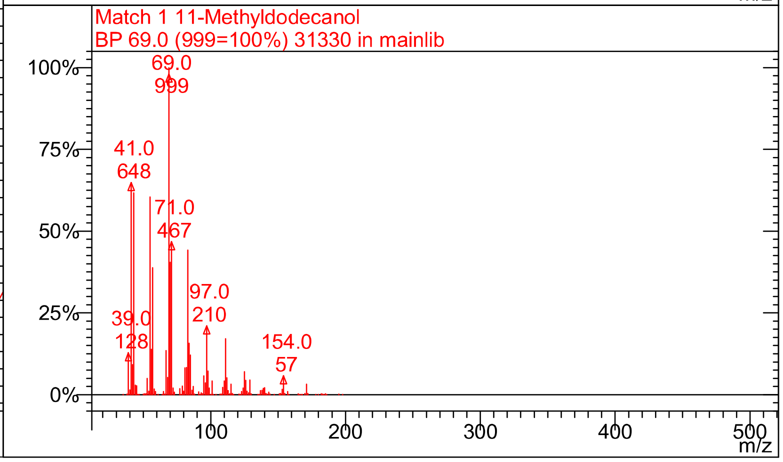

Seg 2, <No Description>, Time: 4.00-30.00

Sample ID:	A1	Operator:	
Instrument ID:	MS Instrument #1	Last Calibration:	None
Measurement Type:	Area	Calibration Type:	External Standard
Acquisition Date:	10/20/2020 1:52 PM	Data File:	d:\201020_ils\a1.sms
Calculation Date:	10/20/2020 5:01 PM	Method:	d:\ms\method\fa.mth
Sample Type:	Analysis		
Inj. Sample Notes:	None		

Compound Information

Peak Name:	11-Methyldodecanol			
Result Index:	14	Peak Number: 14	CAS Number: 85763-57-1	TIC

Identification

Parameter	Specification	Actual	Status
Search Type	Library Search		
Retention Time		9.801 min.	
1st Match Library		mainlib	
1st Match Entry No.		31330	
2nd Match Library		replib	
2nd Match Entry No.		6096	
3rd Match Library		mainlib	
3rd Match Entry No.		23004	
Forward Match	N-F >= 500	769	Pass
Reverse Match		835	

Integration and Quantitation

Parameter	Specification	Actual	Status
Quan Ions	RIC		
RF Used	1.000		
Area	>=5000	2.176e+6	Pass
Height		782330	
Amount		2176025 Counts	

Match Types: N-F : Normal-Forward

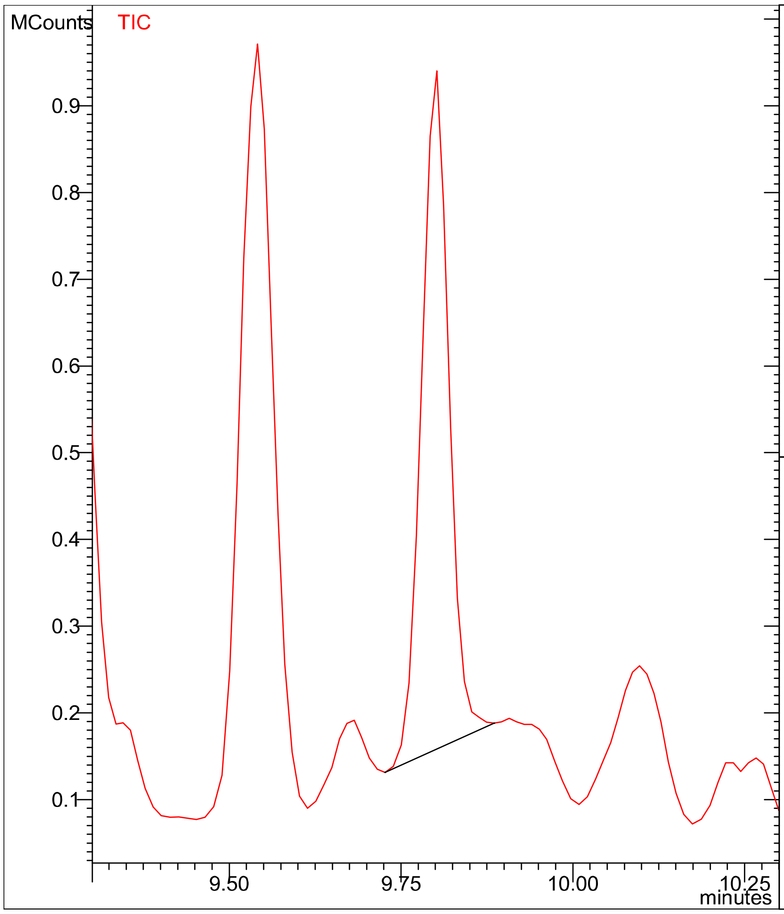

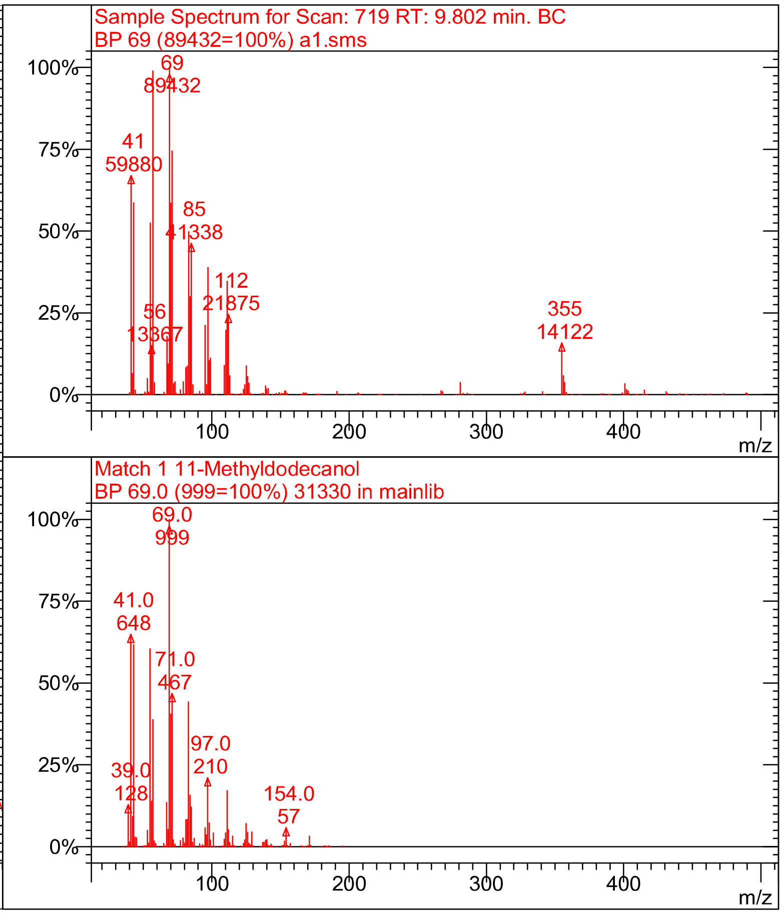

Seg 2, <No Description>, Time: 4.00-30.00

Sample ID:	A1	Operator:	
Instrument ID:	MS Instrument #1	Last Calibration:	None
Measurement Type:	Area	Calibration Type:	External Standard
Acquisition Date:	10/20/2020 1:52 PM	Data File:	d:\201020_ils\a1.sms
Calculation Date:	10/20/2020 5:01 PM	Method:	d:\ms\method\fa.mth
Sample Type:	Analysis		
Inj. Sample Notes:	None		

Compound Information

Peak Name:	Tritetracontane			
Result Index:	15	Peak Number: 15	CAS Number: 7098-21-7	TIC

Identification

Parameter	Specification	Actual	Status
Search Type	Library Search		
Retention Time		13.669 min.	
1st Match Library		mainlib	
1st Match Entry No.		23579	
2nd Match Library		replib	
2nd Match Entry No.		6096	
3rd Match Library		replib	
3rd Match Entry No.		5843	
Forward Match	N-F >= 500	838	Pass
Reverse Match		838	

Integration and Quantitation

Parameter	Specification	Actual	Status
Quan Ions	RIC		
RF Used	1.000		
Area	>=5000	1.399e+6	Pass
Height		525284	
Amount		1398664 Counts	

Match Types: N-F : Normal-Forward

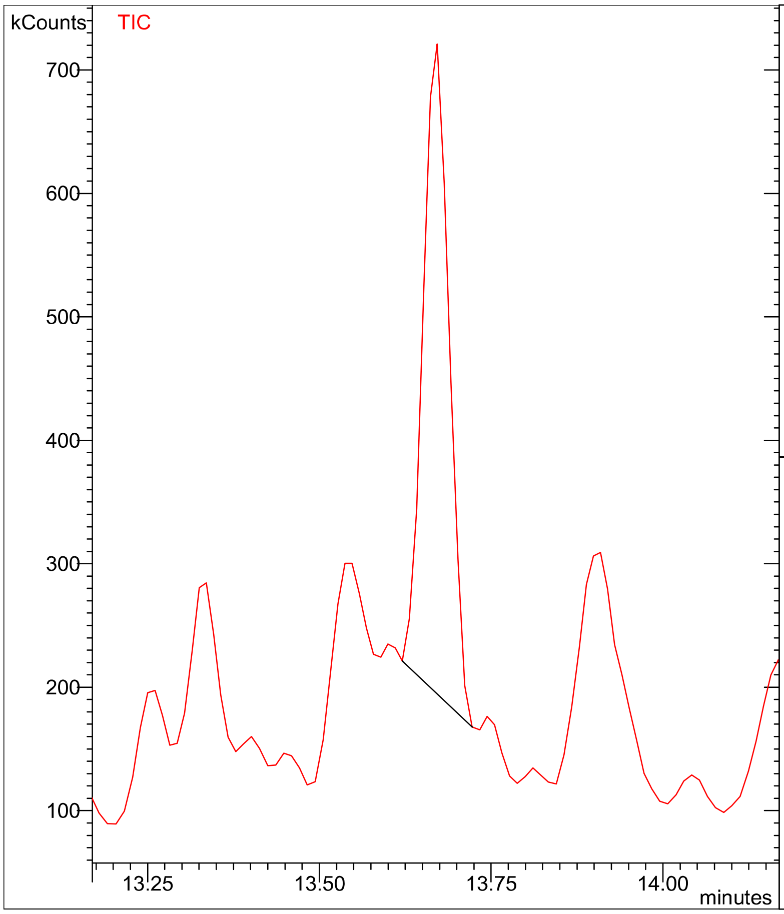

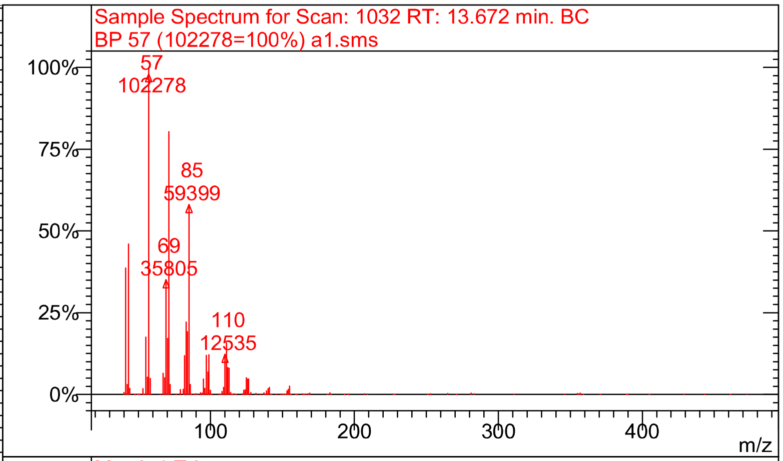

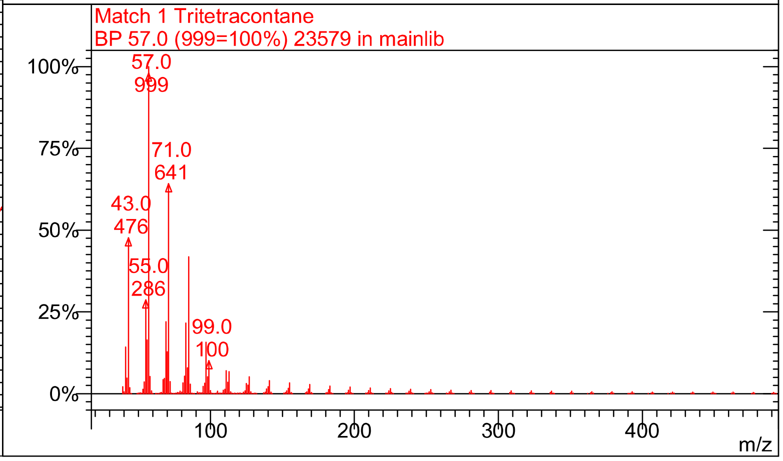

Seg 2, <No Description>, Time: 4.00-30.00

Sample ID:	A1	Operator:	
Instrument ID:	MS Instrument #1	Last Calibration:	None
Measurement Type:	Area	Calibration Type:	External Standard
Acquisition Date:	10/20/2020 1:52 PM	Data File:	d:\201020_ils\a1.sms
Calculation Date:	10/20/2020 5:01 PM	Method:	d:\ms\method\fa.mth
Sample Type:	Analysis		
Inj. Sample Notes:	None		

Compound Information

Peak Name: 11-Methyldodecanol
Result Index: 16 Peak Number: 16 CAS Number: 85763-57-1 TIC

Identification

Parameter	Specification	Actual	Status
Search Type	Library Search		
Retention Time		14.450 min.	
1st Match Library		mainlib	
1st Match Entry No.		31330	
2nd Match Library		replib	
2nd Match Entry No.		5738	
3rd Match Library		replib	
3rd Match Entry No.		2169	
Forward Match	N-F >= 500	802	Pass
Reverse Match		802	

Integration and Quantitation

Parameter	Specification	Actual	Status
Quan Ions	RIC		
RF Used	1.000		
Area	>=5000	2.595e+6	Pass
Height		728499	
Amount		2594930 Counts	

Match Types: N-F : Normal-Forward

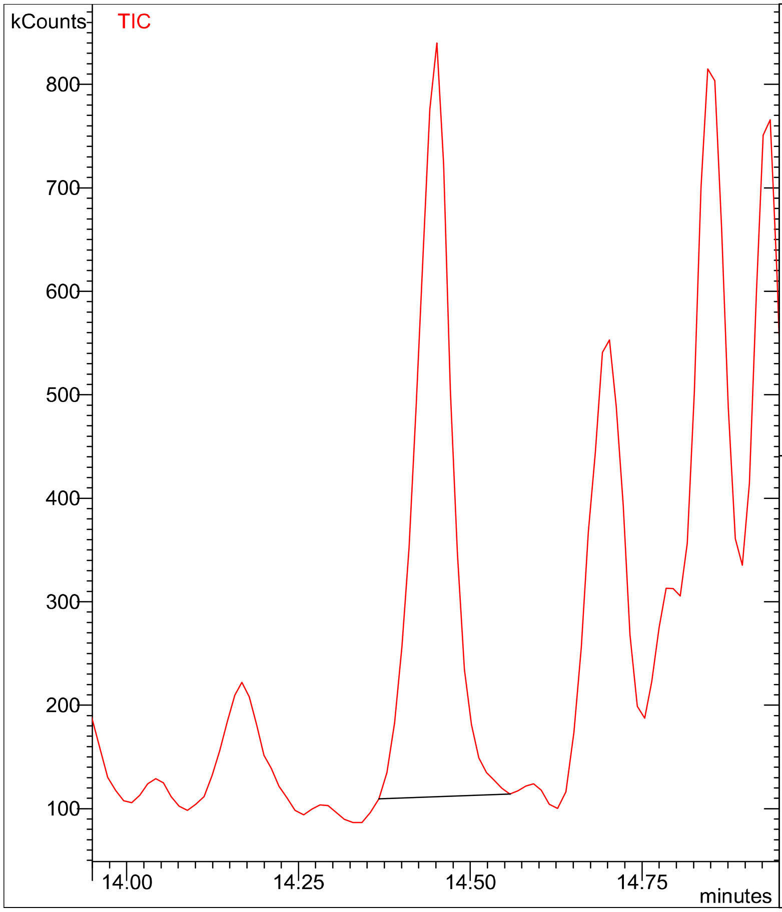

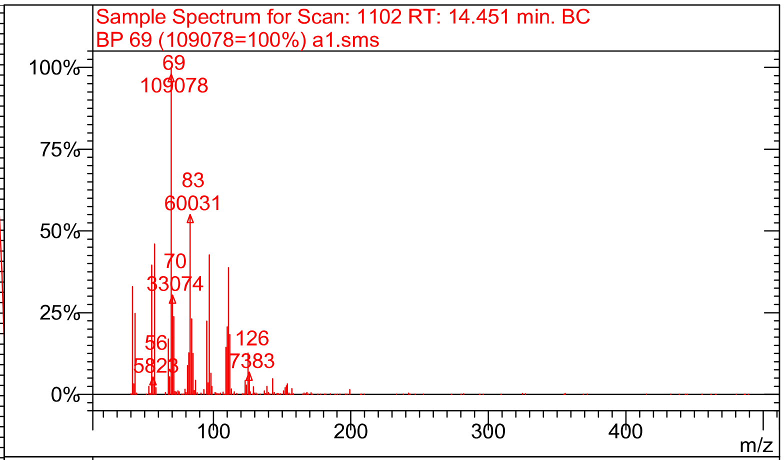

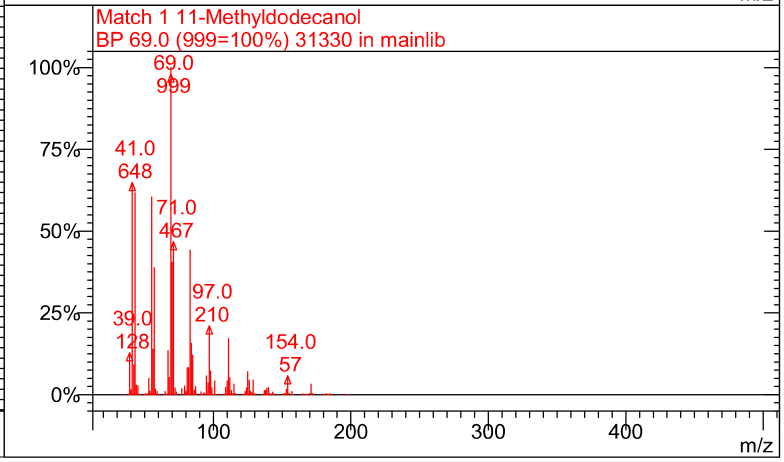

Seg 2, <No Description>, Time: 4.00-30.00

Sample ID:	A1	Operator:	
Instrument ID:	MS Instrument #1	Last Calibration:	None
Measurement Type:	Area	Calibration Type:	External Standard
Acquisition Date:	10/20/2020 1:52 PM	Data File:	d:\201020_ils\a1.sms
Calculation Date:	10/20/2020 5:01 PM	Method:	d:\ms\method\fa.mth
Sample Type:	Analysis		
Inj. Sample Notes:	None		

Compound Information

Peak Name:	1-Decanol, 2-hexyl-			
Result Index:	17	Peak Number: 17	CAS Number: 2425-77-6	TIC

Identification

Parameter	Specification	Actual	Status
Search Type	Library Search		
Retention Time		14.850 min.	
1st Match Library		replib	
1st Match Entry No.		6096	
2nd Match Library		mainlib	
2nd Match Entry No.		23579	
3rd Match Library		replib	
3rd Match Entry No.		5843	
Forward Match	N-F >= 500	857	Pass
Reverse Match		857	

Integration and Quantitation

Parameter	Specification	Actual	Status
Quan Ions	RIC		
RF Used	1.000		
Area	>=5000	1.550e+6	Pass
Height		545671	
Amount		1550447 Counts	

Match Types: N-F : Normal-Forward

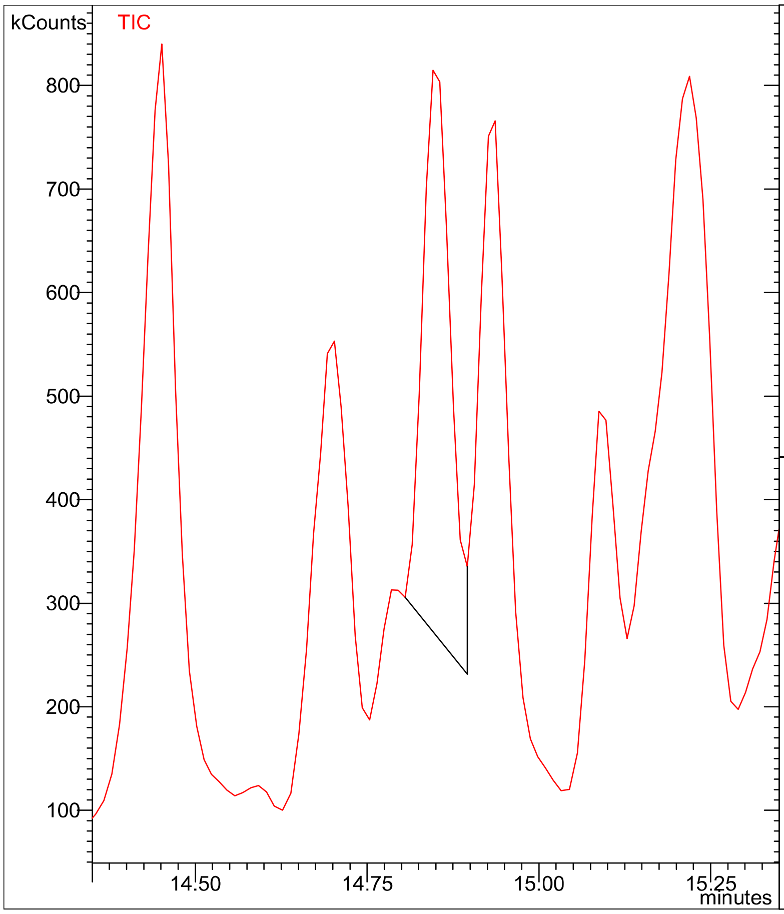

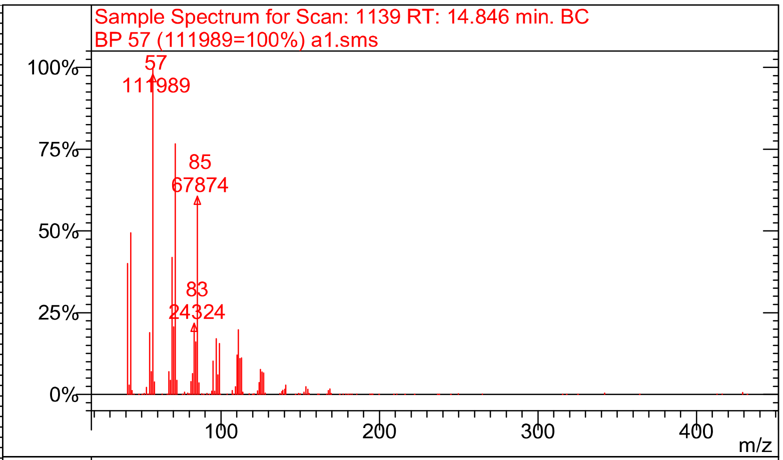

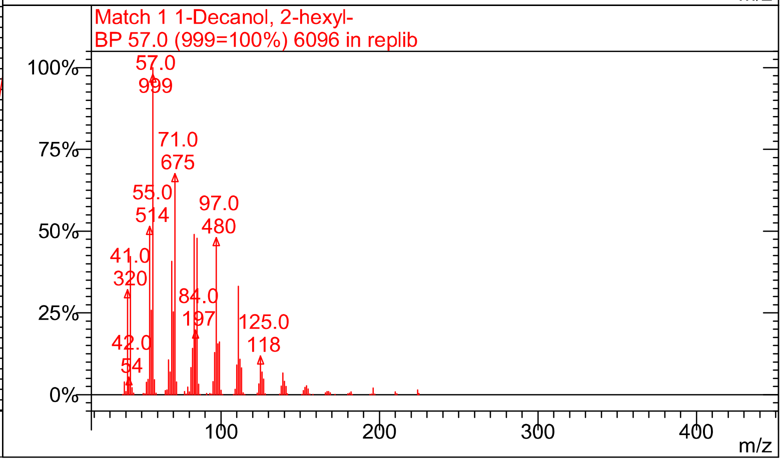

Seg 2, <No Description>, Time: 4.00-30.00

Sample ID:	A1	Operator:	
Instrument ID:	MS Instrument #1	Last Calibration:	None
Measurement Type:	Area	Calibration Type:	External Standard
Acquisition Date:	10/20/2020 1:52 PM	Data File:	d:\201020_ils\a1.sms
Calculation Date:	10/20/2020 5:01 PM	Method:	d:\ms\method\fa.mth
Sample Type:	Analysis		
Inj. Sample Notes:	None		

Compound Information

Peak Name:	11-Methyldodecanol			
Result Index:	18	Peak Number: 18	CAS Number: 85763-57-1	TIC

Identification

Parameter	Specification	Actual	Status
Search Type	Library Search		
Retention Time		14.932 min.	
1st Match Library		mainlib	
1st Match Entry No.		31330	
2nd Match Library		mainlib	
2nd Match Entry No.		7364	
3rd Match Library		replib	
3rd Match Entry No.		2169	
Forward Match	N-F >= 500	678	Pass
Reverse Match		812	

Integration and Quantitation

Parameter	Specification	Actual	Status
Quan Ions	RIC		
RF Used	1.000		
Area	>=5000	1.575e+6	Pass
Height		564405	
Amount		1574784 Counts	

Match Types: N-F : Normal-Forward

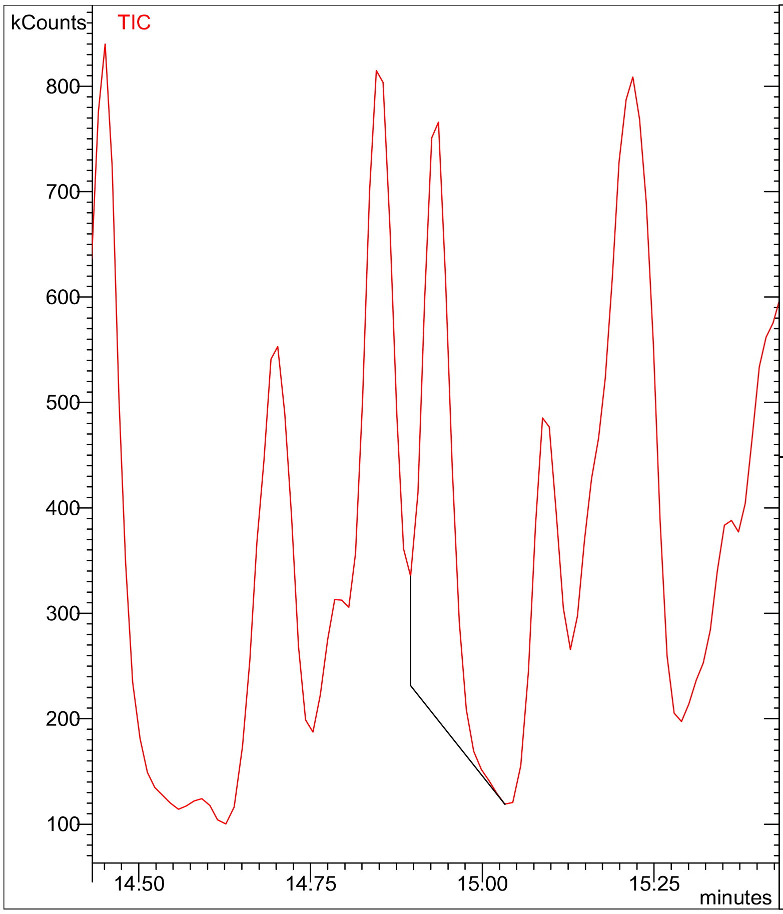

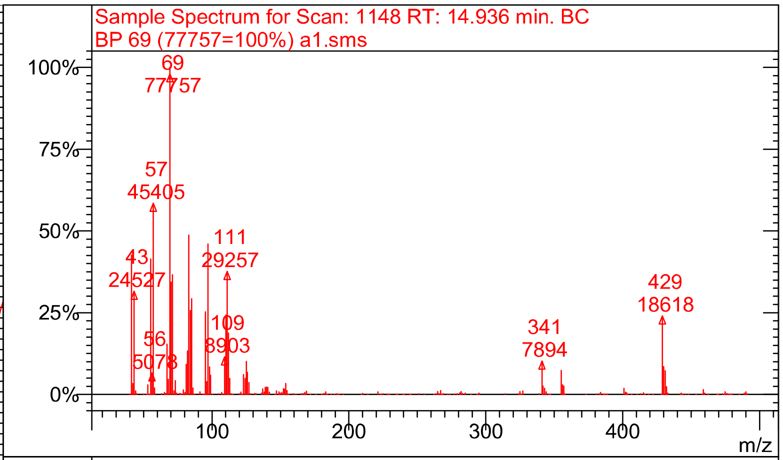

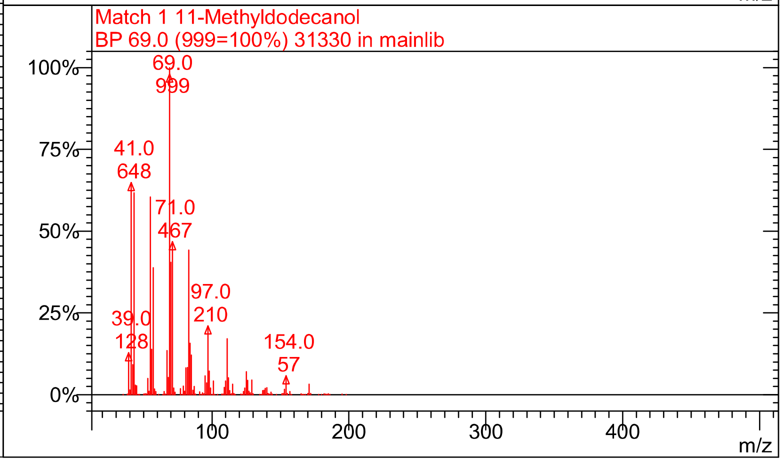

Seg 2, <No Description>, Time: 4.00-30.00

Sample ID:	A1	Operator:	
Instrument ID:	MS Instrument #1	Last Calibration:	None
Measurement Type:	Area	Calibration Type:	External Standard
Acquisition Date:	10/20/2020 1:52 PM	Data File:	d:\201020_ils\a1.sms
Calculation Date:	10/20/2020 5:01 PM	Method:	d:\ms\method\fa.mth
Sample Type:	Analysis		
Inj. Sample Notes:	None		

Compound Information

Peak Name:	1-Dodecanol, 2-hexyl-			
Result Index:	19	Peak Number: 19	CAS Number: 110225-00-8	TIC

Identification

Parameter	Specification	Actual	Status
Search Type	Library Search		
Retention Time		15.092 min.	
1st Match Library		mainlib	
1st Match Entry No.		22630	
2nd Match Library		mainlib	
2nd Match Entry No.		23004	
3rd Match Library		mainlib	
3rd Match Entry No.		22667	
Forward Match	N-F >= 500	829	Pass
Reverse Match		835	

Integration and Quantitation

Parameter	Specification	Actual	Status
Quan Ions	RIC		
RF Used	1.000		
Area	>=5000	614134	Pass
Height		275445	
Amount		614134 Counts	

Match Types: N-F : Normal-Forward

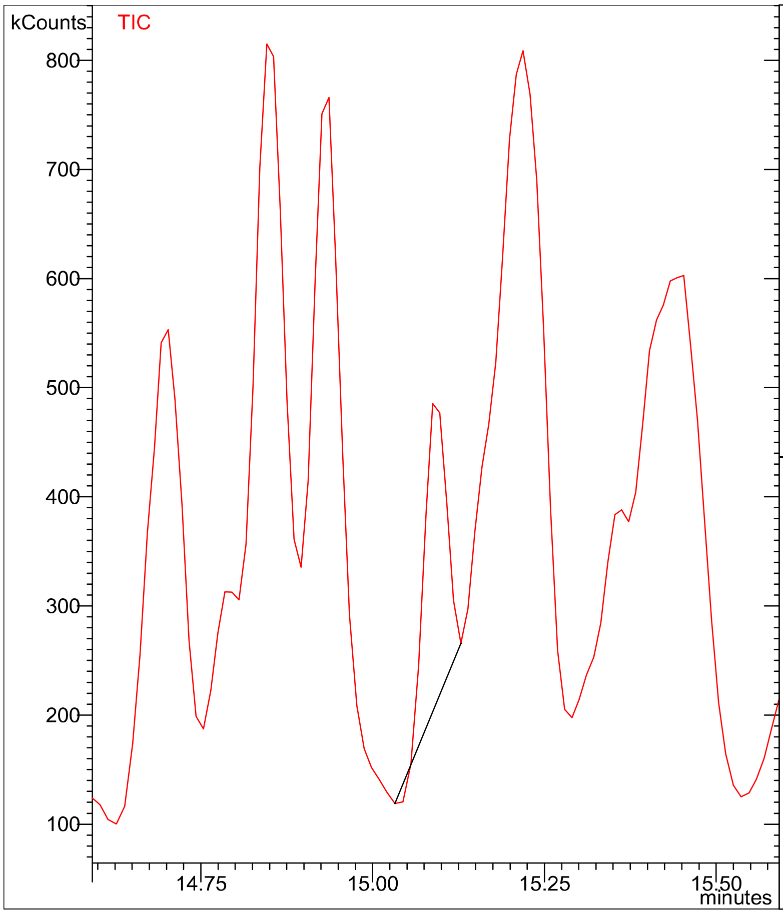

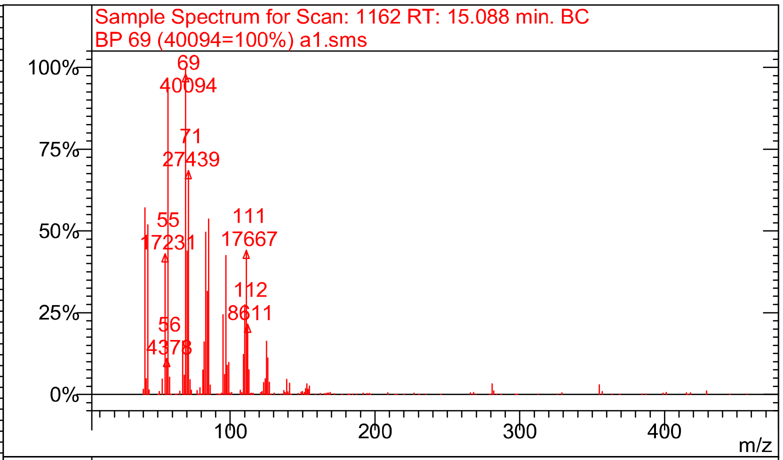

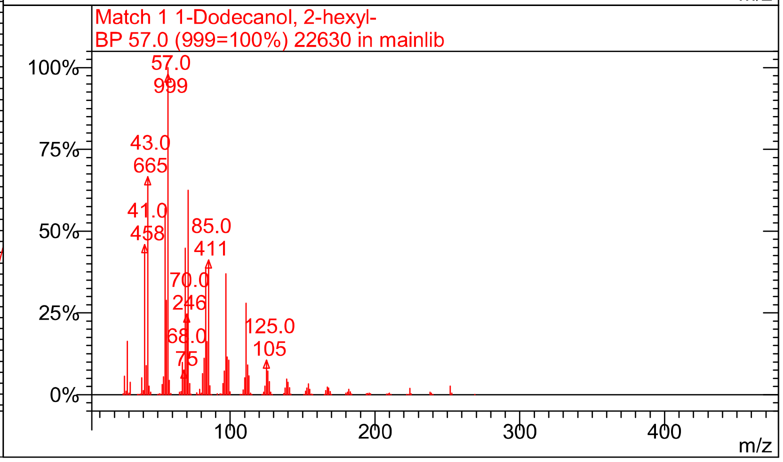

Seg 2, <No Description>, Time: 4.00-30.00

Sample ID:	A1	Operator:	
Instrument ID:	MS Instrument #1	Last Calibration:	None
Measurement Type:	Area	Calibration Type:	External Standard
Acquisition Date:	10/20/2020 1:52 PM	Data File:	d:\201020_ils\a1.sms
Calculation Date:	10/20/2020 5:01 PM	Method:	d:\ms\method\fa.mth
Sample Type:	Analysis		
Inj. Sample Notes:	None		

Compound Information

Peak Name: 11-Methyldodecanol
Result Index: 20 Peak Number: 20 CAS Number: 85763-57-1 TIC

Identification

Parameter	Specification	Actual	Status
Search Type	Library Search		
Retention Time		16.066 min.	
1st Match Library		mainlib	
1st Match Entry No.		31330	
2nd Match Library		mainlib	
2nd Match Entry No.		31860	
3rd Match Library		replib	
3rd Match Entry No.		5738	
Forward Match	N-F >= 500	801	Pass
Reverse Match		801	

Integration and Quantitation

Parameter	Specification	Actual	Status
Quan Ions	RIC		
RF Used	1.000		
Area	>=5000	1.653e+6	Pass
Height		574179	
Amount		1653307 Counts	

Match Types: N-F : Normal-Forward

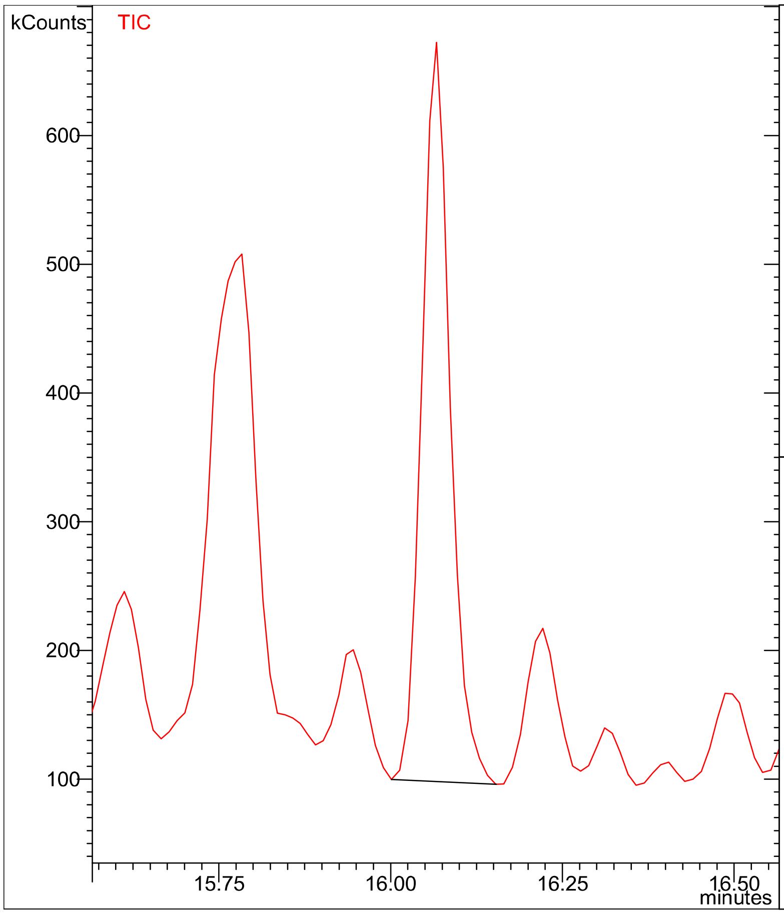

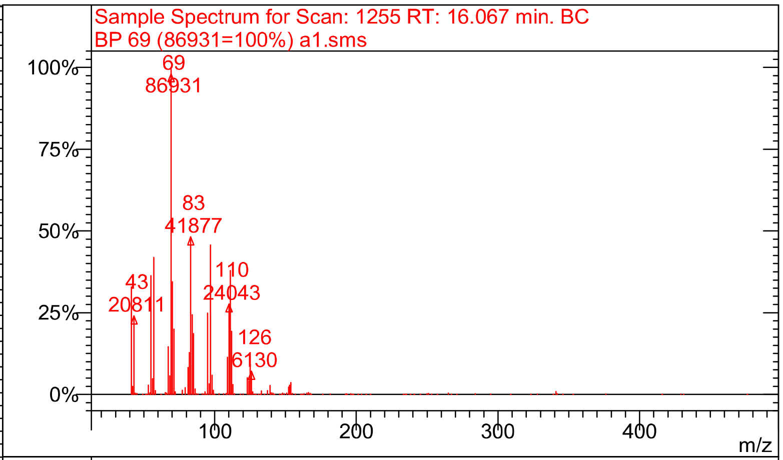

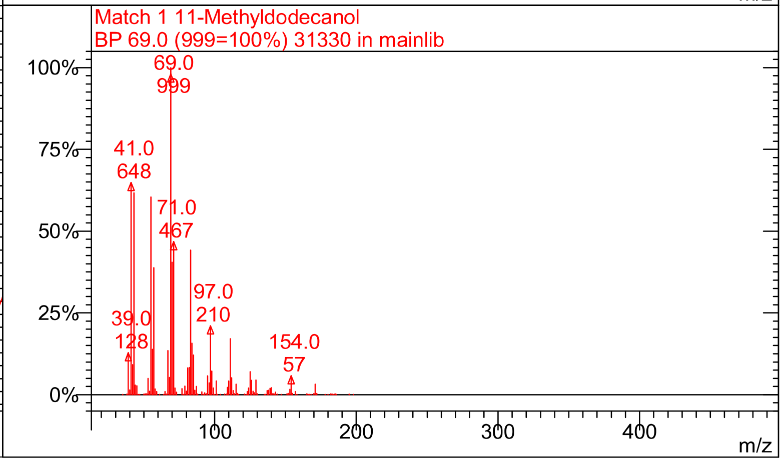

Seg 2, <No Description>, Time: 4.00-30.00

Sample ID:	A1	Operator:	
Instrument ID:	MS Instrument #1	Last Calibration:	None
Measurement Type:	Area	Calibration Type:	External Standard
Acquisition Date:	10/20/2020 1:52 PM	Data File:	d:\201020_ils\a1.sms
Calculation Date:	10/20/2020 5:01 PM	Method:	d:\ms\method\fa.mth
Sample Type:	Analysis		
Inj. Sample Notes:	None		

Compound Information

Peak Name: Pentadecanoic acid, 14-methyl-, methyl e
Result Index: 21   Peak Number: 21   CAS Number: 5129-60-2   TIC

Identification

Parameter	Specification	Actual	Status
Search Type	Library Search		
Retention Time		19.608 min.	
1st Match Library		replib	
1st Match Entry No.		9839	
2nd Match Library		mainlib	
2nd Match Entry No.		40758	
3rd Match Library		replib	
3rd Match Entry No.		9770	
Forward Match	N-F >= 500	795	Pass
Reverse Match		795	

Integration and Quantitation

Parameter	Specification	Actual	Status
Quan Ions	RIC		
RF Used	1.000		
Area	>=5000	3.304e+7	Pass
Height		9.534e+6	
Amount		33044512 Counts	

Match Types: N-F : Normal-Forward

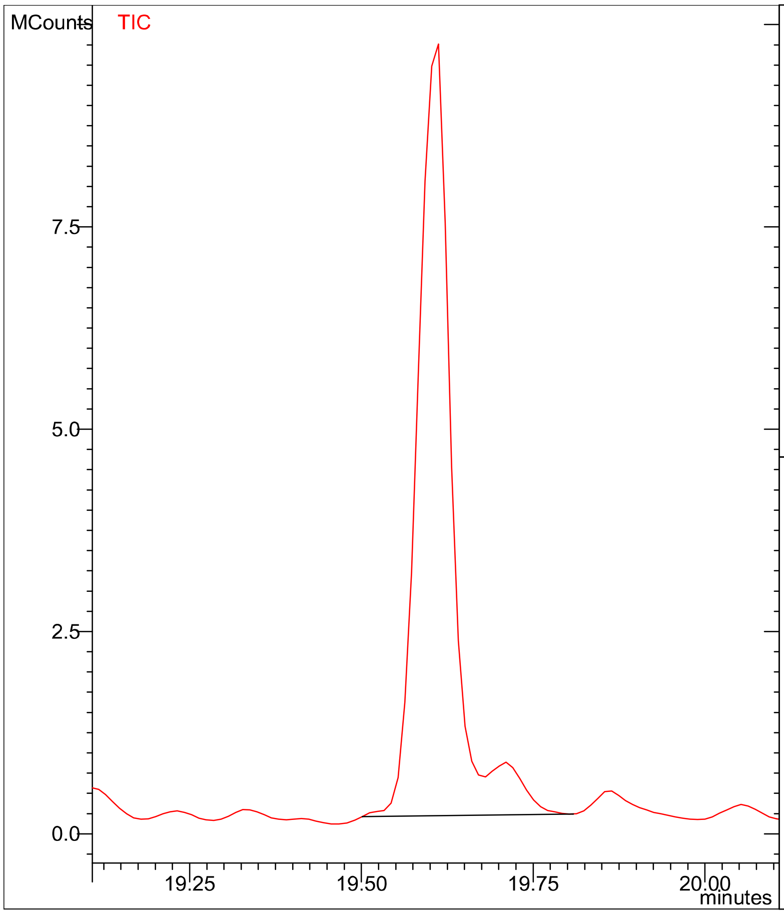

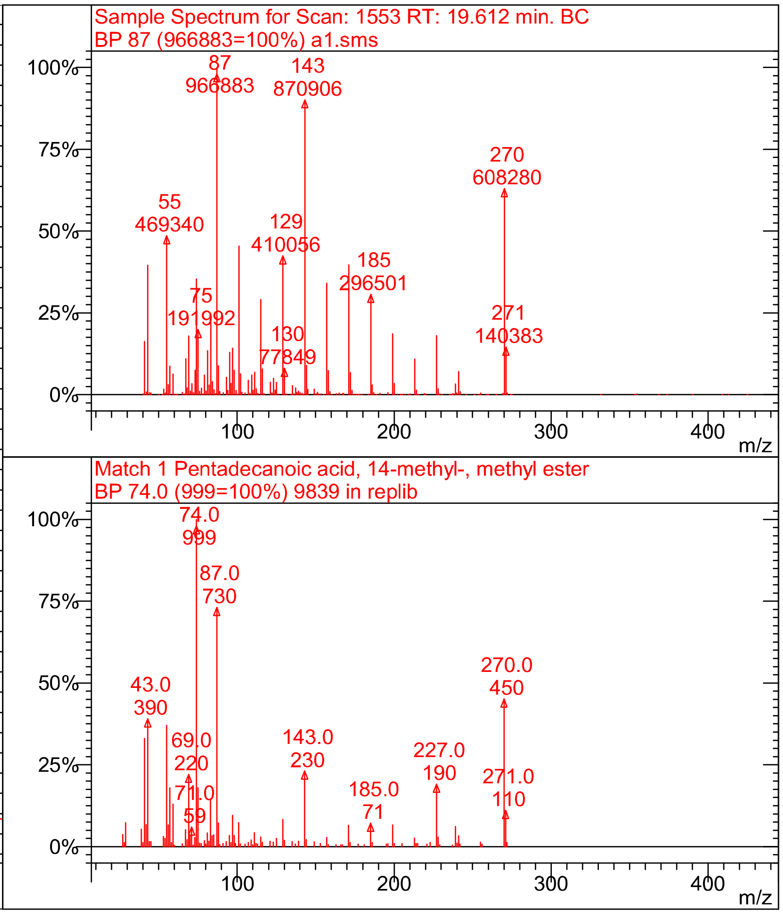

Seg 2, <No Description>, Time: 4.00-30.00

Sample ID:	A1	Operator:	
Instrument ID:	MS Instrument #1	Last Calibration:	None
Measurement Type:	Area	Calibration Type:	External Standard
Acquisition Date:	10/20/2020 1:52 PM	Data File:	d:\201020_ils\a1.sms
Calculation Date:	10/20/2020 5:01 PM	Method:	d:\ms\method\fa.mth
Sample Type:	Analysis		
Inj. Sample Notes:	None		

Compound Information

Peak Name: Benzenepropanoic acid, 3,5-bis(1,1-dimet
Result Index: 22 Peak Number: 22 CAS Number: 6386-38-5 TIC

Identification

Parameter	Specification	Actual	Status
Search Type	Library Search		
Retention Time		19.711 min.	
1st Match Library		mainlib	
1st Match Entry No.		192656	
2nd Match Library		replib	
2nd Match Entry No.		29429	
3rd Match Library		mainlib	
3rd Match Entry No.		192657	
Forward Match	N-F >= 500	725	Pass
Reverse Match		742	

Integration and Quantitation

Parameter	Specification	Actual	Status
Quan Ions	RIC		
RF Used	1.000		
Area	>=5000	507880	Pass
Height		293963	
Amount		507880 Counts	

Match Types: N-F : Normal-Forward

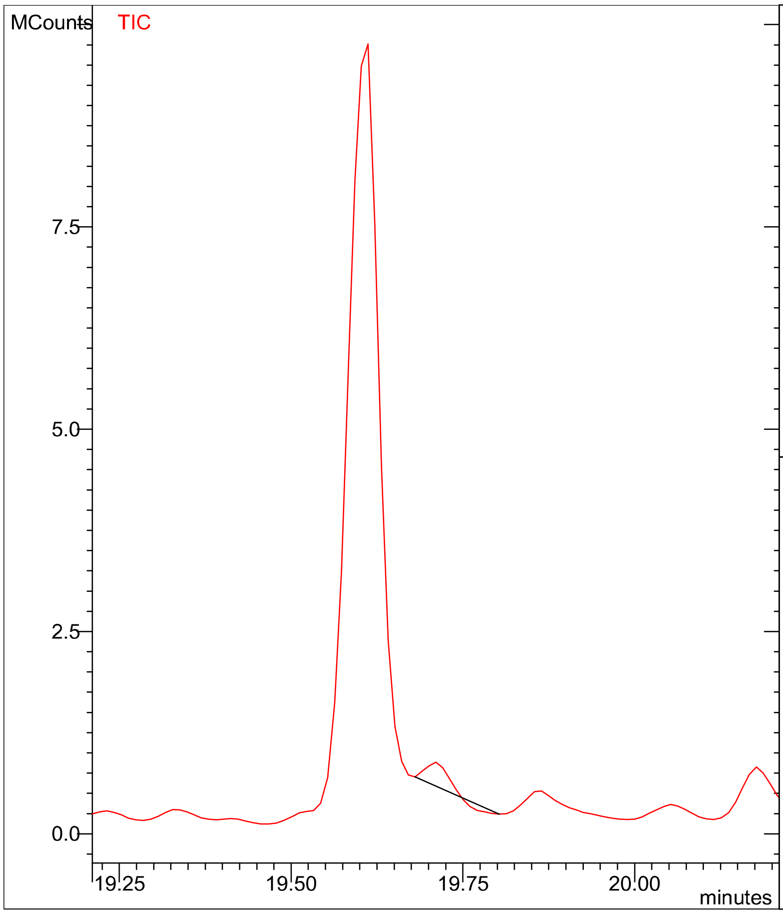

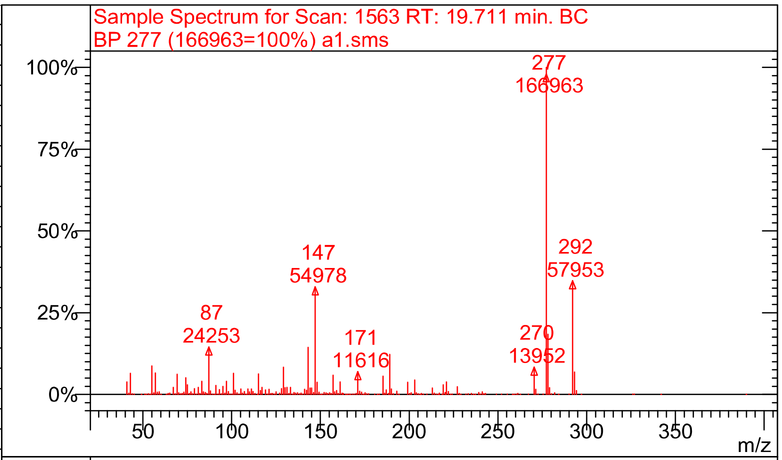

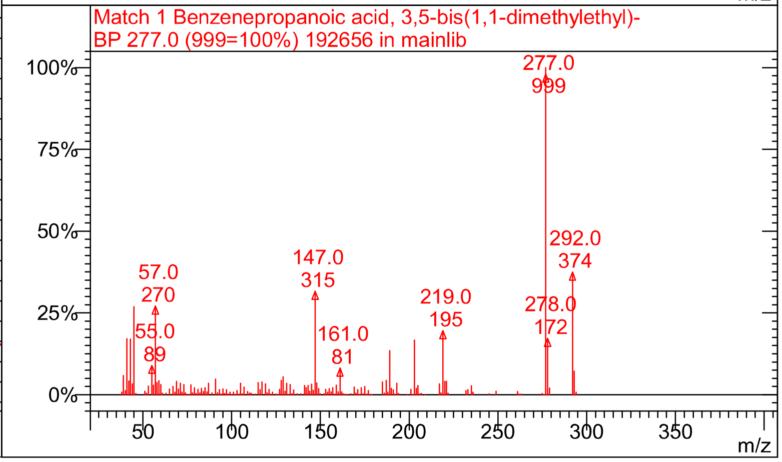

Seg 2, <No Description>, Time: 4.00-30.00

Sample ID:	A1	Operator:	
Instrument ID:	MS Instrument #1	Last Calibration:	None
Measurement Type:	Area	Calibration Type:	External Standard
Acquisition Date:	10/20/2020 1:52 PM	Data File:	d:\201020_ils\a1.sms
Calculation Date:	10/20/2020 5:01 PM	Method:	d:\ms\method\fa.mth
Sample Type:	Analysis		
Inj. Sample Notes:	None		

Compound Information

Peak Name: Tritetracontane
Result Index: 23 Peak Number: 23 CAS Number: 7098-21-7 TIC

Identification

Parameter	Specification	Actual	Status
Search Type	Library Search		
Retention Time		20.177 min.	
1st Match Library		mainlib	
1st Match Entry No.		23579	
2nd Match Library		replib	
2nd Match Entry No.		5811	
3rd Match Library		replib	
3rd Match Entry No.		5842	
Forward Match	N-F >= 500	831	Pass
Reverse Match		831	

Integration and Quantitation

Parameter	Specification	Actual	Status
Quan Ions	RIC		
RF Used	1.000		
Area	>=5000	1.581e+6	Pass
Height		561456	
Amount		1581310 Counts	

Match Types: N-F : Normal-Forward

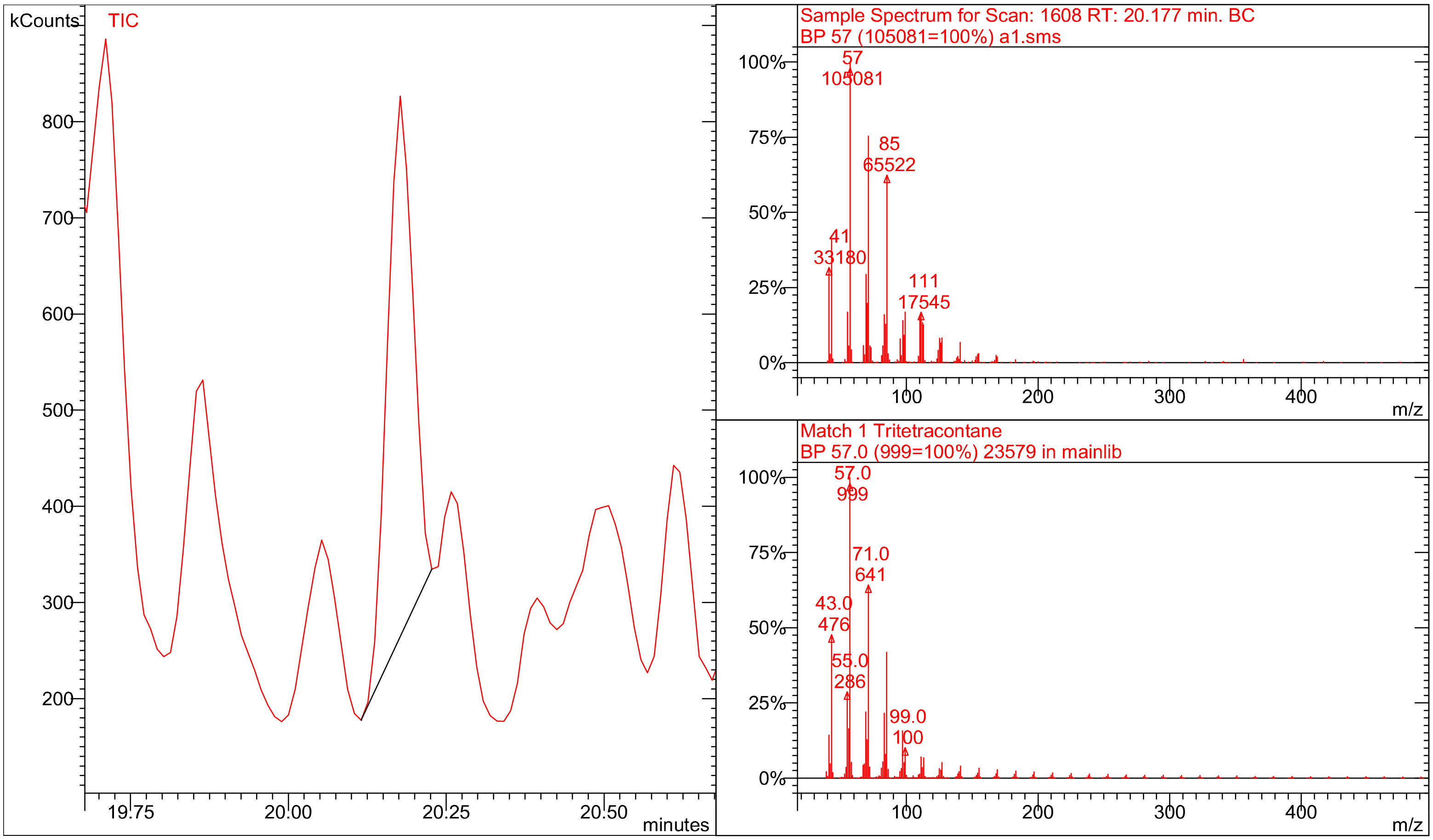

Seg 2, <No Description>, Time: 4.00-30.00

Sample ID:	A1	Operator:	
Instrument ID:	MS Instrument #1	Last Calibration:	None
Measurement Type:	Area	Calibration Type:	External Standard
Acquisition Date:	10/20/2020 1:52 PM	Data File:	d:\201020_ils\a1.sms
Calculation Date:	10/20/2020 5:01 PM	Method:	d:\ms\method\fa.mth
Sample Type:	Analysis		
Inj. Sample Notes:	None		

Compound Information

Peak Name:	Methyl 9-cis,11-trans-octadecadienoate			
Result Index:	24	Peak Number: 24	CAS Number: None	TIC

Identification

Parameter	Specification	Actual	Status
Search Type	Library Search		
Retention Time		23.597 min.	
1st Match Library		mainlib	
1st Match Entry No.		30152	
2nd Match Library		mainlib	
2nd Match Entry No.		30153	
3rd Match Library		mainlib	
3rd Match Entry No.		30156	
Forward Match	N-F >= 500	852	Pass
Reverse Match		854	

Integration and Quantitation

Parameter	Specification	Actual	Status
Quan Ions	RIC		
RF Used	1.000		
Area	>=5000	2.977e+6	Pass
Height		929035	
Amount		2976975 Counts	

Match Types: N-F : Normal-Forward

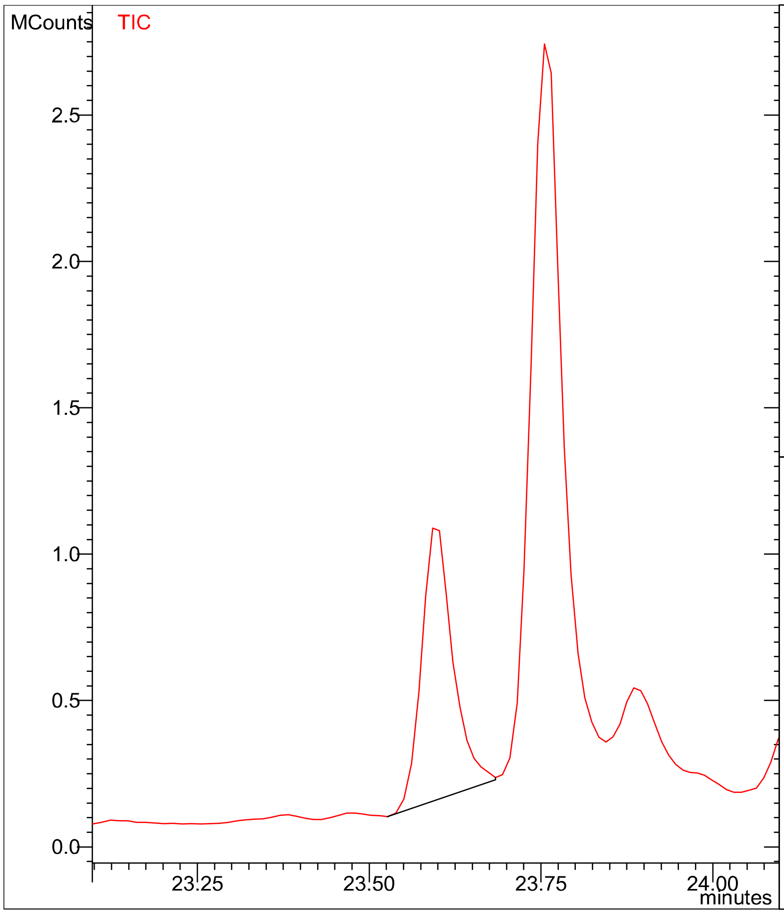

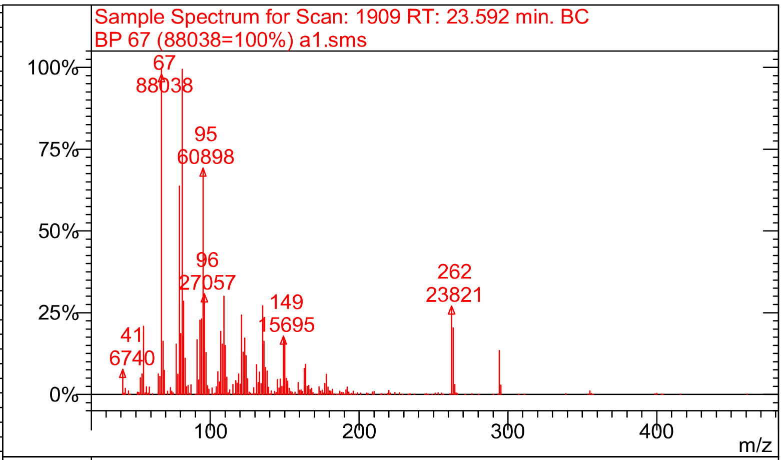

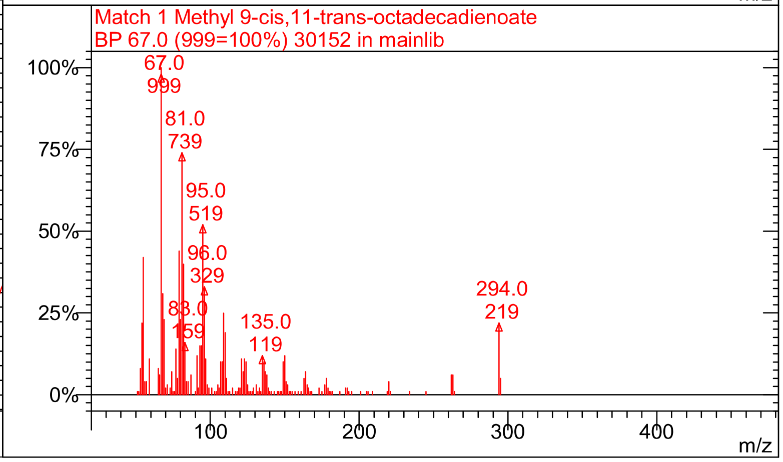

Seg 2, <No Description>, Time: 4.00-30.00

Sample ID:	A1	Operator:	
Instrument ID:	MS Instrument #1	Last Calibration:	None
Measurement Type:	Area	Calibration Type:	External Standard
Acquisition Date:	10/20/2020 1:52 PM	Data File:	d:\201020_ils\a1.sms
Calculation Date:	10/20/2020 5:01 PM	Method:	d:\ms\method\fa.mth
Sample Type:	Analysis		
Inj. Sample Notes:	None		

Compound Information

Peak Name: 9-Octadecenoic acid (Z)-, methyl ester
Result Index: 25 Peak Number: 25 CAS Number: 112-62-9 TIC

Identification

Parameter	Specification	Actual	Status
Search Type	Library Search		
Retention Time		23.758 min.	
1st Match Library		mainlib	
1st Match Entry No.		18902	
2nd Match Library		replib	
2nd Match Entry No.		4732	
3rd Match Library		mainlib	
3rd Match Entry No.		18891	
Forward Match	N-F >= 500	826	Pass
Reverse Match		830	

Integration and Quantitation

Parameter	Specification	Actual	Status
Quan Ions	RIC		
RF Used	1.000		
Area	>=5000	7.822e+6	Pass
Height		2.454e+6	
Amount		7822113 Counts	

Match Types: N-F : Normal-Forward

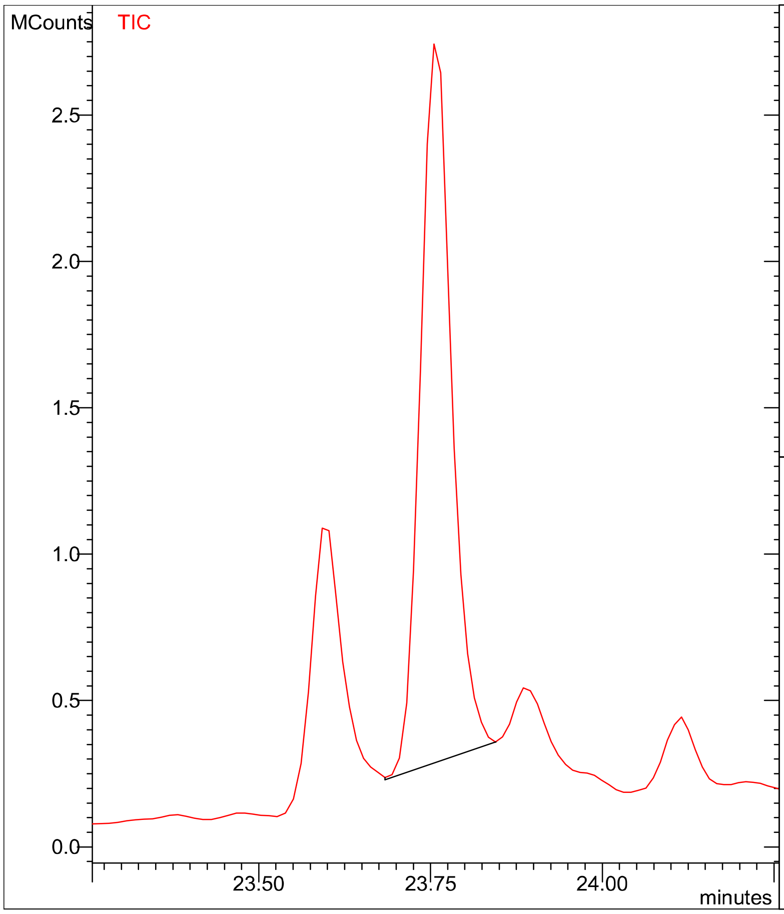

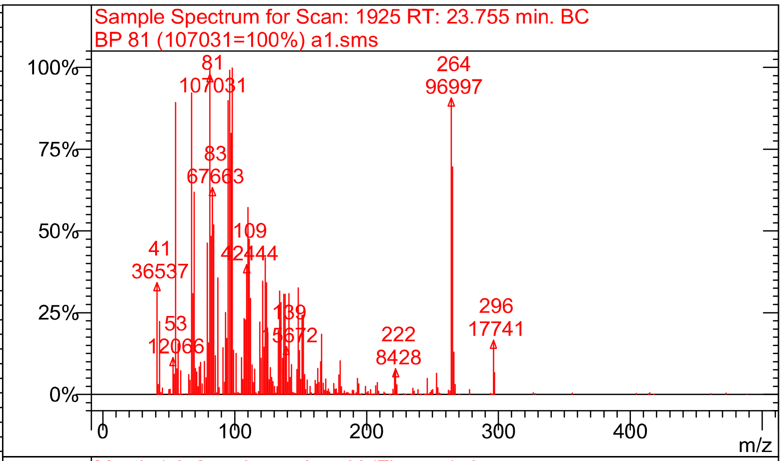

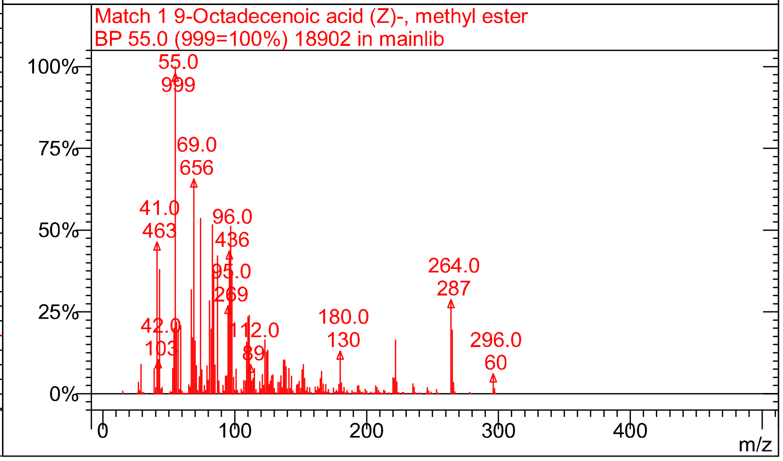

Seg 2, <No Description>, Time: 4.00-30.00

Sample ID:	A1	Operator:	
Instrument ID:	MS Instrument #1	Last Calibration:	None
Measurement Type:	Area	Calibration Type:	External Standard
Acquisition Date:	10/20/2020 1:52 PM	Data File:	d:\201020_ils\a1.sms
Calculation Date:	10/20/2020 5:01 PM	Method:	d:\ms\method\fa.mth
Sample Type:	Analysis		
Inj. Sample Notes:	None		

Compound Information

Peak Name: Methyl stearate
Result Index: 26 Peak Number: 26 CAS Number: 112-61-8 TIC

Identification

Parameter	Specification	Actual	Status
Search Type	Library Search		
Retention Time		24.380 min.	
1st Match Library		replib	
1st Match Entry No.		9840	
2nd Match Library		replib	
2nd Match Entry No.		9838	
3rd Match Library		mainlib	
3rd Match Entry No.		40878	
Forward Match	N-F >= 500	837	Pass
Reverse Match		838	

Integration and Quantitation

Parameter	Specification	Actual	Status
Quan Ions	RIC		
RF Used	1.000		
Area	>=5000	1.882e+7	Pass
Height		5.487e+6	
Amount		18815896 Counts	

Match Types: N-F : Normal-Forward

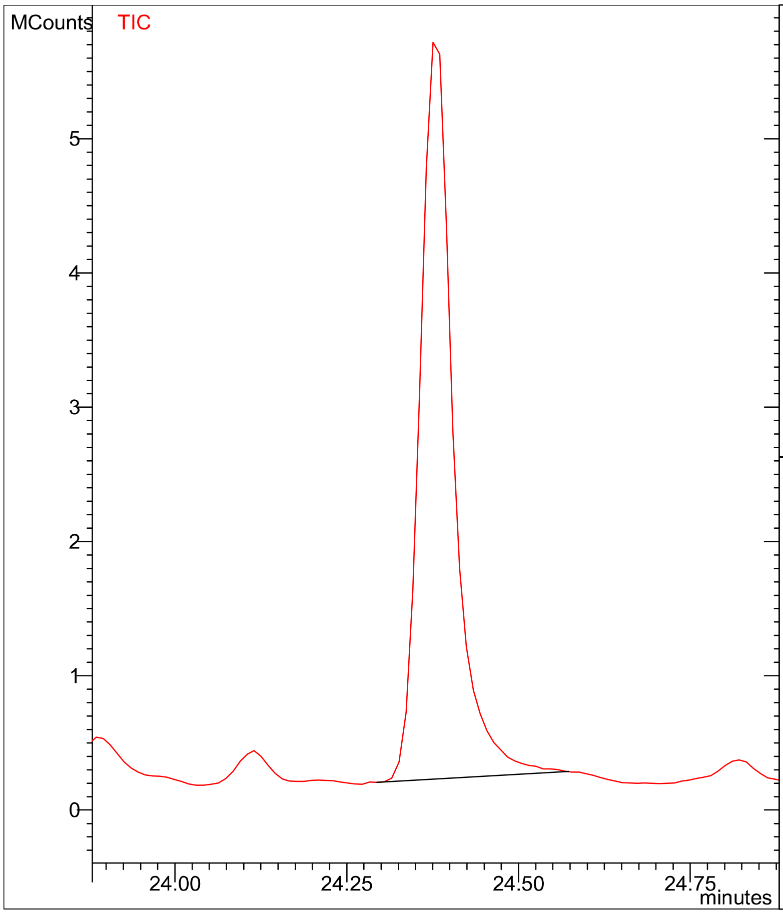

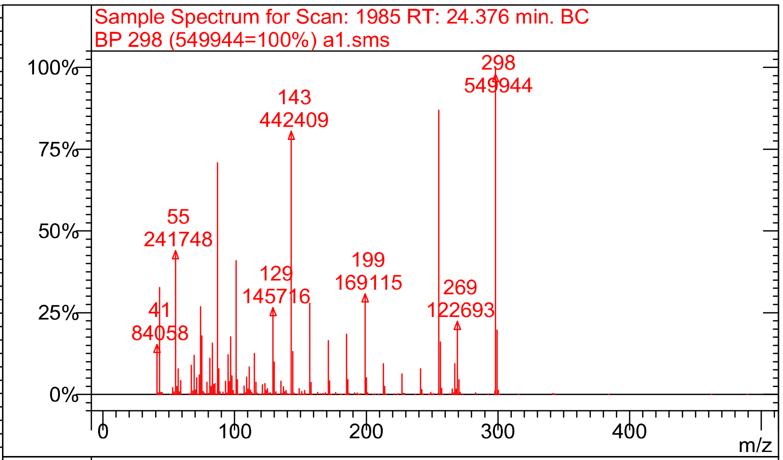

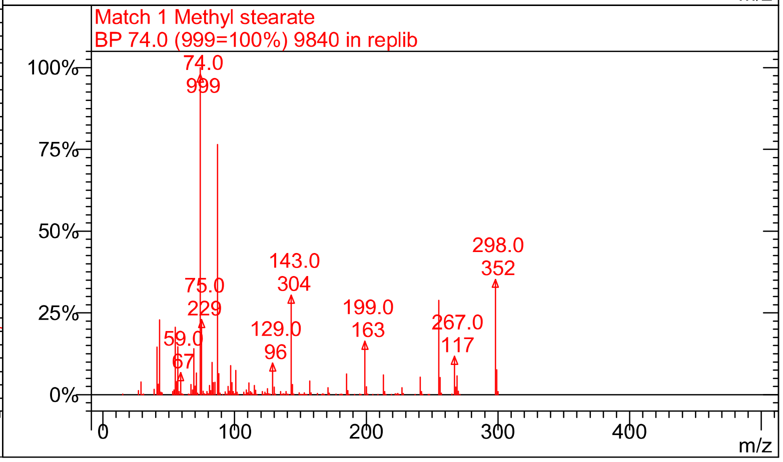

Seg 2, <No Description>, Time: 4.00-30.00

Sample ID:	A1	Operator:	
Instrument ID:	MS Instrument #1	Last Calibration:	None
Measurement Type:	Area	Calibration Type:	External Standard
Acquisition Date:	10/20/2020 1:52 PM	Data File:	d:\201020_ils\a1.sms
Calculation Date:	10/20/2020 5:01 PM	Method:	d:\ms\method\fa.mth
Sample Type:	Analysis		
Inj. Sample Notes:	None		

Compound Information

Peak Name:	Tritetracontane			
Result Index:	27	Peak Number: 27	CAS Number: 7098-21-7	TIC

Identification

Parameter	Specification	Actual	Status
Search Type	Library Search		
Retention Time		25.084 min.	
1st Match Library		mainlib	
1st Match Entry No.		23579	
2nd Match Library		replib	
2nd Match Entry No.		5811	
3rd Match Library		replib	
3rd Match Entry No.		5842	
Forward Match	N-F >= 500	827	Pass
Reverse Match		827	

Integration and Quantitation

Parameter	Specification	Actual	Status
Quan Ions	RIC		
RF Used	1.000		
Area	>=5000	1.603e+6	Pass
Height		359956	
Amount		1603479 Counts	

Match Types: N-F : Normal-Forward

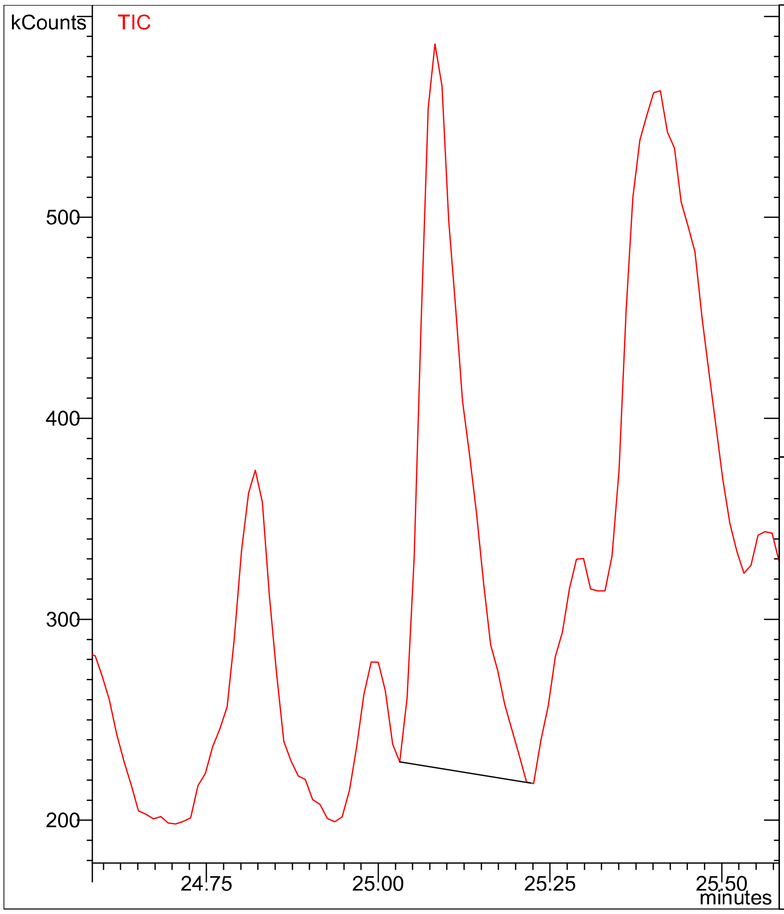

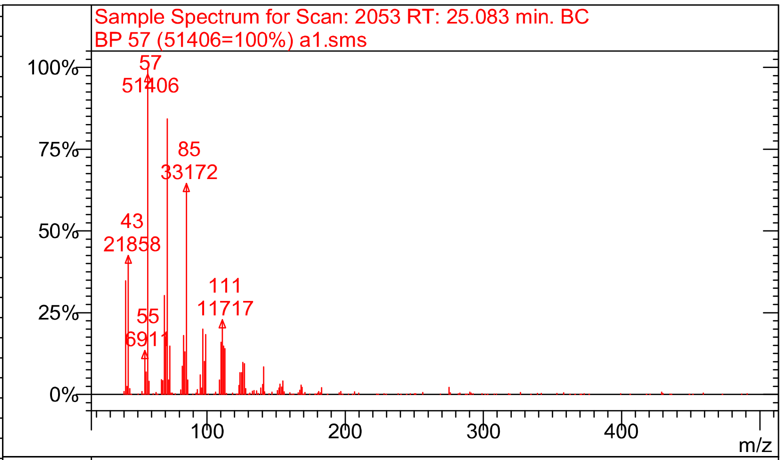

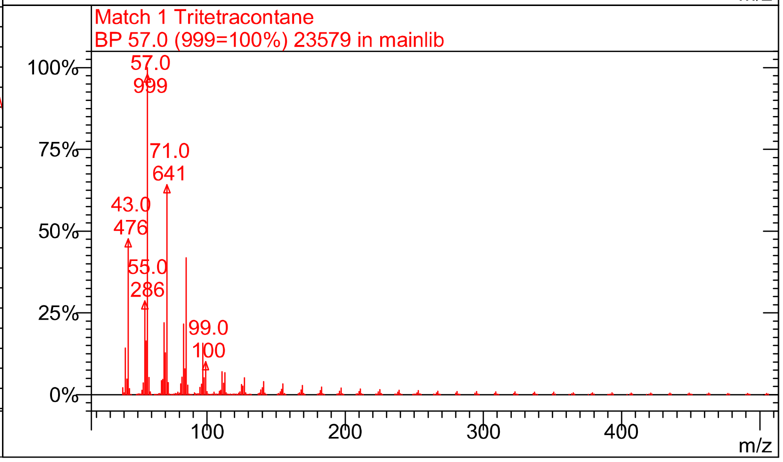

Seg 2, <No Description>, Time: 4.00-30.00

Sample ID:	A1	Operator:	
Instrument ID:	MS Instrument #1	Last Calibration:	None
Measurement Type:	Area	Calibration Type:	External Standard
Acquisition Date:	10/20/2020 1:52 PM	Data File:	d:\201020_ils\a1.sms
Calculation Date:	10/20/2020 5:01 PM	Method:	d:\ms\method\fa.mth
Sample Type:	Analysis		
Inj. Sample Notes:	None		

Compound Information

Peak Name: 5,8,11,14-Eicosatetraenoic acid, methyl
Result Index: 28 Peak Number: 28 CAS Number: 2566-89-4 TIC

Identification

Parameter	Specification	Actual	Status
Search Type	Library Search		
Retention Time		27.249 min.	
1st Match Library		replib	
1st Match Entry No.		10455	
2nd Match Library		mainlib	
2nd Match Entry No.		44574	
3rd Match Library		mainlib	
3rd Match Entry No.		44570	
Forward Match	N-F >= 500	854	Pass
Reverse Match		860	

Integration and Quantitation

Parameter	Specification	Actual	Status
Quan Ions	RIC		
RF Used	1.000		
Area	>=5000	4.938e+6	Pass
Height		1.346e+6	
Amount		4937641 Counts	

Match Types: N-F : Normal-Forward

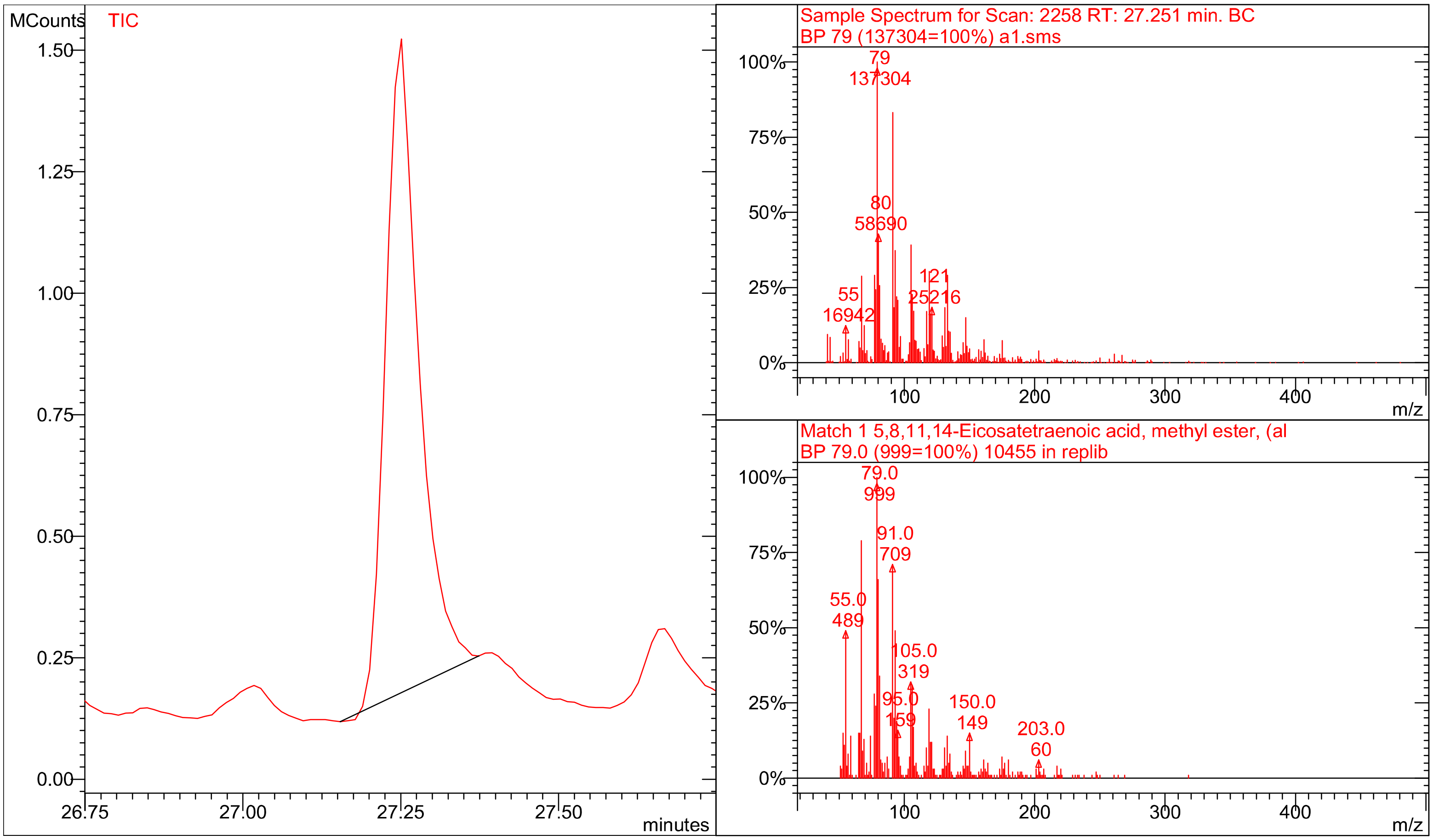

Seg 2, <No Description>, Time: 4.00-30.00
