# Supplementary figures and images for: Malaria parasite heme biosynthesis promotes and griseofulvin protects against cerebral malaria in mice
Source: Nat Commun. 2022 Jul 12;13:4028. doi: 10.1038/s41467-022-31431-z (PMC9276668; doi:10.1038/s41467-022-31431-z)

# Chromatogram Plot

File: d:\201020\_ils\la1.sms

Sample: A1

Scan Range: 1 - 2508 Time Range: 0.00 - 29.98 min.

Operator:

Date: 10/20/2020 1:52 PM

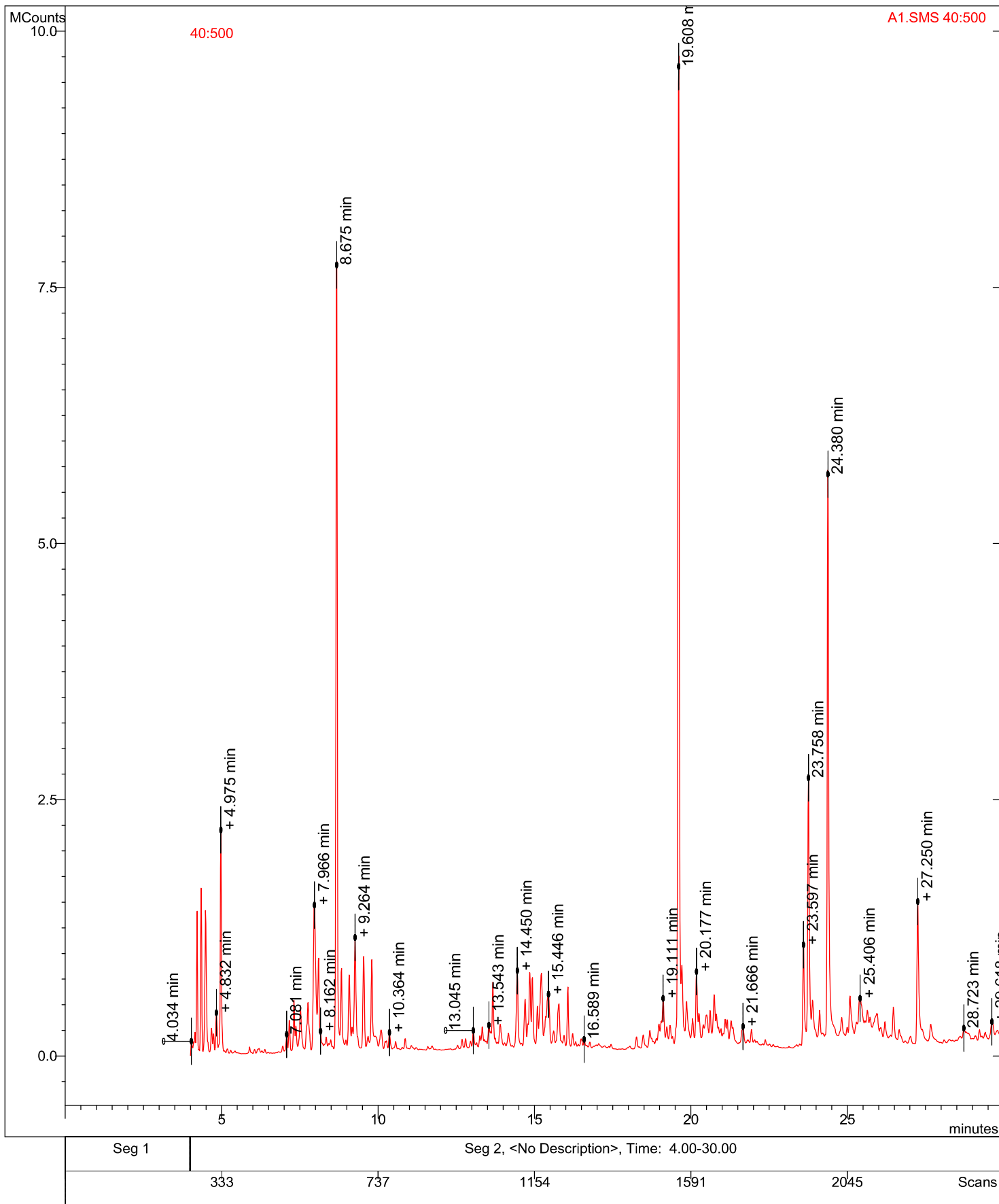

Supplement: Supplementary file 10 — Source Data [file 41467_2022_31431_MOESM10_ESM.zip › Source Data/GCMS for Lipid Analysis/TIC.pdf]
